# MAST: a hybrid Multi-Agent Spatio-Temporal model of tumor microenvironment informed using a data-driven approach

**DOI:** 10.1093/bioadv/vbac092

**Published:** 2022-12-05

**Authors:** Giulia Cesaro, Mikele Milia, Giacomo Baruzzo, Giovanni Finco, Francesco Morandini, Alessio Lazzarini, Piergiorgio Alotto, Noel Filipe da Cunha Carvalho de Miranda, Zlatko Trajanoski, Francesca Finotello, Barbara Di Camillo

**Affiliations:** Department of Information Engineering, University of Padova, 35131 Padova, Italy; Department of Information Engineering, University of Padova, 35131 Padova, Italy; Department of Information Engineering, University of Padova, 35131 Padova, Italy; Department of Information Engineering, University of Padova, 35131 Padova, Italy; Department of Information Engineering, University of Padova, 35131 Padova, Italy; Department of Information Engineering, University of Padova, 35131 Padova, Italy; Department of Industrial Engineering, University of Padova, 35131 Padova, Italy; Department of Pathology, Leiden University Medical Center, 2300 RC Leiden, The Netherlands; Biocenter, Institute of Bioinformatics, Medical University of Innsbruck, 6020 Innsbruck, Austria; Biocenter, Institute of Bioinformatics, Medical University of Innsbruck, 6020 Innsbruck, Austria; Institute of Molecular Biology, University Innsbruck, 6020 Innsbruck, Austria; Digital Science Center (DiSC), University Innsbruck, 6020 Innsbruck, Austria; Department of Information Engineering, University of Padova, 35131 Padova, Italy; Department of Comparative Biomedicine and Food Science, University of Padova, 35020 Padova, Italy

## Abstract

**Motivation:**

Recently, several computational modeling approaches, such as agent-based models, have been applied to study the interaction dynamics between immune and tumor cells in human cancer. However, each tumor is characterized by a specific and unique tumor microenvironment, emphasizing the need for specialized and personalized studies of each cancer scenario.

**Results:**

We present MAST, a hybrid Multi-Agent Spatio-Temporal model which can be informed using a data-driven approach to simulate unique tumor subtypes and tumor–immune dynamics starting from high-throughput sequencing data. It captures essential components of the tumor microenvironment by coupling a discrete agent-based model with a continuous partial differential equations-based model.

The application to real data of human colorectal cancer tissue investigating the spatio-temporal evolution and emergent properties of four simulated human colorectal cancer subtypes, along with their agreement with current biological knowledge of tumors and clinical outcome endpoints in a patient cohort, endorse the validity of our approach.

**Availability and implementation:**

MAST, implemented in Python language, is freely available with an open-source license through GitLab (https://gitlab.com/sysbiobig/mast), and a Docker image is provided to ease its deployment. The submitted software version and test data are available in Zenodo at https://dx.doi.org/10.5281/zenodo.7267745.

**Supplementary information:**

Supplementary data are available at *Bioinformatics Advances* online.

## 1 Introduction

The tumor microenvironment (TME) is composed of multiple interacting cells, including cancer cells, infiltrated immune cells [e.g. T cells, natural killer (NK) cells, dendritic cells (DCs) and macrophages (Ms)], mesenchymal stromal cells or fibroblasts (Plava *et al.*, 2019). The composition of the TME is specific and unique for each cancer and evolves in time and space in response to resources, stresses and (epi)genetic mutations. Indeed, the TME is made up of different cell types and cell states that correspond to distinct activation or metabolic profiles. These entities communicate with each other through physical interactions or by releasing specific molecular signals (Baruzzo *et al.*, 2022) that can induce cell motility, proliferation, death, etc. As a consequence, the composition and organization (e.g. tumor core versus Periphery) of the TME at the initial, middle or late stages of tumor progression can significantly differ.

Cell–cell interactions in the TME alter cell biology, ultimately influencing disease progression to the point that various heterologous interactions in the TME are now considered hallmarks of cancer biology (Hanahan and Coussens, 2012). In this context, it is of paramount importance to characterize different TME properties and how they affect tumor progression. Integrative and quantitative modeling of the TME, although simplifying many aspects of reality, represents a means toward a better understanding of the dynamics of cancer development, the phenotypes characterizing different TMEs and even generates future outcome prediction to design therapies based on *in silico* observations (Peskov *et al.*, 2019).

In the literature, there are two main simulation approaches (Norton *et al.*, 2019). The top-down modeling approaches, such as temporal ordinary differential equations or spatio-temporal partial differential equations (PDEs), are meant to model nutrient or signaling molecules concentrations, without focusing on the behavior of each single entity. However, the complex interactions among communication molecules and the details about metabolisms are far to be completely understood in biology and, therefore, only a few system components can be characterized (Clarke and Fisher, 2020; Thomas *et al.*, 2016).

In contrast, the bottom-up approach focuses on single-cell entities and their individual interactions with the aim of identifying which emergent properties may result from their collective behavior. For example, agent-based models (ABMs) are discrete models where heterogenous entities, i.e. agents, are defined, as well as their behaviors, attributes and interactions following predefined rules (Bonabeau, 2002). Such models better reflect the stochastic, spatial-dependent and heterogenous nature of biology but require more computational power relative to top-down approaches.

The hybrid approach represents a powerful way to combine the above strategies allowing to describe the different level and scale of biological systems choosing the most suitable modeling approach, at the cost of increasing computational and methodological complexity while integrating such approaches. The combination of the two modeling frameworks might help describing the physical reality at both cellular and communication/metabolism molecules level in the context of TME.

In the recent years, several quantitative models of increasing complexity have been developed to represent different mechanisms of tumor–immune microenvironment, such as tumor mechanobiology, vasculature, lymphatics and immunotherapy (Norton *et al.*, 2019). For example, Frascoli *et al.* (2017) suggested a stochastic ABM coupled with delay differential equations to mimic cell mobility and cellular adhesion to the extracellular matrix in tumor site. Norton *et al.* (2018) combined three ABMs of tumor–immune system, angiogenesis and tumor-associated stroma to investigate the stroma cell influence on tumor progression. Other ABMs and hybrid models focused on modeling immune escape mechanisms: (i) loss of antigenicity by avoiding immune recognition through antigen presentation thereby reducing tumor-specific immune response; (ii) loss of immunogenicity by expressing inhibitory immune checkpoint molecules, such as PD-L1; and (iii) inducing an immunosuppressive microenvironment (Beatty and Gladney, 2015). In this context, Kather *et al.* (2017) presented a stochastic ABM of immune–tumor–stromal interactions in human colorectal cancer (CRC) to simulate different immunological phenotypes, while Gong *et al.* (2017) proposed a hybrid model of tumor–immune interaction to simulate tumor growth and immune checkpoint inhibitor treatments, targeting the PD-1/PD-L1 axis.

**Fig. 1.**
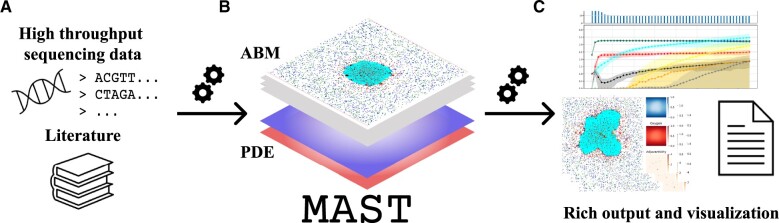
Schematic representation of MAST, a hybrid multi-ABM of tumor–immune system. (**A**) MAST can be informed through a data-driven approach using several sources of information to model unique characteristics of the TME in a tumor. (**B**) It couples a discrete ABM, which simulates tumor–immune system dynamics in the TME, and a continuous PDEs-based model to simulate nutrient diffusion from vessels. (**C**) MAST provides tabular and graphical outputs in order to analyze spatio-temporal evolution of *in silico* tumor growth simulation

In this work, we developed MAST, a hybrid Multi-Agent Spatio-Temporal model of human solid tumor tissue that includes an ABM, simulating tumor and immune cell interactions, and a PDE-based model to simulate nutrients diffusion from blood vessels through the tumor tissue (see Fig. 1). The use of the two modeling frameworks allows to couple discrete and continuous modeling and capture essential elements of the TME, i.e. cells interaction and nutrients availability. With respect to previous works, MAST introduces three main contributions: (i) it models nutrient diffusion from vessels and cell dynamics in response to nutrient availability; (ii) it models immune escape mechanisms through the accumulation of mutations during the disease course, leading to spatial and temporal heterogeneity of tumor subpopulations; and (iii) it introduces a data-driven approach to inform model parameters, thus simulating specific characteristics of a real TME, from high-throughput data.

In particular, we informed MAST with literature data, as well as genomics and transcriptomics data from bulk and single-cell sequencing technologies, yielding knowledge on tumor mutation rate (Huang and Lee, 2022), percentage of cell types in the TME (Sturm *et al.*, 2019) and loss of immunogenicity, to model the TME of human CRC. The model faithfully recapitulates emergent behavior and predicts tumor progression in four subtypes of CRC tissue, i.e. Consensus Molecular Subtypes (CMS) (Guinney *et al.*, 2015). The model was then used to investigate the effect of different tumor conditions on colorectal tumor growth.

## 2 Methods

MAST allows modeling interactions among tumor cells, necrotic cells, cancer-associated fibroblasts (CAFs), NKs, cytotoxic T lymphocytes (CTLs), regulatory T cells (Tregs) and DCs. It simulates tumor-cell mutations and acquisition of new properties and antigens, toward which the CTLs specialize, including: changes in proliferation/survival fitness (Stratton *et al.*, 2009; Vicens and Posada, 2018); loss/acquisition of antigenicity; loss of immunogenicity; and ability to shape immunosuppressive microenvironment (cancer cells might evade the immune system (IS) by releasing signaling molecules that locally repel IS cells) (Beatty and Gladney, 2015; Tang *et al.*, 2020). Moreover, MAST models nutrient availability in the tissue which drives proliferation and migration in the TME.

### 2.1 Model design and implementation

In the ABM, each agent: (i) autonomously controls its actions; (ii) lives in a physical space, i.e. a 2D grid representing the tissue, where it interacts in a timely and probabilistic manner with other agents and the surrounding environment; and (iii) is characterized by a specific metabolism, i.e. nutrients consumption, and thus competes for nutrients.

The neighborhood that allows an agent to sense surrounding changes is defined according to Moore’s definition on a 2D lattice as composed of the agent itself and the eight closest, surrounding cells. Two classes of agents, namely cancer-related and immune-related, representing the main categories of cells in TME are modeled. For simplicity, we use agent and cell as synonyms.

#### 2.1.1 Cancer-related agents

Cancer is a disease characterized by dysfunctional apoptosis, abnormal cell division and accumulation of mutations that give rise to TME heterogeneity, both in terms of spatial properties and temporal evolution (Dagogo-Jack and Shaw, 2018). During disease, tumor cells accumulate mutations that might result in different subpopulations of malignant cells with distinct mutational profiles and different abilities to survive.

TME also contains tumor stromal cells, which include fibroblasts and mesenchymal stromal cells. In this environment, CAFs can differentiate and promote tumor growth, angiogenesis and shape an immunosuppressive environment (Plava *et al.*, 2019). In MAST, we simulated CAFs and cancer cells with different mutational burden, also modeling cell division, cell death and cell movement.

*Cell division*: We assume cancer cell duplication probability depends on nutrient concentration and on cancer cell mutation fitness parameter. The probability of a cell to duplicate is computed as:

(1) $$p_{\mathrm{dupl}}(i,j)=1-\exp\left[-{\left(\frac{N(i,j)}{\frac{\theta_{\mathrm{dupl}}}{1\;+\;\#\mathrm{neighbCAFs}(i,j)\;\cdot \;\theta_{{\mathrm{dupl}}_{\mathrm{stroma}}}}}\right)}^{2}\right],$$

where N(i,j) represents the concentration of substances useful for cell duplication (e.g. glucose) in grid position (i,j) and $\theta_{\mathrm{dupl}}$ is a parameter related to the mutational status of the cell, so that there might be cells duplicating at higher rate than others at the same nutrient concentrations, depending on the cell fitness. To mimic promotion of cancer cell duplication by CAFs, parameter θ_*dupl*_ in Equation (1) is divided by 1 plus the number of CAFs cells in the Moore neighborhood multiplied by parameter θ_*dupl_stroma*_. When dividing, daughter cancer cells inherit all the mutations of the original cell.

*Cell death*: Cancer cell agents can die by IS attack, as described below, or by lack of nutrients. Even in this latter case, the probability of death depends on both nutrient concentration and mutational status of the cell as follow:

(2) $$p_{\mathrm{necr}}(i,j)=\exp\left[-{\left(\frac{M(i,j)}{\theta_{\mathrm{necr}}}\right)}^{2}\right],$$

where M(i,j) represents the concentration of substances useful for cell maintenance (e.g. oxygen) and θ_*necr*_ is a parameter related to the mutational status of the cell, so that there might be cells that survive easily than other in an environment poor of these substances, depending on the cell fitness. To mimic immunogenic cell death, molecules enhancing immune cell recruitment in the area are released after cancer cell death (Fucikova *et al.*, 2020). If cancer cells die by lack of nutrients, they become necrotic remaining in the environment for a certain time and being able to recruit DC agents (Sauter *et al.*, 2000). We did not explicitly model apoptosis.

*Mutation*: If a mutation occurs, it can be of different types, each with a certain rate depending on the tumor type. Here, we are interested in modeling (epi)genetic mutations that can give rise to: (1) loss or acquisition of antigenicity, i.e. new tumor antigens (proteins capable of inducing a tumor-specific immune response); (2) loss of immunogenicity by expressing inhibitory immune checkpoint molecules, such as PD-L1 molecule; (3) release signaling molecules that locally repel immune cells; and (4) increased or decreased duplication/survival fitness which is mimicked by different values of parameters θ_*dupl*_ and θ_*necr*_ in Equations (1) and (2). In our simulation, if a cancer cell undergoes a mutation of Type (1), a new antigen is randomly sampled from a set of possible mutations, and its mutational status is updated; thus, the cell lineage accumulates mutations during the simulation. To mimic different TME properties, Types (1)–(4) mutations can occur at different probabilities (see Supplementary Table S1).

*Movement*: In MAST, cancer cells can move to a position in the Moore neighborhood with the following probability.

(3) $$p_{\mathrm{move}}(i,j)=1-\exp\left[-{\left(\frac{M(i,j)}{\frac{\theta_{\mathrm{move}}}{1\;+\;\#\mathrm{neighbCAFs}(i,j)}}\right)}^{2}\right].$$

We assume that cancer cells have low migration probability which depends on both θ_*move*_ parameter and on nutrient concentration M(i,j). We implement this feature to allow simulating different settings depending on the tumor type. Moreover, to mimic promotion of cancer cell diffusion by CAFs, parameter θ_*move*_ in Equation (3) is divided by 1 plus the number of CAFs cells in the Moore neighborhood.

*CAFs differentiation*: CAFs are thought to differentiate from normal tissue, and appear in the simulation after a long-lasting inflammation. CAF agent is modeled so to promote tumor growth by augmenting proliferation of the cancer cells, and tumor diffusion by augmenting tumor-cell movement, as described in Equations (1) and (3) (Joshi *et al.*, 2021). To allow different biological scenarios to be modeled, CAFs may allow a different degree of permeability, behaving as obstacles or promoters of movement for tumor cells. In addition, CAF agents are modeled as immunosuppressive cells by inhibiting immune cell recruitment as explained in the next paragraph. They can die by lack of nutrients with the same probability of a cancer cell [see Equation (2)].

#### 2.1.2 Immune system agents

IS constitutes a strong defense against cancer development, catching and eliminating cells that undergo malignant transformation. The way the IS acts to provide immune surveillance is wide and complex; we refer to proper literature to describe it (Hawse and Morel, 2014; Martínez-Lostao *et al.*, 2015). However, only for the purpose of introducing cell agents in MAST, we summarize here its main mechanisms.

Immune response rises from a coordinated action of multiple cell types and molecules. NKs, DCs and Ms are initially recruited in the TME and release cytokines and chemokines that initiate the inflammation. DCs and Ms are also called antigen presenting cells because they phagocyte cellular material including cancer cells, process and present their antigens, within the lymph nodes, to T cells that eventually get primed against tumor antigens.

T cells maturate in different types. CTLs can mount a strong immune response by killing their tumor-cell targets through antigen recognition, while Treg cells have an immunosuppressive function, that maintains tolerance to self-antigens and prevent autoimmunity when inflammation is long-lasting.

Within MAST, NKs, DCs, CTLs and Tregs are explicitly modeled and can be recruited, move, attack, die or disappear from the grid space.

*Recruitment*: In MAST, immune cells are recruited in the tissue with probability increasing with the adjuvanticity signal:

(4) $$p_{\mathrm{recruit}}\left(i,j\right)=\exp\left[-\left(\frac{1}{{\mathrm{adjuvanticity}}_{\mathrm{neigh}}\left(i,j\right)}\right)\right],$$

where ${\mathrm{adjuvanticity}}_{\mathrm{neigh}\;}$ represents the sum of adjuvanticity signals in the Moore neighborhood of cell (i,j). In particular, adjuvanticity is defined as a proxy of the global IS enhancing signal, resulting from the release of molecules promoting immune recruitment (e.g. chemokine) and molecules release by cancer cells and promoting immune suppression. In particular, adjuvanticity signal is increased when a cancer cell dies by lack of nutrients or by IS attack, while it is decreased when cancer cells acquire mutations of Type (3) or immunosuppressive cells (i.e. Tregs and CAFs) are in the neighborhood.

The probability of recruiting specifically NKs, DCs and CTLs is obtained by multiplying *p_recruit_* in Equation (4) by a specific parameter, which reflects their proportions with respect to the total number of IS cells. Differently from other IS cells and consistently with the observation that Tregs are attracted in the domain when the inflammation is long-lasting (Greten and Grivennikov, 2019), Treg agents are first recruited when a CTL becomes inactive after a certain number of attacks, with probability TREG_reclut_par, and act as support agents promoting the recruitment of other Tregs and CAFs with probability described in Equations (5) and (6). Differently from Equation (4), Tregs and CAFs recruitment depends on both the number of Tregs and CAFs in the Moore neighborhood (i.e. ${\mathrm{inhibitory}}_{\mathrm{neigh}}$ parameter) and their corresponding recruitment parameters (i.e. inhib_TREG_recruit_par and inhib_CAF_recruit_par).

(5) $$p_{TREG_{\mathrm{recruit}}}\left(i,j\right)=\exp\left[-\left(\frac{\mathrm{inhib}\_\mathrm{TREG}\_\mathrm{recruit}\_\mathrm{par}}{{\mathrm{inhibitory}}_{\mathrm{neigh}}\;(i,j)\;}\right)\right].$$

(6) $$p_{CAF_{\mathrm{recruit}}}(i,j)=\exp\left[-\left(\frac{\mathrm{inhib}\_\mathrm{CAF}\_\mathrm{recruit}\_\mathrm{par}}{{\mathrm{inhibitory}}_{\mathrm{neigh}}\;(i,\;j)}\right)\right].$$

*Movement*: When cancer cells are not in the neighborhood, immune cells can move to new positions with probability proportional to the adjuvanticity signal. To mimic the fact that T cells and NKs are small and tend to move quickly (Vesperini *et al.*, 2021), we allow them to move in a neighborhood with a bigger radius at each iteration.

*Attack*: When cancer cells are in the neighborhood, DCs process cancer cell antigens and promote the recruitment of antigen-specific T cells in the domain. CTLs attack only when specific antigens are recognized and not many Treg cells are in their neighborhood. Specifically, CTLs have a different killing probability depending on cancer cell mutational status (e.g. PD-L1+/−). In particular, when PD-L1-like mutation is acquired by a tumor cell, the killing probability drops to mimic inhibitory regulation through immune checkpoint molecules (Huang *et al.*, 2019). Consistently with the biological knowledge, CTLs can kill multiple target cells (Wiedemann *et al.*, 2006). After a certain number of attacks, they become inactive and, as explained above, can recruit Treg in their position at this stage, modeling a long-lasting inflammation. Differently from CTLs, NKs are not antigen-specific, and, in MAST, they always attack cancer cells in the neighborhood with a probability of killing which is lower (Cerignoli *et al.*, 2018). If they succeed, then they die and the adjuvanticity signal is locally increased, attracting other immune cells.

*Antigen specificity of CTLs*: If a cancer cell is killed by an NK cell or encounter a DC, then the cancer cell-specific antigens are processed. If the antigen was previously met, antigen-specific T cells are immediately recruited in the environment. Otherwise, antigen-specific T-cell activation is promoted and they will be recruited in the environment with a certain delay, so to mimic their activation in the lymph nodes.

#### 2.1.3 PDE model

PDEs are used to model nutrient diffusion from their source (vessels) and to model nutrient consumption within the TME environment. The nutrient concentration C in a certain time instant t in a certain position of the 2D grid (x,y) is computed by solving the following equation at steady state:

(7) $$\frac{\partial C\left(x,y,t\right)}{\partial t}=D_{c}\;\nabla^{2}C\left(x,y,t\right)-k_{i}\;I\left(x,y,t\right)\;C\left(x,y,t\right)-\;k_{t}\;T\left(x,y,t\right)\;C\left(x,y,t\right),$$

where $D_{c}$ is the nutrient diffusion coefficient, $I\left(x,y,t\right)\;\mathrm{and}\;T(x,y,t)\;$are the spatial distributions at time *t* of IS and cancer-related agents, respectively, and $k_{i}$ and $k_{t}$ are the corresponding consumption rates. We consider two main types of nutrients: those that are essential for cell division (denoted as *N*), and those that are essential for cell survival (denoted as *M*). For nutrients, we use the average glucose diffusion coefficient in tissues (∼1 mm^2^/s) (Carvalho *et al.*, 2017), and thus resolve PDE at a steady state. Since other substances important for cell survival are faster, solution at a steady state is appropriate also for this latter. Normalized nutrient concentrations at blood vessels are assumed equal to 1 arbitrary unit (au).

#### 2.1.4 Mast simulation cycle

Initially, a 2D grid of dimension W×H is defined together with the type of cells occupying each grid position, based on the user-specified parameters. Considering the average grid cell size of ∼14.9 µm (Kather *et al.*, 2017), a lattice of 200 × 200 cells can be used to mimic around 0.09 cm^2^ of tissue corresponding to 40 000 cells. Blood vessels are positioned upstream and downstream of the domain (first and last row of the grid).

At the beginning of the simulation, a fixed number of monoclonal tumor cells are placed in the center of the grid so that a specific and unique antigen setting initially characterizes cancer cells, whereas a fixed percentage of NKs and DCs with respect to the total number of cells are placed in random positions. Upon initialization, at each time-step, MAST first updates the concentrations of nutrients (using the PDE model) and then lets agents act (using the ABM model), sampling for each agent in the grid the action to be done (see Fig. 1), performing a random grid scanning. One time-step t in the simulation corresponds to 6 h, that is the average time for immune cell death (Breart *et al.*, 2008) and we considered cells to divide in ∼24 h (i.e. 4 time-steps) (Kather *et al.*, 2017).

Additional information about model implementation, input and output is provided in Supplementary Section S1, Figures S1–S4 and Tables S1 and S2.

### 2.2 Data-driven strategy to inform and assess MAST

To assess the predictions of the model, we emulate the analysis workflow performed in Kather *et al.* (2017). First, we showed that our model can reproduce key features of real-case biological scenarios of human CRC. Model outcomes in the CRC scenarios were validated using clinical data of the TCGA patient cohort and available biological knowledge on CRC CMS. Specifically, four different TMEs were simulated based on CMS classification: CMS1 (microsatellite instability immune), CMS2 (canonical), CMS3 (metabolic) and CMS4 (mesenchymal) (Guinney *et al.*, 2015). A brief characterization of CMS is provided in Supplementary Section S2 and Figure S5. Second, we investigated the effect of varying different tumor conditions on tumor progression, analyzing the characteristics of resulting TMEs and their agreement with current literature. While some parameters do not change in different TMEs, others can be specified to model unique characteristics of the TME.

CMS simulation scenarios were defined exploiting the data-driven approach that characterizes MAST (see Fig. 1A), i.e. setting some simulation parameters based on high-throughput sequencing data (see Table 1 and Supplementary Table S3). In detail, tumor mutational burden (TMB) can be derived from bulk whole-genome sequencing experiments (bulk DNA-seq), while cell fraction and expression levels of inhibitory immune checkpoint genes can be estimated from bulk and single-cell RNA sequencing (scRNA-seq) data. Here, we informed our model using CRC publicly available data from TCGA database and Samsung Medical Center (SMC) dataset of Lee *et al.* (2020), as explained in Supplementary Section S3 and Figure S6.

**Table 1.** Data-driven setting of some MAST parameters across CMS

Parameter	CMS1	CMS2	CMS3	CMS4	Source	Index
tum_newantigen_rate	High	Low	Low	Low	DNA-seq	TMB
tum_pdlp_rate	High	Low	High	High	scRNA-seq	Gene expression
tum_adjchange_rate	Low	High	Mid	Low	RNA-seq	Cell fraction
inhib_TREG_recruit_par	High	High	High	Low	RNA-seq	Cell fraction
inhib_CAF_recruit_par	High	High	High	Low	RNA-seq	Cell fraction
tum_ncons	Low	Low	High	Low	literature	/

*Bulk whole-genome sequencing*: TMB measures the total number of nonsynonymous mutations per coding area of a sample genome (Mut/Mb) and was estimated for each TCGA patient analyzing bulk whole-genome data (Thorsson *et al.*, 2018). Given the mutational potential to generate neoantigens (Braun *et al.*, 2016), TMB index was used to set the mutational probability related to loss/acquisition of antigenicity (parameter tum_newantigen_rate set *high* or *low* in Table 1) following the trend of TMB distribution in the data across CMS (Supplementary Fig. S7), also confirmed by literature (Kather *et al.*, 2018).

*Bulk RNA sequencing*: Cell fractions were estimated from TCGA bulk RNA-seq data using a bioinformatics approach based on EPIC (Racle *et al.*, 2017) and quanTIseq (Finotello *et al.*, 2019), further refined leveraging a consensus of several deconvolution methods accessible through the immunedeconv R package (Sturm *et al.*, 2019), as explained in Supplementary Section S3 and Figure S6. CAF and Treg cell proportion estimates were used to tune recruitment probabilities of the two cell types, i.e. Equations (5) and (6) (parameters inhib_CAF_recruit_par and inhib_TREG_recruit_par in Table 1). Moreover, immune cell proportion, computed as the sum of all immune-related cell type fractions, was used to set parameter tum_adjchange_rate, as higher values of immune cell proportions mean a lower probability of cancer cells of creating an immunosuppressive environment and thus locally repel the IS cells. The setting of these parameters to different levels across the CMS, reported in Table 1, aims to mimic the trend of cell fraction proportions showed by CRC TCGA data (see Supplementary Figs S8 and S9).

*Single-cell RNA sequencing*: The average expression level of inhibitory immune checkpoint genes, i.e. *CD274* (PD-L1)-like genes (complete list of genes is available in Supplementary Section S3), known to be upregulated in cancer cell able to evade the immune response, was used to tune parameter tum_pdlp_mut, i.e. mutational probability related to loss of immunogenicity. The relative distribution of mean gene expression level in scRNA-seq data from SMC dataset (GSE132465) (Lee *et al.*, 2020) is used, as illustrated in Supplementary Figure S10, to set the parameter as *high* in CMS1, CMS3 and CMS4 and *low* in CMS2 (see Table 1).

*Literature*: It is known that CMS3 tumor has an impaired metabolic regulation (Kather *et al.*, 2018). Thus, the parameter related to tumor consumption of nutrient *N*, i.e. tum_ncons parameter, is set at a higher rate in CMS3 with respect to the other subtype rates.

Additional information about high-throughput data, bioinformatics analyses and CMS-specific parameters setting are available in Supplementary Sections S3 and S4.

## 3 Results

### 3.1 CMS simulation: emergent properties and outcome

**Fig. 2.**
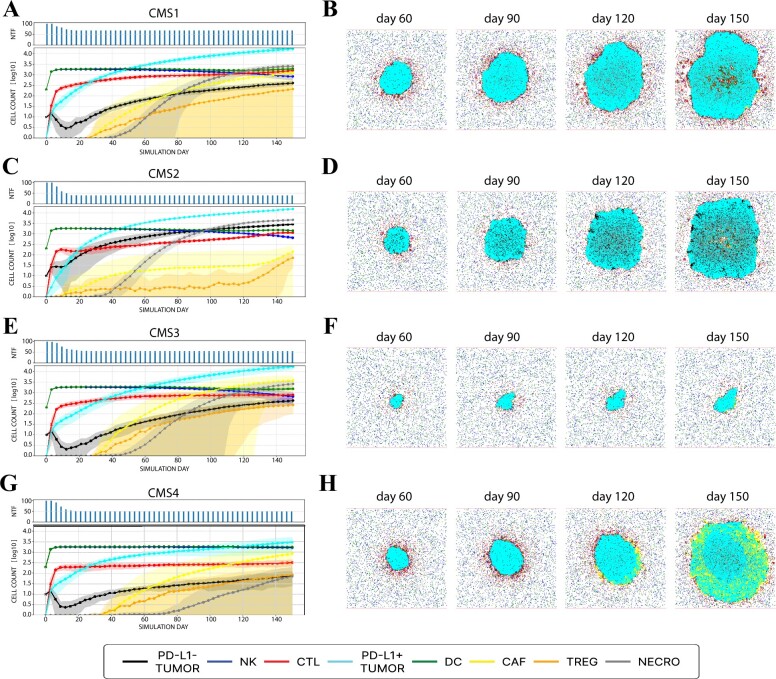
*In silico* simulation of the four CMS. Left panels (**A**, **C**, **E** and **G**) provide information on the temporal evolution of 100 simulations. In particular, in the upper subgraph, the number of not completely tumor-free (NTF) simulations in a determined instant (day), i.e. simulations having at least one cancer agent in the domain, is showed for each CMS. In the below subgraph, the time course of agent counts across NTF simulations in log10-scale is showed: continuous line represents the average count, and the shaded area represents its variability (±standard deviation). Right panels (**B**, **D**, **F** and **H**) display the spatio-temporal evolution of one simulation for each CMS. From left to right, tumor progression on Days 60, 90, 120 and 150 are represented. Legend represents color-agent association related to above representations. All graphical representations are generated using MAST

We simulated four TME scenarios representing the four molecular subtypes, i.e. CMSs (see Table 1). For each CMS, 100 *in silico* tumor-growth simulations were performed for a fixed number of iterations (around 150 days) and their execution times are shown in Supplementary Section S1 and Table S2. Our model was able to reproduce emergent properties of each subtype of the TME as shown in Figure 2.

First, we observed that all tumors are characterized by the exponential growth of cancer cells (see Fig. 2A, C, E and G and Supplementary Fig. S11), even though CMS3 growth was significantly slower, suggesting that the metabolic subtype is a slow-proliferating tumor. As shown in Supplementary Figure S12, the number of specialized CTLs in the simulated tissue in CMS1 over time was higher with respect to the other CMS, indicating a stronger immune response activation. Moreover, CTLs appeared to be infiltrated inside the tumor mass in our simulations (see Fig. 2B). These results are in line with the distinct feature of CMS1, i.e. strong immune infiltration (see Supplementary Section S2 and Fig. S5). We also observe a different number of CAFs across the different subtypes (see Supplementary Fig. S13) emerging spontaneously over time. In particular, the growth rate was significantly faster in CMS4 compared to the others. Spatially, CMS4 was characterized by high stromal component (see Fig. 2H), a well-known emergent property of this tumor subtype.

Simulation outcomes were classified into complete tumor remission, when *in silico* simulation was tumor-cell free. Histogram bar height in Figure 2A, C, E and G represents the number of simulations not completely tumor-free at different time points. In particular, CMS1 and CMS4 subgroups resulted in a high-proliferating tumor in the majority of simulations although CMS1 is characterized by higher IS cells infiltration and lower CAFs. In contrast, the canonical and metabolic subtypes, i.e. CMS2 and CMS3, were characterized by a higher tumor eradication rate. These predictions were validated using clinical outcome endpoints of progression-free interval of a 303 colorectal patient cohort from TCGA database (see Supplementary Fig. S14) and are in line with current clinical knowledge on CMS (see Supplementary Fig. S5). Additional results on simulations and clinical data analysis are available in Supplementary Section S5.

### 3.2 Effect of specific parameters on TME

To better highlight the emergent properties of the different tumor–immune dynamics and further validate the proposed model, the collective behavior of tumors under different tumor conditions was investigated for each CMS. Additional information about these simulations is provided in Supplementary Section S6.

#### 3.2.1 Effect of antigenicity

The loss of antigenicity is one of the immune escape mechanism cancer cells use to avoid elimination and increase tumorigenesis. It is expected that the higher the tumor antigenicity, the more likely the immune recognition through antigen presentation occurs, thus inducing a higher immune response (Beatty and Gladney, 2015; de Charette *et al.*, 2016). To investigate the effect of loss/acquisition of antigenicity on tumor–immune dynamics, the antigenicity rate tum_newantigen_rate was varied between 1- and 16-fold from the default value. As expected, the increasing number of antigens elicited the number of CTLs suggesting, in turn, an elicited antigen-specific immune response (see Supplementary Figs S15–S22).

#### 3.2.2 Effect of immunogenicity

Tumors can escape immune elimination by decreasing immunogenicity, i.e. upregulating inhibitory immune checkpoint genes such as PD-L1 molecules. Li *et al.* (2019) showed that PD-L1 expression in tumor cells was significantly associated with poor prognostic outcomes in CRC patients. To determine the effect of immunogenicity on tumor progression, we increased the probability of PD-L1-like genes acquisition, i.e. tum_pdlp_rate, from 1- to 16-fold from the default value (see Supplementary Figs S23–S30). In accordance with Li *et al.*, the increasing probability of expressing PD-L1-like genes resulted in higher rate of tumor-cell survival.

#### 3.2.3 Effect of nutrient consumption

Tumor survival and proliferation require the availability of nutrients in the environment. However, nutrient availability may be limited by the combined effect of nutrient demand, i.e. consumption rate of other cells, and accessibility of nutrients through vasculature (Sullivan and Vander Heiden, 2019). To observe the effect of nutrient consumption on tumor progression, we increase the tumor nutrient requirement for duplication, i.e. tum_ncons parameter, from 1- to 150-fold from the default value of non-tumor cell. As highlighted by the decreasing number of tumor cells and in accordance with Sullivan and Vander Heiden study (see Supplementary Figs S31–S38), a high nutrient demand limited tumor proliferation in a context of unchanged nutrient accessibility, i.e. unchanged modeled vasculature.

#### 3.2.4 Effect of CAFs recruitment

CAFs are known to enhance tumor proliferation and migration in many cancers, including CRC (Aizawa *et al.*, 2018). To analyze the CAFs effect on tumor progression, we decreased the probability of cell type recruitment by increasing inhib_CAF_reclut_par parameter from 1- to 30-fold from the default value. As expected, the cardinality of CAFs varied with the parameter, and tumor proliferation was promoted in response to increased CAF recruitment (see Supplementary Figs S39–S46).

## 4 Discussion

In this work, we have presented MAST, a hybrid multi-ABM to investigate how IS and tumor cells interact within TME, assessing which emergent properties arise. MAST couples a discrete and stochastic ABM, which simulates interactions and dynamics of immune and cancer cells in the TME, and a continuous, deterministic PDE-based model to simulate nutrient diffusion from vessels. Furthermore, MAST provides a wide range of graphical outputs allowing an in-depth analysis of the spatio-temporal evolution of each simulated TMEs.

It specifically models immune recognition and escape mechanisms through the acquisition of mutations, and spatio-temporal dynamics of cells in response to nutrient availability. Model parameters can be informed in a data-driven way using different sources, such as literature or high-throughput sequencing data, in order to simulate specific and unique immune–cancer microenvironment of a tumor type.

We informed MAST by coupling bulk and single-cell sequencing data of human CRC samples in order to simulate tumor progression in four heterogeneous and specific TME scenarios, i.e. CMS. To validate our model, we use different strategies. First, we assessed the ability of MAST to reproduce known emergent biological properties of the four CMS scenarios, such as a stronger immune response and stromal infiltration in CMS1 and CMS4, respectively. Then, we investigated the effect of different mutational, metabolic and stromal plasticity conditions in cancer development and tumor–immune dynamics, achieving a good agreement with current biological knowledge of tumor. Lastly, we used clinical endpoints’ real data from TCGA database to compare simulated and real tumor outcomes of corresponding CMS scenarios.

Given the data-driven approach to inform the simulator, we also tested the effect of considering different data sources on simulating real case scenarios (see Supplementary Section S7, Figs S47–S52 and Tables S4 and S5). In particular, we used TCGA or SMC dataset separately to simulate the four CRC CMSs. Although a slight change in some parameter settings was observed, different data sources provided the same distinct biological collective behavior of a tumor, while including specific and personalized features of the TME of the patient cohort.

In general, our model, like other existing ABMs, may suffer from some limitations. MAST models the TME in a ‘slice’ of tissue, considering the possibility that IS cells appear and disappear from outside (i.e. upstream and downstream) but computing nutrient diffusion in a 2D rather than 3D space. This simplification, however, allows for limiting the computational burden. One of the main factors limiting a wider application of quantitative systems modeling is its demand for rich experimental data or literature for a precise parameter estimation. Therefore, MAST is a simplification of the real mechanisms occurring in the TME. In future studies, it would be of interest to integrate other sources of information complementary to bulk and single-cell experiments to inform our model, for example imaging data or spatial transcriptomics for the spatial distribution of cells, also adding vasculature modeling. In terms of model assessment, MAST and the great majority of proposed ABMs in literature (Alfonso *et al.*, 2016; Frascoli *et al.*, 2017; Kather *et al.*, 2017; Pourhasanzade *et al.*, 2017; Wang *et al.*, 2009) are validated in a qualitatively way through the analysis of biological plausibility of simulated outcomes with known tumors characteristics, and comparison between simulated and real clinical endpoints. Further validation through image-derived data is difficult, given the limited availability of a complete and comprehensive image repository of data from individual patients or cohort of patients, and the lack of temporal data. Nevertheless, our simulations mimic the characteristic patterns of real images such as those shown by Kather *et al.* (2017).

We believe that MAST can be a useful tool to better understand tumor–immune cell dynamics that drive tumor progression in specific and unique TMEs. We think that a data-driven way to generate simulation settings could be a powerful tool in the way toward specialized and personalized studies of the TME. Ideally, the availability of patient-specific data and the ability to inform model from them can allow the modeling of patient-specific tumor–immune system dynamics.

Although some simple immunotherapy modeling has been investigated (Supplementary Table S1 and Section S8), implementation of other therapies could be incorporated in the future to investigate their combined effect on tumor progression and to test how different time-schedule affects their efficacy. Moreover, tumor mutational evolution that leads to a selective advantage related to survival and duplication fitness will be further investigated.

## Supplementary Material

vbac092_Supplementary_Data

## Data Availability

The model described in this article is implemented in Python language and it is freely available with an open-source license through GitLab (https://gitlab.com/sysbiobig/mast). MAST is also released through the Docker Registry (https://registry.gitlab.com/sysbiobig/mast/) to facilitate its distribution. The submitted software version and test data are available in Zenodo at https://dx.doi.org/10.5281/zenodo.7267745.

## References

[vbac092-B1] Aizawa T. et al (2018) Molecular characterization of cancer associated fibroblasts in colorectal cancer. Ann. Oncol., 29, ix42.

[vbac092-B2] Alfonso J.C. et al (2016) *In-silico* insights on the prognostic potential of immune cell infiltration patterns in the breast lobular epithelium. Sci. Rep., 6, 33322. 27659691 10.1038/srep33322 PMC5034260

[vbac092-B3] Baruzzo G. et al (2022) Identify, quantify and characterize cellular communication from single-cell RNA sequencing data with scSeqComm. Bioinformatics, 38, 1920–1929. 10.1093/bioinformatics/btac036 35043939

[vbac092-B4] Beatty G.L. , Gladney W.L. (2015) Immune escape mechanisms as a guide for cancer immunotherapy. Clin. Cancer Res., 21, 687–692. 25501578 10.1158/1078-0432.CCR-14-1860 PMC4334715

[vbac092-B5] Bonabeau E. (2002) Agent-based modeling: methods and techniques for simulating human systems. Proc. Natl. Acad. Sci. USA, 99, 7280–7287. 12011407 10.1073/pnas.082080899 PMC128598

[vbac092-B6] Braun D.A. et al (2016) Genomic approaches to understanding response and resistance to immunotherapy. Clin. Cancer Res., 22, 5642–5650. 27698000 10.1158/1078-0432.CCR-16-0066 PMC5135569

[vbac092-B7] Breart B. et al (2008) Two-photon imaging of intratumoral CD8+ T cell cytotoxic activity during adoptive T cell therapy in mice. J. Clin. Invest., 118, 1390–1397. 18357341 10.1172/JCI34388 PMC2268880

[vbac092-B8] Carvalho S. et al (2017) Glucose diffusion in colorectal mucosa—a comparative study between normal and cancer tissues. J. Biomed. Opt., 22, 91506. 28241323 10.1117/1.JBO.22.9.091506

[vbac092-B9] Cerignoli F. et al (2018) *In vitro* immunotherapy potency assays using real-time cell analysis. PLoS One, 13, e0193498. 29499048 10.1371/journal.pone.0193498 PMC5834184

[vbac092-B10] Clarke M.A. , Fisher J. (2020) Executable cancer models: successes and challenges. Nat. Rev. Cancer, 20, 343–354. 32341552 10.1038/s41568-020-0258-x

[vbac092-B11] Dagogo-Jack I. , Shaw A.T. (2018) Tumour heterogeneity and resistance to cancer therapies. Nat. Rev. Clin. Oncol., 15, 81–94. 29115304 10.1038/nrclinonc.2017.166

[vbac092-B12] de Charette M. et al (2016) Turning tumour cells into antigen presenting cells: the next step to improve cancer immunotherapy? Eur. J. Cancer, 68, 134–147. 27755997 10.1016/j.ejca.2016.09.010

[vbac092-B13] Finotello F. et al (2019) Molecular and pharmacological modulators of the tumor immune contexture revealed by deconvolution of RNA-seq data. Genome Med., 11, 1–20. 31126321 10.1186/s13073-019-0638-6 PMC6534875

[vbac092-B14] Frascoli F. et al (2017) A model of the effects of cancer cell motility and cellular adhesion properties on tumour-immune dynamics. Math. Med. Biol., 34, 215–240. 27094601 10.1093/imammb/dqw004

[vbac092-B15] Fucikova J. et al (2020) Detection of immunogenic cell death and its relevance for cancer therapy. Cell Death Dis., 11, 1–13. 33243969 10.1038/s41419-020-03221-2 PMC7691519

[vbac092-B16] Gong C. et al (2017) A computational multiscale agent-based model for simulating spatio-temporal tumour immune response to PD1 and PDL1 inhibition. J. R. Soc. Interface, 14, 20170320. 28931635 10.1098/rsif.2017.0320 PMC5636269

[vbac092-B17] Greten F.R. , Grivennikov S.I. (2019) Inflammation and cancer: triggers, mechanisms, and consequences. Immunity, 51, 27–41. 31315034 10.1016/j.immuni.2019.06.025 PMC6831096

[vbac092-B18] Guinney J. et al (2015) The consensus molecular subtypes of colorectal cancer. Nat. Med., 21, 1350–1356. 26457759 10.1038/nm.3967 PMC4636487

[vbac092-B19] Hanahan D. , Coussens L.M. (2012) Accessories to the crime: functions of cells recruited to the tumor microenvironment. Cancer Cell, 21, 309–322. 22439926 10.1016/j.ccr.2012.02.022

[vbac092-B20] Hawse W.F. , Morel P.A. (2014) An immunology primer for computational modelers. J. Pharmacokinet. Pharmacodyn., 41, 389–399. 25238901 10.1007/s10928-014-9384-y PMC4850904

[vbac092-B21] Huang C. et al (2019) Immune checkpoint molecules. Possible future therapeutic implications in autoimmune diseases. J. Autoimmun., 104, 102333. 31564474 10.1016/j.jaut.2019.102333

[vbac092-B22] Huang A.Y. , Lee E.A. (2022) Identification of somatic mutations from bulk and single-cell sequencing data. Front. Aging, 2, 800380. 35822012 10.3389/fragi.2021.800380 PMC9261417

[vbac092-B23] Joshi R.S. et al (2021) The role of cancer-associated fibroblasts in tumor progression. Cancers (Basel), 13, 1399–1327. 33808627 10.3390/cancers13061399 PMC8003545

[vbac092-B24] Kather J.N. et al (2017) *In silico* modeling of immunotherapy and stroma-targeting therapies in human colorectal cancer. Cancer Res., 77, 6442–6452. 28923860 10.1158/0008-5472.CAN-17-2006

[vbac092-B25] Kather J.N. et al (2018) Genomics and emerging biomarkers for immunotherapy of colorectal cancer. Semin. Cancer Biol., 52, 189–197. 29501787 10.1016/j.semcancer.2018.02.010

[vbac092-B26] Lee H.O. et al (2020) Lineage-dependent gene expression programs influence the immune landscape of colorectal cancer. Nat. Genet., 52, 594–603. 32451460 10.1038/s41588-020-0636-z

[vbac092-B27] Li Y. et al (2019) The prognostic and clinicopathological roles of PD-L1 expression in colorectal cancer: a systematic review and meta-analysis. Front. Pharmacol., 10, 139. 30873025 10.3389/fphar.2019.00139 PMC6403169

[vbac092-B28] Martínez-Lostao L. et al (2015) How do cytotoxic lymphocytes kill cancer cells? Clin. Cancer Res., 21, 5047–5056. 26567364 10.1158/1078-0432.CCR-15-0685

[vbac092-B29] Norton K.A. et al (2018) Modeling triple-negative breast cancer heterogeneity: effects of stromal macrophages, fibroblasts and tumor vasculature. J. Theor. Biol., 452, 56–68. 29750999 10.1016/j.jtbi.2018.05.003 PMC6127870

[vbac092-B30] Norton K.A. et al (2019) Multiscale agent-based and hybrid modeling of the tumor immune microenvironment. Processes (Basel), 7, 37. 30701168 10.3390/pr7010037 PMC6349239

[vbac092-B31] Peskov K. et al (2019) Quantitative mechanistic modeling in support of pharmacological therapeutics development in immuno-oncology. Front. Immunol., 10, 924. 31134058 10.3389/fimmu.2019.00924 PMC6524731

[vbac092-B32] Plava J. et al (2019) Recent advances in understanding tumor stroma-mediated chemoresistance in breast cancer. Mol. Cancer, 18, 1–10. 30927930 10.1186/s12943-019-0960-z PMC6441200

[vbac092-B33] Pourhasanzade F. et al (2017) An agent-based model of avascular tumor growth: Immune response tendency to prevent cancer development. *Simulation*, 93, 641–657. doi:10.1177/0037549717699072.

[vbac092-B34] Racle J. et al (2017) Simultaneous enumeration of cancer and immune cell types from bulk tumor gene expression data. Elife, 6, e26476. 29130882 10.7554/eLife.26476 PMC5718706

[vbac092-B35] Sauter B. et al (2000) Consequences of cell death: exposure to necrotic tumor cells, but not primary tissue cells or apoptotic cells, induces the maturation of immunostimulatory dendritic cells. J. Exp. Med., 191, 423–434. 10662788 10.1084/jem.191.3.423 PMC2195816

[vbac092-B36] Stratton M.R. et al (2009) The cancer genome. Nature, 458, 719–724. 19360079 10.1038/nature07943 PMC2821689

[vbac092-B37] Sturm G. et al (2019) Comprehensive evaluation of transcriptome-based cell-type quantification methods for immuno-oncology. Bioinformatics, 35, i436–i445. 31510660 10.1093/bioinformatics/btz363 PMC6612828

[vbac092-B38] Sullivan M.R. , Vander Heiden M.G. (2019) Determinants of nutrient limitation in cancer. Crit. Rev. Biochem. Mol. Biol., 54, 193–207. 31162937 10.1080/10409238.2019.1611733 PMC6715536

[vbac092-B39] Tang S. et al (2020) Mechanisms of immune escape in the cancer immune cycle. Int. Immunopharmacol., 86, 106700. 32590316 10.1016/j.intimp.2020.106700

[vbac092-B40] Thomas R.M. et al (2016) Concepts in cancer modeling: a brief history. Cancer Res., 76, 5921–5925. 27694601 10.1158/0008-5472.CAN-16-1293 PMC5117812

[vbac092-B41] Thorsson V. et al; Cancer Genome Atlas Research Network. (2018) The immune landscape of cancer. Immunity, 48, 812–830.e14. 29628290 10.1016/j.immuni.2018.03.023 PMC5982584

[vbac092-B42] Vesperini D. et al (2021) Characterization of immune cell migration using microfabrication. Biophys. Rev., 13, 185–202. 34290841 10.1007/s12551-021-00787-9 PMC8285443

[vbac092-B43] Vicens A. , Posada D. (2018) Selective pressures on human cancer genes along the evolution of mammals. Genes (Basel), 9, 582. 30487452 10.3390/genes9120582 PMC6316132

[vbac092-B44] Wang Z. et al (2009) Cross-scale, cross-pathway evaluation using an agent-based non-small cell lung cancer model. Bioinformatics, 25, 2389–2396. 19578172 10.1093/bioinformatics/btp416 PMC2735669

[vbac092-B45] Wiedemann A. et al (2006) Cytotoxic T lymphocytes kill multiple targets simultaneously via spatiotemporal uncoupling of lytic and stimulatory synapses. Proc. Natl. Acad. Sci. USA, 103, 10985–10990. 16832064 10.1073/pnas.0600651103 PMC1544161

