# Supplementary material for: MAST: a hybrid Multi-Agent Spatio-Temporal model of tumor microenvironment informed using a data-driven approach
Source: Bioinform Adv. 2022 Dec 5;2(1):vbac092. doi: 10.1093/bioadv/vbac092 (PMC9744439; doi:10.1093/bioadv/vbac092)
Supplement: vbac092_Supplementary_Data [file vbac092_supplementary_data.pdf]

MAST: a hybrid Multi-Agent Spatio-Temporal model of tumor microenvironment informed using a data-driven approach

Giulia Cesaro[1,†] [giulia.cesaro.1@studenti.unipd.it]
Mikele Milia[1,†] [mikele.milia@unipd.it]
Giacomo Baruzzo[1] [giacomo.baruzzo@unipd.it]
Giovanni Finco[1] [gfinco@phys.ethz.ch]
Francesco Morandini[1] [fmorandi@ur.rochester.edu]
Alessio Lazzarini[1] [alessio.lazzarini@studenti.unipd.it]
Piergiorgio Alotto[2] [piergiorgio.alotto@unipd.it]
Noel Filipe da Cunha Carvalho de Miranda[3] [N.F.de_Miranda@lumc.nl]
Zlatko Trajanoski[4] [zlatko.trajanoski@i-med.ac.at]
Francesca Finotello[4,5,6] [francesca.finotello@i-med.ac.at]
Barbara Di Camillo[1,7,*] [barbara.dicamillo@unipd.it]

[1]Department of Information Engineering, University of Padova, Padova, Italy,
[2]Department of Industrial Engineering, University of Padova, Padova, Italy,
[3]Department of Pathology, Leiden University Medical Center, Leiden, The Netherlands,
[4]Biocenter, Institute of Bioinformatics, Medical University of Innsbruck, Innsbruck 6020, Austria,
[5]Institute of Molecular Biology, University Innsbruck, Innsbruck 6020, Austria,
[6]Digital Science Center (DiSC), University Innsbruck, Innsbruck 6020, Austria,
[7]Department of Comparative Biomedicine and Food Science, University of Padova, Padova, Italy.

*To whom correspondence should be addressed. † These authors contributed equally to this work.

Corresponding author: Barbara Di Camillo;
Email: barbara.dicamillo@unipd.it;
Address: via Gradenigo 6B, 35131, Padova, Italy;
Phone number: +39 049 8277671;

Supplementary Materials

Section S1 - Package and implementation details

S1.1 Usage

MAST is freely available Python software that can be used as a simulator of microenvironments or a visualizer of their results. To be used, MAST requires both mandatory and optional parameters. For more details, please refer to its usage guide (https://gitlab.com/sysbiobig/mast/).

S1.2 Docker & Singularity

MAST is released through the Docker Registry (https://registry.gitlab.com/sysbiobig/mast/) to facilitate its distribution and use by clinicians and researchers. The Docker image is built on Ubuntu 18.04 LTS (Bionic Beaver) and within it in addition to MAST there is the Fortran module developed to solve steady state PDEs. In order to pull the Docker image from the Registry, a user can use the following line of code:

```
$  docker pull registry.gitlab.com/sysbiobig/mast
```

After the container has been successfully pulled, the user can interact with MAST using the following line of code:

```
$  docker run -v $(pwd)/output:/home/mast/output \
              -v $(pwd)/plot:/home/mast/plot \
             -v $(pwd)/data:/home/mast/data \
              -u $(id -u):$(id -g) \
              -ti registry.gitlab.com/sysbiobig/mast \
              src/main.py [...]
```

In case MAST needs to be run on a High-Performance Computing (HPC) cluster, the previously built Docker container can be converted to a Singularity one and run via the following lines of code:

```
$  singularity      build      <container>      docker-daemon://regis-
   try.gitlab.com/sysbiobig/mast:latest

$  singularity exec --bind $(pwd)/output:/home/mast/output \
                    --bind $(pwd)/plot:/home/mast/plot \
                    --bind $(pwd)/data:/home/mast/data \
                    --pwd /home/mast \
                    singularity/mast.sif \
                    python3 src/main.py [...]
```

In both circumstances, [...] represents the list of mandatory and optional parameters that MAST expects to receive from the command line, as explained in the Section S1.1.

S1.3 Implementation details

MAST is implemented as a Python package. The Agent Based Model (ABM) module is written in Python while the Partial Differential Equation (PDE) module is written in Fortran. Their interaction occurs through a Python wrapper. To simulate a TME, a mandatory input set of parameters is required. Any parameter can be specified by the user, estimated from real data, or taken from our defaults.

For reproducibility purposes, a simulation is always initialized with a unique seed (e.g., its index). Since simulations are independent, MAST allow parallel execution through multi-processing. Given the complexity and multidimensionality of outputs (see Section S1.5) generated by the model, users can generate interactively or via command line several static and dynamic graphical representations (see Section S1.6), along with tabular outputs, to observe collective behavior of simulated TME both in space and time.

S1.4 Simulation pseudocode

MAST performs several operations at each time step. Specifically, the following pseudo code schematizes the operations that occur.

```
For each time-step t:
  - Update the concentration of nutrients (PDE model)
  - For each agent in the grid:
    - Sample the action
    - Perform the action
    - Update adjuvanticity signal
```

Upon the setting of the parameters of the simulation and the initialization of the agents of the first temporal instant, a simulation step begins with the diffusion of the nutrients useful to the duplication and survival of the agents in the tissue. The frequency with which PDEs diffuse substances from the vessels to the tissue can be specified by the user in relation to the available computational capacity. After nutrient diffusion, each agent in the grid samples and implements an action. For this reason, at the end of each simulation step signaling molecules are updated, in order to notify the areas of the tissue where a fight between immune system and tumor cells is occurring.

S1.5 File management

S1.5.1 Simulation input

MAST allows the user to define in input a structure of folders useful to group the micro-environments of interest during the runs. In detail, inside the folder "data" it is possible to define a folder that is interpreted as environment, inside every folder environment it is possible to define an arbitrary number of sub-folders called systems useful to separate logically the micro-environments that must be simulated. An arbitrary number of files (.dat) can be defined within a microenvironment folder. MAST accepts only two types of files as input, those related to the general setup (.sim) and those related to the specific configuration of tumor microenvironments (.dat). Both these files contain encoded information in format:

```
parameter:value
```

MAST contains only one setup (.sim) file and it is used to set all the default parameters necessary to execute a basic simulation. In order to simulate custom microenvironments, it is necessary to produce a (.dat) file for each one containing a particular subset of parameters that must be overwritten in the setup (.sim) file. In order to avoid inconsistencies between microenvironments, configuration parameters overwrite default parameters locally. In fact, after the end of all the simulations of microenvironment (k), the overwritten values are reset to their defaults since a priori it is not possible to know which subset of parameters has been modified. The complete list of parameters used by MAST is showed in Table S1.

Parameter	MAST Parameter	Meaning	Default	Range	Source
W	width	Number of rows of the grid	200	> 1	-
H	height	Number of columns of the grid	200	> 1	-
num_sim	num_sim	Number of simulations per system	100	> 1	-
num_cycles	num_cycles	Number of simulation cycles (one cycle represents one model time-step)	600	> 1	-
num_hours	num_hours	Duration of a simulation time step (number of hours corresponding to a cy-cle)	6	> 0	(Breart et al., 2008)
day_save	day_save	Number of days before saving tabular information (agent position and anti-gens)	3	1 to num_cycles / 24 / num_hours	-
gif	gif_generate	Logical value that enables GIF genera-tion	1	0 or 1	-
gif_dpi	gif_dpi	Number of DPI to be used during GIF generation	150	150 to 300	-
gif_fps	gif_fps	Number of FPS to be used during GIF generation	4	1 to 12	-
pde	pde	Logical value that enables PDE diffu-sion	1	0 or 1	-
pde_update	pde_update	Number of cycles occurring between each PDE update	12	1 to *num_cycles*	-
pde_diffusion	pde_diffusion	Nutrient diffusion coefficient	0.00 1	> 0	(Carvalho et al., 2017)
k_i *(nutrient M)*	mcons	Nutrient M consumption rate of a non-tumor agent (e.g., oxygen)	0.00 2	0 to 1	-
k_i *(nutrient N)*	ncons	Nutrient N consumption rate of a non-tumor agent (e.g., glucose)	0.00 2	0 to 1	-
k_t *(nutrient M)*	tum_mcons	Nutrient M consumption rate of a tumor agent (e.g., oxygen)	0.02	0 to 1	(Yalcin et al., 2009)
k_t *(nutrient N)*	tum_ncons	Nutrient N consumption rate of a tumor agent (e.g., glucose)	0.02	0 to 1	(Yalcin et al., 2009)
tumor_radius	tum_radius	Radius of monoclonal tumor cluster at time zero	1	> 1	-
tumor_row_position	tum_x_coord	Initial tumor cluster row position	100	0 to *W-1*	-

tumor_column_position	tum_y_coord	Initial tumor cluster column position	100	0 to *H-1*	-
tumor_duplication_delay	tum_div	Number of cycles after which each newborn cell can duplicate	2	> 1	(Kather et al., 2017)
θ_{dupl}	tum_dupl_par	Parameter related to mutational status of tumor cell, so that there might be cell duplicating at higher rate than others at the same nutrient concentrations, depending on duplication cell fitness (see Equation (1))	1.2	> 0	-
θ_{dupl_stroma}	tum_duplstroma_par	CAF rate of promoting duplication of tumor cells (see Equation (1))	0.5	> 0	-
θ_{necr}	tum_necr_par	Parameter related to mutational status of tumor cell, so that cells might survive easily than others in an environment poor of nutrient M, depending on survival cell fitness (see Equation (2))	0.2	> 0	-
necrotic_life_cycles	max_necr_cycles	Necrotic cell's maximum number of life cycles before disappearing	20	> 0	-
θ_{move}	tum_mov_par	Parameter related to tumor cell mobility (see Equation (3))	4	> 0	-
caf_impermeability	caf_imperm	CAF impermeability	1	0 to 1	-
tum_mut_rate	tum_mut_rate	Tumor mutational rate	0.5	0 to 1	-
tum_duplchange_rate	tum_duplchange_rate	If a mutation occurs, this is the probability to induce a change in θ_{dupl} parameter	0	0 to 1	-
tum_newantigen_rate	tum_newantigen_rate	If a mutation occurs, this is the probability to induce a new antigen presentation	0.01	0 to 1	data
tum_adjchange_rate	tum_adjchange_rate	If a mutation occurs, this is the probability to release signaling molecules that locally repel immune cells	0.00 5	0 to 1	data

tum_necrchange_rat e	tum_necrchange_rate	If a mutation occurs, this is the probability to induce a change in θ_{necr} parameter	0	0 to 1	-
tum_pdlp_mut_rate	tum_pdlp_mut_rate	If a mutation occurs, this is the probability to induce a PD-L1+ like mutation	0.02	0 to 1	data
tum_pdlm_mut_rate	tum_pdlm_mut_rate	If a mutation occurs, this is the probability to lose a PD-L1+ like mutation	0.00 5	0 to 1	-
immune_movement_radius	maxmov	Immune system cell maximum movement per simulation cycle	10	0 to min(W, H)	-
immune_death_probability	p_die	Probability of immune cells to disappear from the domain	0.1	0 to 1	(Kather et al., 2017)
minimum_adjuvanticity_value	adj_min	Adjuvanticity signal value when Treg/CAF are in the neighborhood or when a cancer cell acquires a mutation that repel IS locally and is killed	1	> 0	-
maximum_adjuvanticity_value	adj_max	Adjuvanticity signal value when a cancer cell dies by lack of nutrients or by IS attack	4	> *adj_min*	-
innate_recruitment_probability	innate_perc	Probability of recruiting DC or NK when there is no adjuvanticity signal in the neighborhood	0.00 5	0 to 1	-
innate_killing_probability	innate_kill	Probability of NKs to kill a tumor cell	0.1	0 to 1	(Cerignoli et al., 2018)
dc_reclut_proportion	dc_reclut_par	Multiplicative coefficient for recruitment probability of DC in the environment applied to $p_{recruit}$ in Equation (4)	0.2	0 to 1	-
nk_reclut_proportion	nk_reclut_par	Multiplicative coefficient for recruitment probability of NK in the environment applied to $p_{recruit}$ in Equation (4)	0.1	0 to 1	-
ctl_reclut_proportion	ctl_reclut_par	Multiplicative coefficient for recruitment probability of CTL in the environment applied to $p_{recruit}$ in Equation (4)	0.5	0 to 1	-
inhib_TREG_recruit_p ar	inhib_treg_reclut_par	Parameter related to Equation (5) to obtain the probability of recruiting Treg in the environment	0.2	> 0	data

in-hib_CAF_recruit_par	inhib_caf_reclut_par	Parameter related to Equation (6) to obtain the probability of recruiting CAF in the environment	0.1	> 0	data
TREG_reclut_par	treg_reclut_par	Probability to recruit a Treg once a CTL is exhausted (after *ctl_maximum_attack*)	0.5	0 to 1	-
ctl_maximum_attack	ctl_duration	Number of CTL disposable attacks before getting exhausted	6	> 0	(Christophe et al., 2015)
ctl_killing_probability	ctl_kill	Probability of CTLs to kill a tumor cell	0.3	0 to 1	(Wiedemann et al., 2006)
ctl_killing_pdlp_probability	ctl_kill_pdlp	Probability of CTLs to kill a PD-L1+ like tumor cell	0.15	0 to 1	(Peng et al., 2020)
ctl_penalty	ctl_penalty	Logical value indicating whether a CTL can reduce its disposable attacks when an attack fails	0	0 or 1	-
inject_cure	inject_cure	Comma-separated list of days in which the ICI treatment starts	0	[1 to num_cycles / 24 / num_hours, …]	(Robert, 2020)
drug_duration	drug_duration	Comma-separated list of treatment durations (must be consistent with the inject-cure parameter)	0	[> 0, …, >0]	-

Table S1 – MAST parameters list. *In this table, the list of all the parameters used within MAST alongside their default value, possible ranges, and source of information for their setting (data = data-driven strategy proposed by MAST, see Section S3-4) is showed.*

S1.5.2 Tabular outputs

For each input environment considered MAST automatically generates a corresponding output folder. Each environment folder can be composed of several subfolders equal to the number of systems that will be analyzed. Inside each subfolder, during each simulation, MAST writes two files (.csv), one related to the position of the agents and one related to the antigens acquired from specific agents. Both files are encoded to avoid the generation of large output files. The frequency with which these files are updated depends on the DAY_SAVE parameter, at each saving time the status of the simulation is appended to both files. Specifically, for the agent position file each agent coordinates are linearized. To linearize agent coordinates, each cell position (i, j) in the tissue is converted in row-major order as $(i \times \text{WIDTH} + j)$ and, apart from the first agent found, all the other agents will encode its position as the difference from their index and the index of the previous agent of the same type. In this way, the final file, for example, looks like the following list:

```
tumor,16106,596,14,187,18,408,192,998,10,763,793,...
necro,18108,2801,3380
n_killer,208,61,27,29,2,7,47,26,115,11,68,137,15,...
ctl,1943,405,3564,675,1163,1365,21,993,171,18,25,...
pdlp,14313,796,2,195,4,2,1,195,4,1,1,192,1,1,1,2,...
dc,223,31,25,5,7,41,85,16,11,22,38,21,4,52,18,62,...
stroma,14713,201,394,3,198,1,201,207,4,1,1,6,2,8,...
```

On the other hand, the antigens file for each agent that can expose antigens (i.e., tumor, PD-L1, and CTL) generates a row per agent having the same cardinality presented in the positions file. Since, apart from CTLs, which are specific antigens, the tumor can present several antigens on the membrane, inside the file, the presented antigens are reported separated by the % character. Moreover, whenever the next agent presents the same antigens, a blank character is inserted, as showed for example in the following list:

```
tumor,noadj%,,,,noadj%g%x%n%,x%p%l%,noadj%x%h%b%,...
ctl,b%,a%,,noadj%,j%,,,g%,k%,y%,,b%,a%,b%,,,,a%,,...
pdlp,noadj%,,,,o%,,noadj%w%h%,y%,,,,,,noadj%m%y%,...
```

S1.6 Plotter

MAST provides the user with a Plotter consisting of a wide range of graphs and animations that can be generated interactively or via command line to observe the progression of simulated tumor micro-environments.

If the user is interested in interacting from the command line with the Plotter, please refer to the user guide for more details on the parameters that MAST expects to receive. If, on the other hand, the user is interested in interacting with the Plotter, a menu will appear that will guide him in the choice of environments and systems, finally providing the possibility of choosing which type of information graph to produce. The types of graphs useful for observing temporal (static) and spatio-temporal (dy-namic) development that can be produced using the MAST tracer are analyzed below.

Temporal evolution: multi-agent evolution within multiple systems

This temporal static graph allows comparing multiple systems, each one with multiple agents at the same time. In detail, each panel consists of a graph composed of two subplots that evolve during days. While the upper subplot (bar plot) keeps track of the number of not tumor-cell free (NTF) simulations in each day, the lower subplot presents the temporal evolution of each agent, representing its average cardinality and its standard deviation, both calculated on the day across NTF simulations.

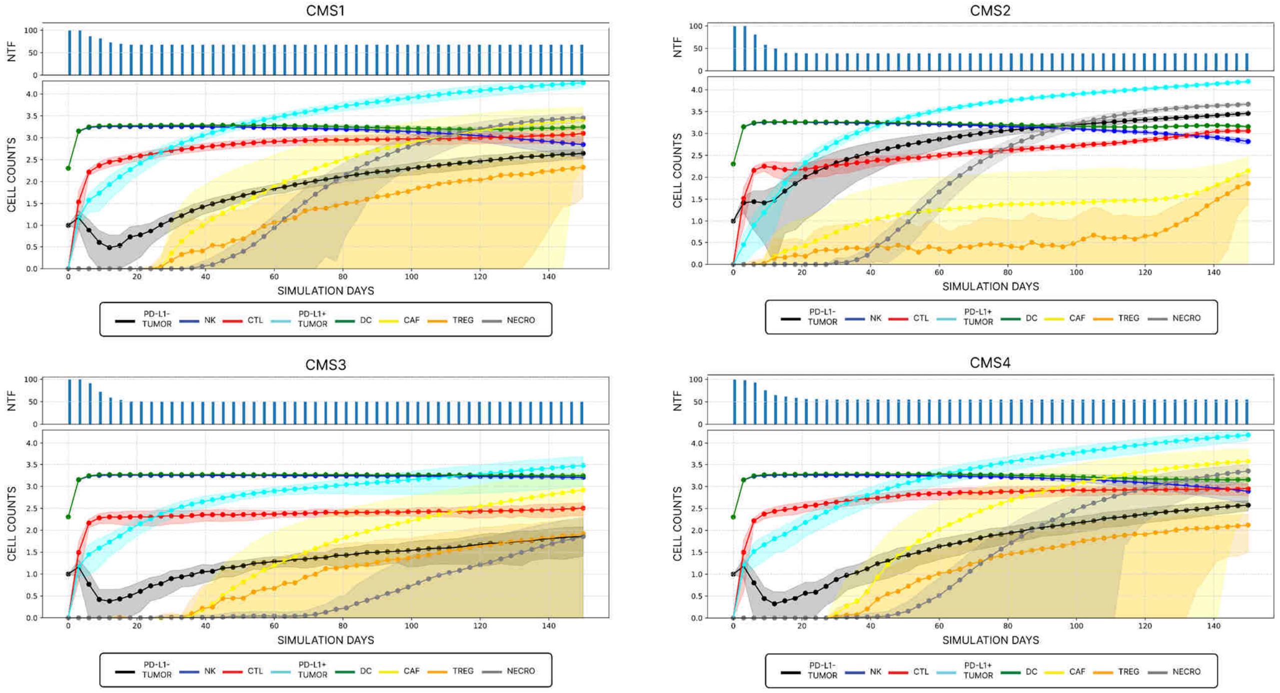

Figure S1 – Example of a multi-agent multi-system plot. *Each panel represents the simulation results of a specific system. In the upper subgraph of each panel, the number of not completely tumor-free (NTF) simulations in a determined instant (day), i.e., simulations having at least one cancer agent in the domain, is showed for each system (e.g., CMS1, CMS2, CMS3, and CMS4). In the below subgraph of each panel, the continuous line represents the average number of cells, and the shaded area repre-sents its variability (± standard deviation), computed across all NTF simulations. Legend below rep-resents agent-color association. This graphical representation is generated using MAST.*

Temporal evolution: single-agent evolution within multiple systems

This temporal static graph allows comparing the time course of cell counts of a single agent across multiple systems. In detail, this graph is composed of two subplots that evolve during days. While the upper subplot (bar plot) keeps track of the number of not tumor-cell free (NTF) simulations in each day, the lower subplot presents the temporal evolution of each agent, representing its average cardinality and its standard deviation, both calculated on the day across NTF simulations.

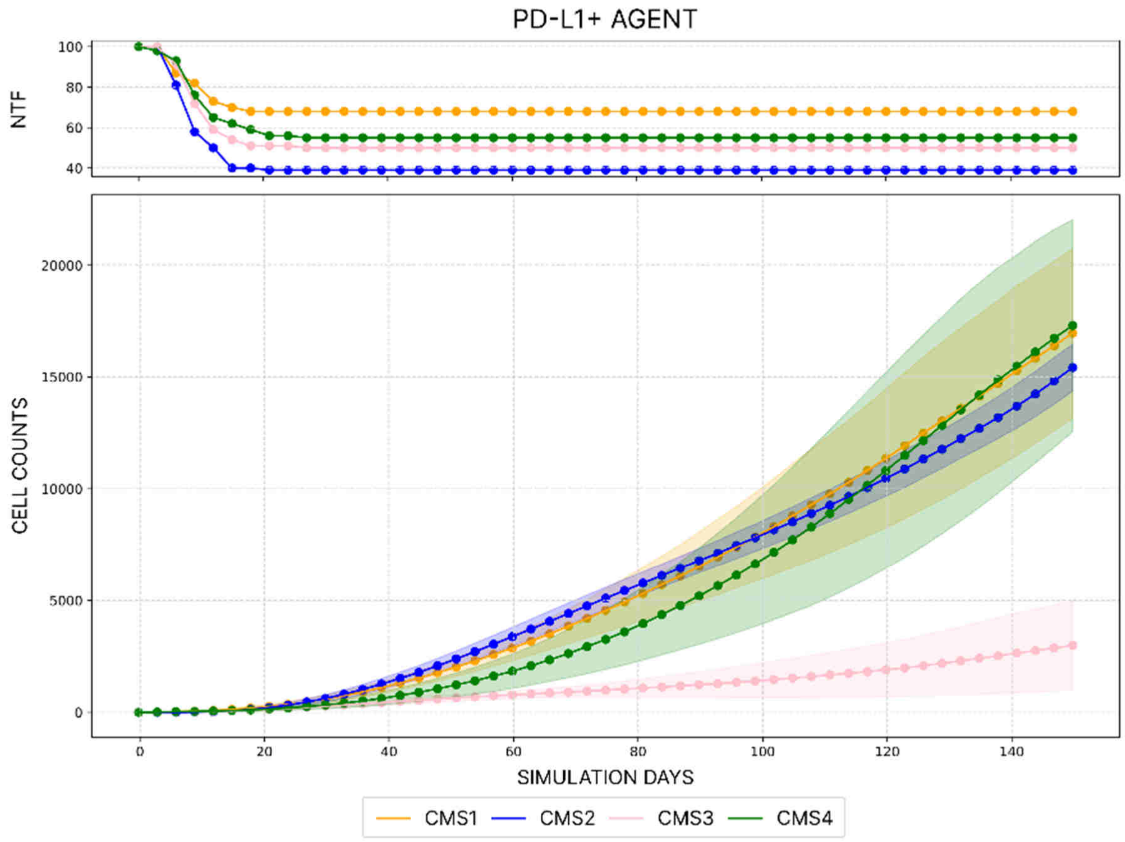

Figure S2 – Example of a single-agent multi-system plot. *In the upper subgraph, the number of not completely tumor-free (NTF) simulations in a determined instant (day), i.e., simulations having at least one cancer agent in the domain, is showed for each system (e.g., CMS1, CMS2, CMS3, and CMS4). In the below subgraph, the continuous line represents the average number of a given agent (e.g., PD-L1+ agent) and the shaded area represents its variability (± standard deviation), computed across all NTF simulations of a given system. Legend below represents system-color association. This graphical representation is generated using MAST.*

Temporal evolution: antigens evolution within a single simulation

This temporal static graph allows to observe how the immune system of a specific microenvironment reacts to antigens exposed by cancer agents (with and without PD-L1-like mutation), through the maturation of cytotoxic T lymphocytes by the lymph nodes. In detail, this graph is composed of three subplots, one for each agent that can borrow antigens (i.e., CTL, PD-L1- tumor, PD-L1+ tumor). Each agent subplot shows which antigens are acquired/lost during days.

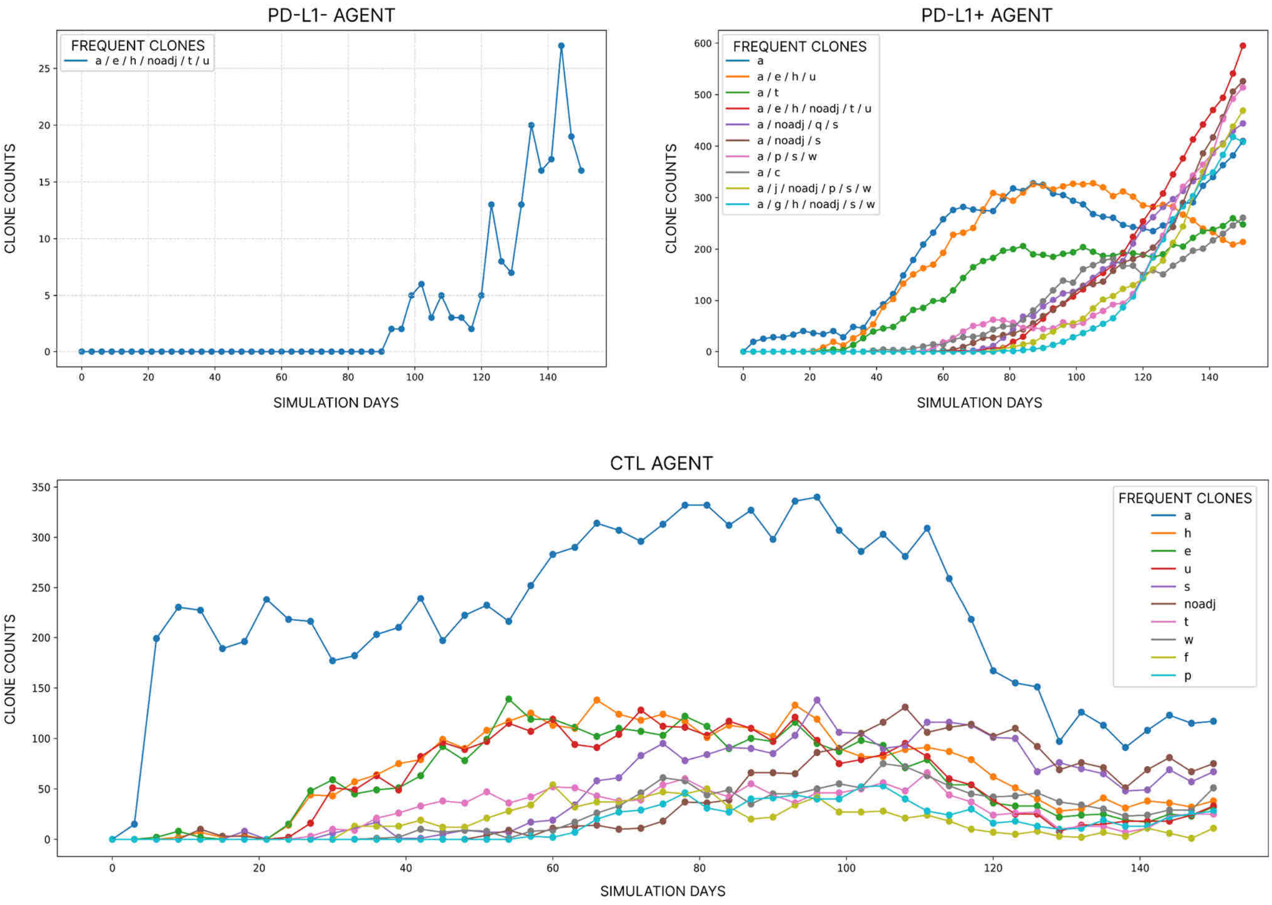

Figure S3 – Example of an antigen evolution plot. *Each subgraph represents the top 10 antigens cardinality across a selected simulation of interest. Respectively, subgraphs show antigens of PD-L1- (upper-left), PD-L1+ (upper-right) and CTL (lower) cell.*

Spatio-temporal evolution: single simulation

This spatio-temporal animation is simulation-specific and can be used both in synchronous or asynchronous way.

To generate an asynchronous GIF, the user must go through the menu which requires to decide which system simulation among the possible ones should be animated. Then, the simulation output files are decoded and concatenated.

To generate a synchronous GIF the GIF parameter must be set to 1. In this way MAST saves and collects heterogeneous information such as the position of the agents, the diffusion state of nutrient N and M (representing molecules such as oxygen and glucose), the adjuvanticity signal map in every simulation cycle, and set up a four-element grid in order to visualize the overall progression. In detail, the left side displays the tissue with the respective agent positions, while the right side displays the spatial distribution of nutrients and of adjuvanticity signal, stacked on top of each other. This visualization has enormous advantages, among which the possibility to trace many aspects of the simulator at the same time.

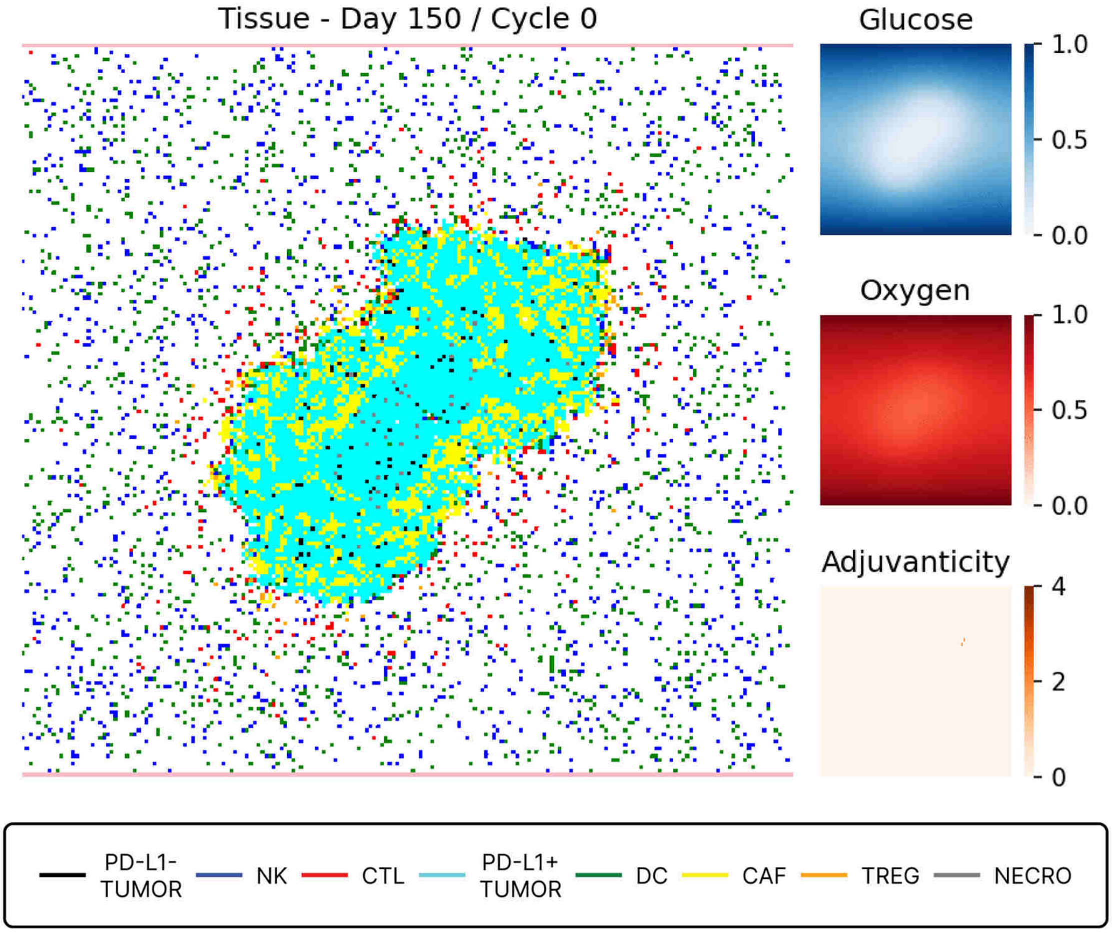

Figure S4 – Example of synchronous spatio-temporal evolution GIF for a given simulation step. *Left panel represents the spatial distribution of agents within MAST environment. Distribution in the domain of nutrient N (e.g., glucose), nutrient M (e.g., oxygen), and adjuvanticity signal are showed respectively from top to bottom right panels. The legend below represents agent-color association.*

S1.7 Computational burden

The computational burden of MAST was assessed by measuring the average simulations running time. Total computational times were normalized for the number of non tumor-free (NTF) simulations at the end of the run, in order to obtain a normalized average time of a single simulation. This choice was made because simulations inside which the immune system prevailed over the tumor had negligible durations.

We performed different test varying the simulation grid (tissue) size (i.e., 200, 300, 400). Tests were aimed at measuring the computational burden of the tool, therefore its accuracy in predicting TMEs emergent properties was not taken into consideration (see Table S2). MAST runtime was measured through the *datetime* Python module and tests were performed on a computing cluster node (Intel Xeon Gold 5220 CPU @ 2.20/3.90GHz).

In Table S2, the sequential computational times are reported and can be reduced exploiting the *multiprocessing* Python module.

As can be seen, the simulations presented in the main manuscript use a grid size of 200x200 as they account for a relevant portion of the tissue with acceptable computation time.

	**Grid size**	**NTF**	**Average simulation runtime**	**Total simulation runtime**
CMS1	200x200	66/100	~ 2 h	~ 5 days
CMS2	200x200	40/100	~ 1 h 40 min	~ 2 days
CMS3	200x200	11/100	~ 2 h 20 min	~ 1 days
CMS4	200x200	21/100	~ 2 h 40 min	~ 2 days
CMS1	300x300	68/100	~ 13 h 25 min	~ 38 days
CMS2	300x300	46/100	~ 12 h 25 min	~ 24 days
CMS3	300x300	24/100	~ 12 h 30 min	~ 12 days
CMS4	300x300	50/100	~ 17 h 30 min	~ 36 days
CMS1	400x400	64/100	~ 31 h 10 min	~ 83 days
CMS2	400x400	37/100	~ 32 h 40 min	~ 50 days
CMS3	400x400	15/100	~ 30 h 20 min	~ 19 days
CMS4	400x400	46/100	~ 34 h 30 min	~ 66 days

Table S2 – MAST runtime using serial and parallel modes. *Tests were performed on a computing cluster node (Intel Xeon Gold 5220 CPU @ 2.20/3.90GHz).*

Section S2 – Biological characterization of Consensus Molecular Subtypes (CMSs)

Human colorectal cancer tissues can be classified based on gene expression profile into four consensus molecular subtypes (CMSs) with distinctive biological features (Kather et al., 2018):

- CMS1, named microsatellite instability immune subtype, is characterized by hypermutated and microsatellite unstable tumors and strong immune infiltration;
- CMS2, named canonical subtype, is immune-neglected;
- CMS3, named metabolic subtype, shows a metabolic dysregulation;
- CMS4, named mesenchymal subtype, has an abundant stroma invasion, which induces an immunosuppressive microenvironment, and is characterized by poor prognosis.

Nowadays, CMS classification is the mostly used classification system and it represents a basis for subtype-based target therapy (Guinney et al., 2015). Thus, there is the need of a better understanding of biological processes implicated in each subtype and their clinical relevance.

The following figure (Figure S5) summarizes current biological knowledge on CMS groups and their significant biological differences (Fessler and Medema, 2016; Picard et al., 2020).

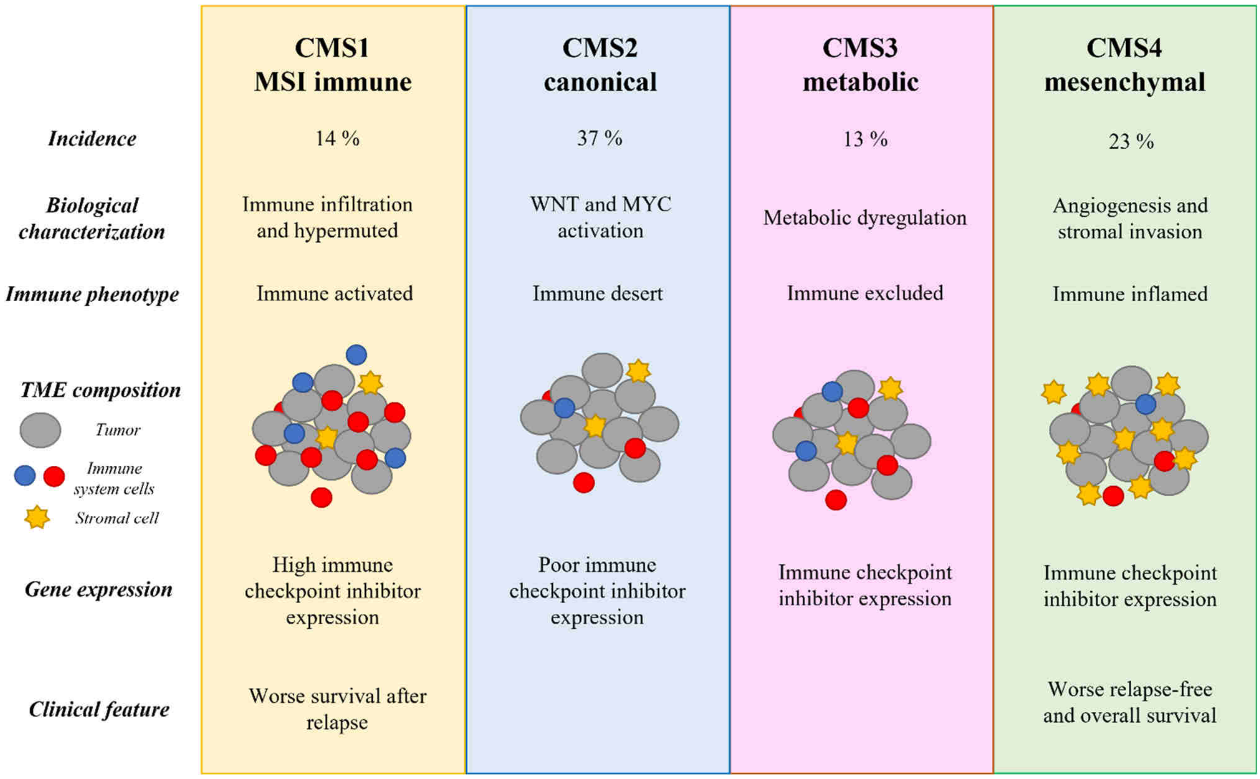

Figure S5 – Biological differences and emergent properties of each Consensus Molecular Subtype. *Distinct genetic modifications, immune phenotype, biological processes, and clinical outcomes are implicated in each subtype.*

Section S3 – Source of information and bioinformatics analyses for index estimation

To model unique characteristics of a TME, it is possible to specify model parameters using different sources of information, such as literature or real data from high-throughput experiments.
Literature source provides static information about mean behavior of TME characteristics in specific tumor types. On the other hand, a more dynamic source of information is provided by experimental data from high-throughput sequencing, e.g., bulk sequencing or, more recently, single-cell sequencing. Bulk sequencing technologies provides genomics (bulk DNA-seq) or transcriptomics (bulk RNA-seq) information at tissue-level, measuring the average signal across a large population of cells. In contrast, single-cell sequencing technologies are able to provide the same genomics (scDNA-seq) and transcriptomics (scRNA-seq) information at a higher resolution, i.e.,at single-cell level, thus allowing heterogeneous system investigation. However, due to the higher cost of single-cell technologies, scRNA-seq datasets typically contains a lower number of patients with respect to the bulk ones.
In particular, two different datasets of human colorectal cancer (CRC) samples are used to model four specific real case scenarios, i.e.,CMS, with distinct biological characterization. Using bioinformatics analyses, we estimated 3 metrics for both datasets useful for model parameter setting. Additional information about data availability and bioinformatics analyses used is provided below.

S3.1 – Source of data

TCGA database

The Cancer Genome Atlas (TCGA) program provides bulk genomic and transcriptomic data for several human cancer, included human colorectal cancer. We downloaded the bulk gene expression data from BROAD Institute (https://gdac.broadinstitute.org) and we followed the same procedure used by Santana et al. to obtain transcripts of million (TPM) of 20472 valid HGNC symbols genes and 379 tumor samples (Lapuente-Santana et al., 2021). To estimate CMS group for each patient of TCGA cohort, we applied *CMScaller* classifier to bulk RNA-seq data (Eide et al., 2017).

SMC dataset

To inform model parameters, we used a publicly available scRNA-seq dataset of human colorectal cancer from the Samsung Medical Center (SMC) (Lee et al., 2020). The pre-processed gene expression matrix and relative metadata of 23 Korean patients are retrieved by GEO (GSE132465) together with genetic and clinical information from their study. In particular, for each individual we extracted the gene expression matrix in log natural TPM scale and its consensus molecular subtype CMS classification.

S3.2 – Index extraction

Tumor mutational burden, cell fractions and average expression levels of immune checkpoint genes are estimated for each patient of TCGA database and SMC dataset. Indexes estimation pipeline is described below.

Tumor mutational burden (TMB)

TMB measures the total number of nonsynonymous mutations per coding area of a sample genome (Mut/Mb) and can be estimated from bulk DNA-seq experiments.
The TMB of colorectal TCGA dataset is retrieved by NCI Genomic Data Commons (GDC) portal (Grossman et al., 2016), resulting from Thorsson et al. analysis of bulk whole-genome sequencing

(Thorsson et al., 2018). The non-silent mutational load in mut/Mb were extracted from "mutational-load_update.txt" (https://gdc.cancer.gov/about-data/publications/panimmune).
Moreover, number of mutations was retrieved for each SMC individuals from Lee Ho et al. analysis on bulk whole-genome sequencing experiment (Lee et al., 2020).

Cell fraction estimation

Cell fractions can be estimated from bulk RNA-seq experiments using deconvolutional methods (Finotello and Trajanoski, 2018), as well as from single-cell RNA-seq experiments using clustering and cell type annotation tools. However, both bulk and single-cell approaches have some limitations, mainly the need for a prior and reliable references on the cell type to estimate cell proportions. Therefore, these methods may fail to perform accurately when data contains rare, unknown, or uncharacterized cell types. However, single-cell technologies also rely on the poor efficiency of isolation protocols.
In order to obtain robust cell fraction estimates, we used a consensus approach based on six deconvolution methods accessible through the immunedeconv package (Sturm et al., 2019): quanTIseq (Finotello et al., 2019) , EPIC (Racle et al., 2017), ConsensusTME (Jimenez-Sanchez et al., 2019), xCell (Aran et al., 2017), TIMER (Li et al., 2016), and MCP-counter (Becht et al., 2016). quanTIseq was used to estimate cell fractions for $CD8^+$ T cells, B cells, Tregs, M1 and M2 macrophages, which showed high correlation with the other deconvolution methods (see Figure S6). EPIC was used to estimate CAFs (absent in quanTIseq signature), NK cells (low consensus agreement for quanTIseq), and tumor cells (high agreement with quanTIseq estimates, but more accurate as they do not include endothelial and epithelial cells), and normal cells (endothelial cells). Treg and NK cell fractions that were given a null score by xCell, were set to zero. Given the low agreement of EPIC and quanTIseq DC fractions compared to other methods, we used a three-step consensus approach: 1) we scaled in the 0-1 range DC scores obtained with xCell, MCP-counter, and TIMER; 2) we took the median; and 3) we rescale it to span the range of values covered by quanTIseq. Finally, cell fraction in each sample were rescaled to sum up to 100%.
In parallel, cell fraction estimation for SMC dataset was obtained from clustering and cell type annotation analysis performed by Lee Ho et al. It includes six major cell type classes, i.e., tumor, stroma, myeloid, T cells, B cells, mast cells, in turn divided into cell subtypes.

Expression of inhibitory immune checkpoint genes

One of the strategies employed by the tumor to evade immune response is the upregulation of inhibitory immune checkpoint molecules, which suppresses T cell activation.
The average expression level of genes encoding for these molecules can be computed at tissue-level from bulk RNA-seq of TCGA cohort and at cancer cell-level from scRNA-seq for each SMC individual.
A comprehensive list of inhibitory immune checkpoint genes (proteins) is obtained upon literature review: CD274 (PD-L1), PDCD1LG2 (PDL2), CD200R1, VSIR (VISTA), HLA-DPA1, HLA-DPB1, HLA-DQA1, HLA-DQA2, HLA-DQB1, HLA-DRA, HLA-DRB1, IGSF11 (VSIG3), CLEC4G (LSECTIN), TNFRSF14 (HVEM), PTDSS1 (PtdSer), LGALS1 (galectin 1), NECTIN2 (CD112), CSF1R (CD115) (Donini et al., 2018; Qin et al., 2019; Huang et al., 2019).

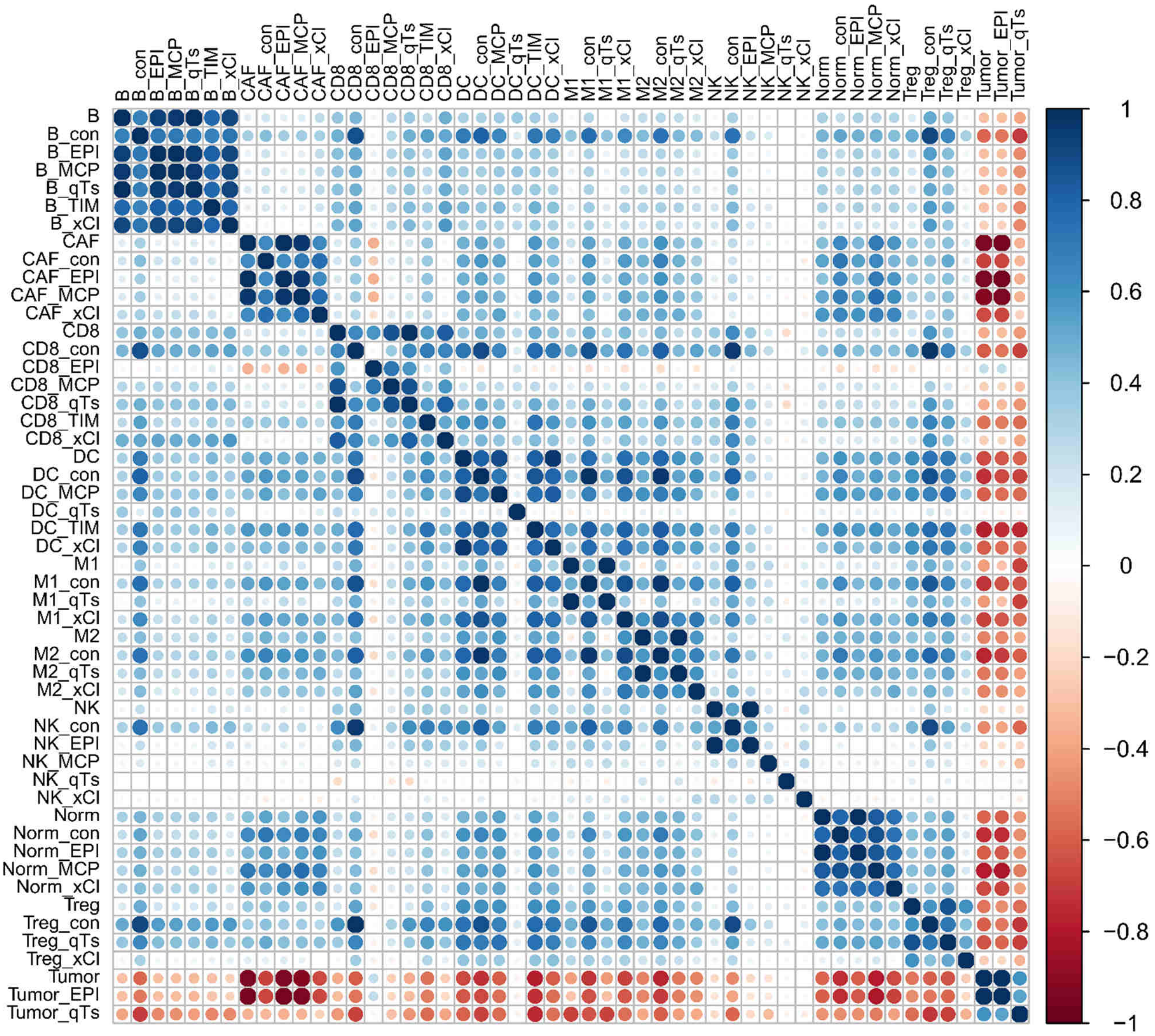

Figure S6 – Deconvolution method correlation. Comparison between deconvolution methods used to estimate cell fractions. Correlation values between different cell fraction estimations are represent in color-scale. Labels are represented in the form of <*cell type*>_<*deconvolution method*>. Methods considered in the analysis are ConsensusTME (referred as *con*), EPIC (referred as *EPI*), MCP-counter (referred as *MCP*), TIMER (referred as *TIM*), and xCell (referred as *xCl*). Labels without deconvolutional method specified represent cell fraction estimates resulting from the consensus approach.

Section S4 – Additional information on CMS parameter setting using different sources

In order to simulate specific features of each CMS, model parameters are informed using literature and several high-throughput experiments, i.e., bulk and single-cell experiments. Several bioinformatics analyses can be used to estimate metrics useful for parameter setting, i.e., tumor mutational burden, cell fraction and average gene expression profile. Additional information about data source of CRC patients and index estimation is provided in Section S3. In this section, we discuss the rationale behind the parameter setting for simulating the different CMSs.

First, TMB index is used to set the mutational probability of losing/acquiring antigenicity, i.e., *tum_newantigen_rate*. Mutational load is estimated from bulk whole-genome sequencing experiments in TCGA cohort. TMB distribution across the 4 subtypes is provided in Figure S7.

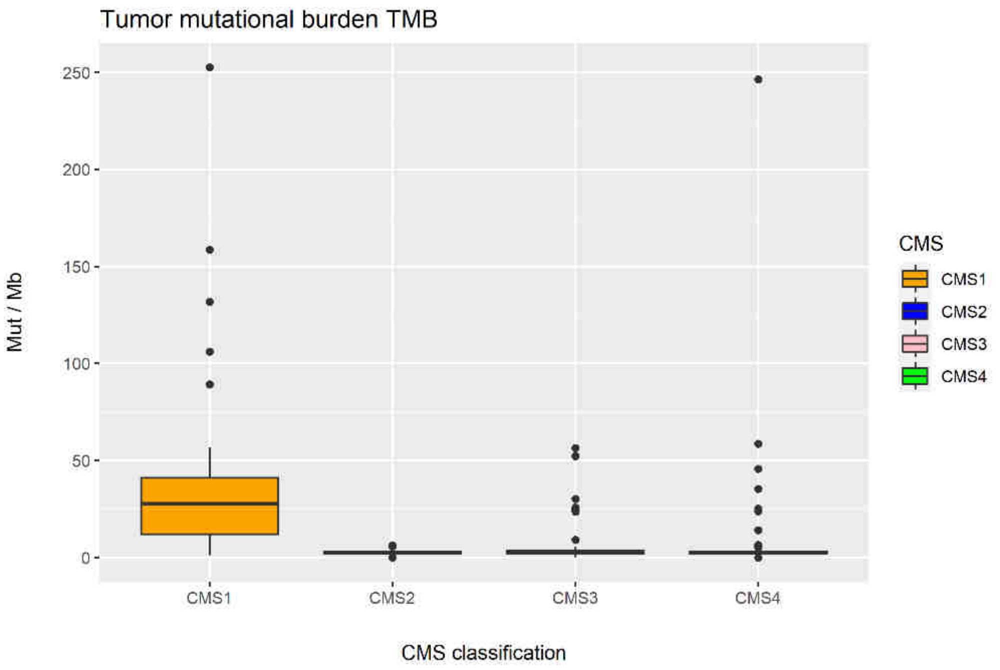

Figure S7 – Distribution of TMB values (Mut/Mb) across CMS derived from bulk whole genome experiments in TCGA cohort. *The boxplot describes the median and IQR. Colors identify the different groups: yellow for CMS1, blue for CMS2, pink for CMS3, and green for CMS4.*

Second, cell fractions are estimated from bulk RNA-seq data of TCGA cohort. In particular, CAF and Treg cell proportion estimates are used to tune recruitment probabilities through *inhib_CAF_recruit_par* and *inhib_TREG_recruit_par* parameters, as shown in Table S3. Moreover, immune cell fraction estimate, i.e., proportions of all immune-related cell types (NK cells, DC cells, CD8+ cells, B cells, macrophages and Treg cells), are used to set tumor mutation probability of acquiring mutation that make cancer cell release molecules repelling IS locally. CMS parameter setting is obtained by following relative ranking of cell fractions estimates across CMS (Figures S8 and S9).

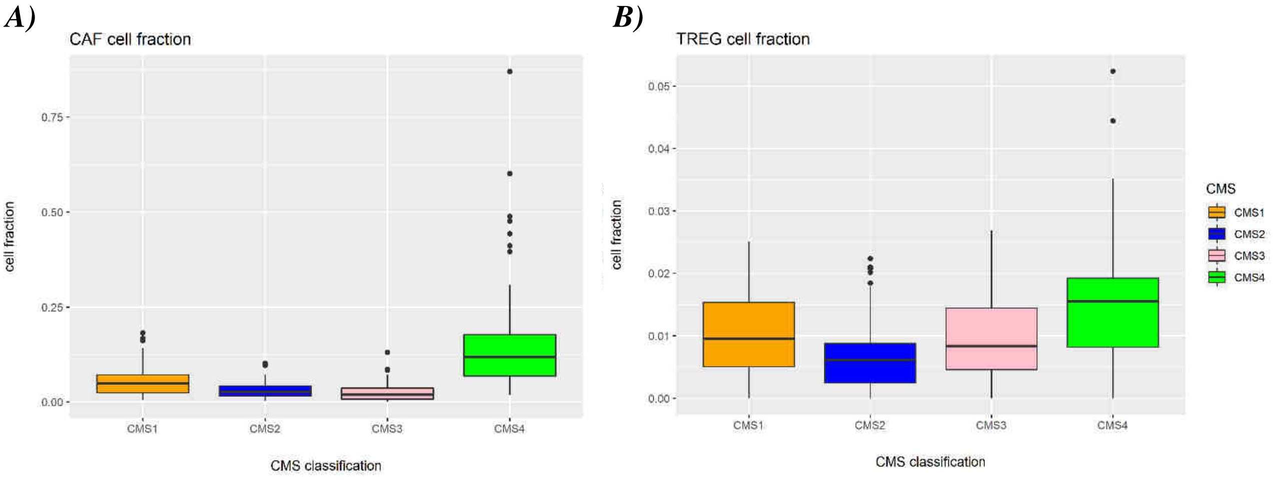

Figure S8 – Distribution of CAF and Treg cell fractions across CMS using bulk RNA-seq data in TCGA cohort. *A) Comparison of CAFs proportions between CMS. B) Comparison of Tregs proportions between CMS. The boxplot describes the median and IQR. Colors identify the different groups: yellow for CMS1, blue for CMS2, pink for CMS3, and green for CMS4.*

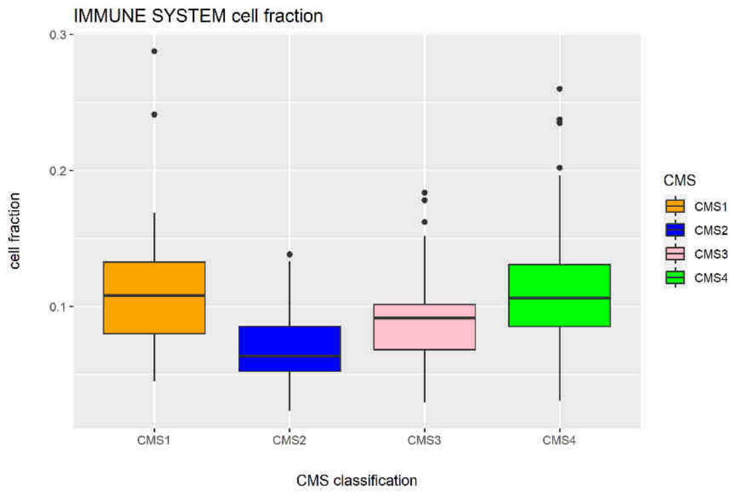

Figure S9 – Distribution of immune system cell fractions across CMS using bulk RNA-seq data in TCGA cohort. *The boxplot describes the median and IQR. Colors identify the different groups: yellow for CMS1, blue for CMS2, pink for CMS3, and green for CMS4.*

The mean expression level of PD-L1-like genes, i.e., inhibitory immune checkpoint genes listed in Section S3, is computed at tumor cell level from single-cell RNA sequencing data of SMC dataset. The relative distribution of values across the four subtypes (see Figure S10) is used to set the mutational probability of losing immunogenicity, i.e., *tum_pdlp_mut* parameter.

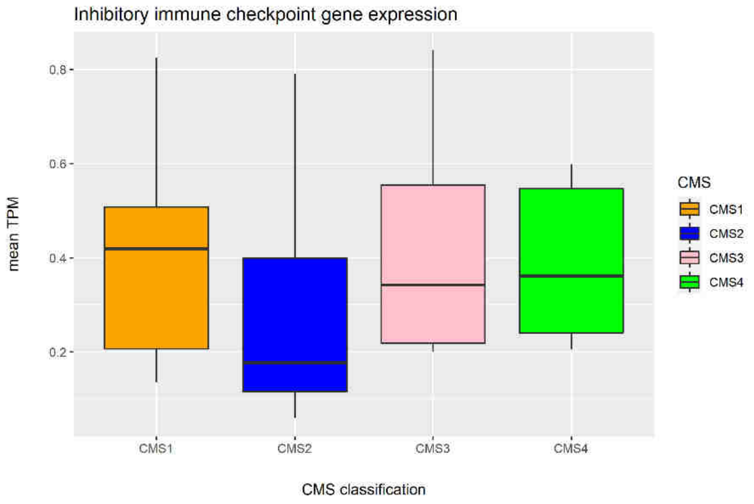

Figure S10 – Distribution of average PD-L1-like gene expression levels across CMS from scRNA-seq data in SMC dataset. *The boxplot describes the median and IQR. Colors identify the different groups: yellow for CMS1, blue for CMS2, pink for CMS3, and green for CMS4.*

The following table (Table S3) summarizes the parameter setting described above, as well as provides additional information about data sources used.

**Parameter**	**CMS1**	**CMS2**	**CMS3**	**CMS4**	**Description**	**Source**	**Dataset**	**Index**
tum_newantigen_rate	High (0.1)	Low (0.01)	Low (0.01)	Low (0.01)	Probability to induce a new antigen mutation	DNA seq	TGCA	TMB
tum_pdlp_rate	High (0.2)	Low (0.02)	High (0.2)	High (0.2)	Probability to gain a PD-L1-like mutation	scRNA-seq	SMC	Gene expres-sion
tum_adjchange_rate	Low (0.005)	High (0.05)	Mid (0.025)	Low (0.005)	Probability to acquire a mutation that make cancer cell release molecules that repel IS cells locally	RNA-seq	TGCA	Cell fraction
inhib_TREG_recruit_par	High (1)	High (1)	High (1)	Low (0.1)	Parameter related to probability of recruiting TREG in the domain	RNA-seq	TGCA	Cell fraction
inhib_CAF_recruit_par	High (2)	High (2)	High (2)	Low (0.2)	Parameter related to probability of recruiting CAF in the domain	RNA-seq	TGCA	Cell fraction
tum_ncons	Low (0.02)	Low (0.02)	High (0.2)	Low (0.02)	Glucose consumption of a tumor agent	literature	/	/

Table S3 –MAST parameters setting across CMS using as source of information both TGCA and SMC datasets.

Section S5 – Additional results of CMS simulations

In the section, additional analyses, and figures of CMS output models, using the data-driven setting explained in Section S4, are provided. In particular, the following figures (Figures S11-13) show, for each CMS scenario, the average counts over time of three agents of MAST, i.e.,tumor cells having acquired PD-L1-like mutation, namely PD-L1+ tumor cells, CTL cells and CAF cells.

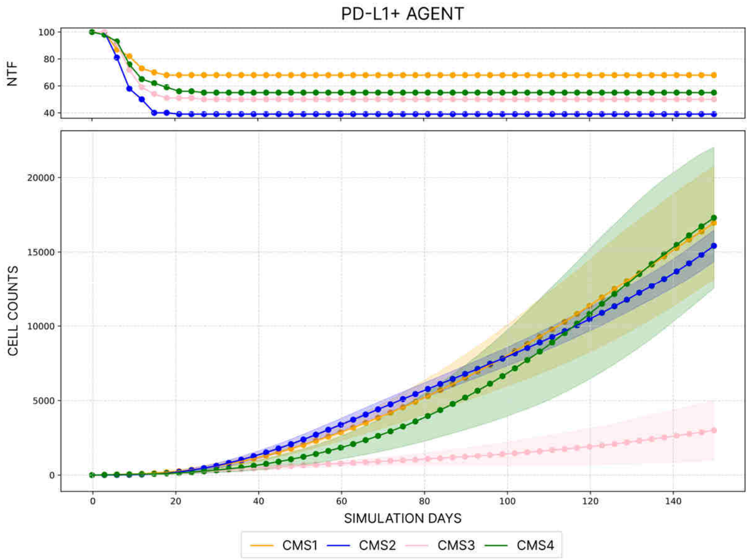

Figure S11 – Cardinality of PD-L1+ tumor cells over time for all CMS groups. *In the upper sub-graph, the number of not completely tumor free (NTF) simulations in a determined instant (day), i.e., simulations having at least one cancer agent in the domain, is showed for each CMS. In the below subgraph, the continuous line represents the average number of PD-L1+ cells and the shaded area represents its variability (± standard deviation), computed across all NTF simulations of a given CMS group. Legend below represents CMS-color association. This graphical representation is generated using MAST.*

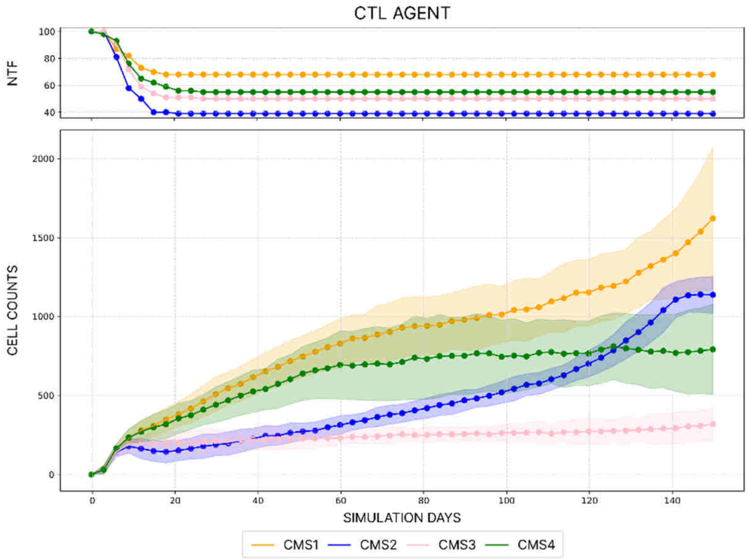

Figure S12 - Cardinality of CTL cells over time for all CMS groups. *In the upper subgraph, the number of not completely tumor free (NTF) simulations in a determined instant (day), i.e., simulations having at least one cancer agent in the domain, is showed for each CMS. In the below subgraph, the continuous line represents the average number of CTL cells, and the shaded area represents its variability (± standard deviation), computed across all NTF simulations of a given CMS group. Legend below represents CMS-color association. This graphical representation is generated using MAST.*

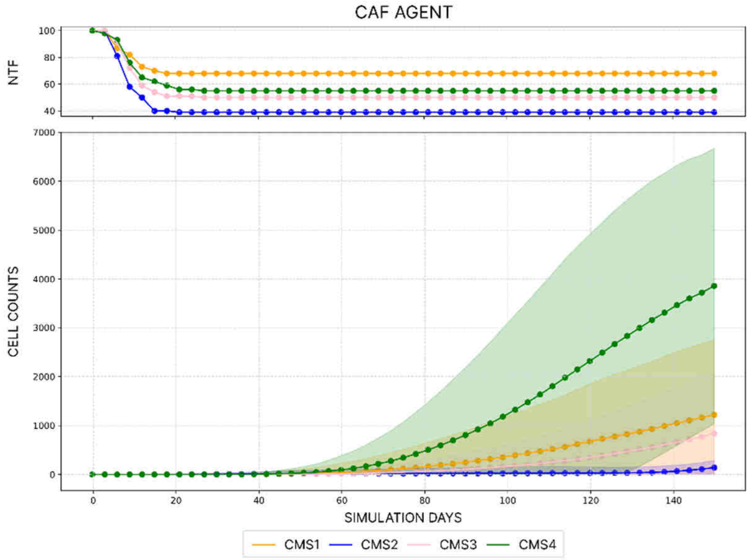

Figure S13 – Cardinality of CAF cells over time for all CMS groups. *In the upper subgraph, the number of not completely tumor free (NTF) simulations in a determined instant (day), i.e., simulations having at least one cancer agent in the domain, is showed for each CMS. In the below subgraph, the continuous line represents the average number of CAF cells, and the shaded area represents its variability (± standard deviation), computed across all NTF simulations of a given CMS group. Legend below represents CMS-color association. This graphical representation is generated using MAST.*

Survival analysis was performed on TGCA collective. Clinical data were downloaded from NCI Genomic Data Commons (GDC) portal resulting from PanCanAtlas project study (Weinstein et al., 2013; Grossman et al., 2016).

In particular, survival outcome data for each patient were extracted from “TCGA-CDR-SupplementalTableS1.xlsx” file (https://gdc.cancer.gov/about-data/publications/pancanatlas). As suggested by The Cancer Genome Atlas Research Network, progression-free interval PFI is used as clinical outcome endpoint for the subsequent survival analysis (Figure S14) (Liu et al., 2018). Log rank test for difference in PFI across CRC subtypes gives a pvalue of $p = 0.0026$, indicating that the CMS groups differ significantly.

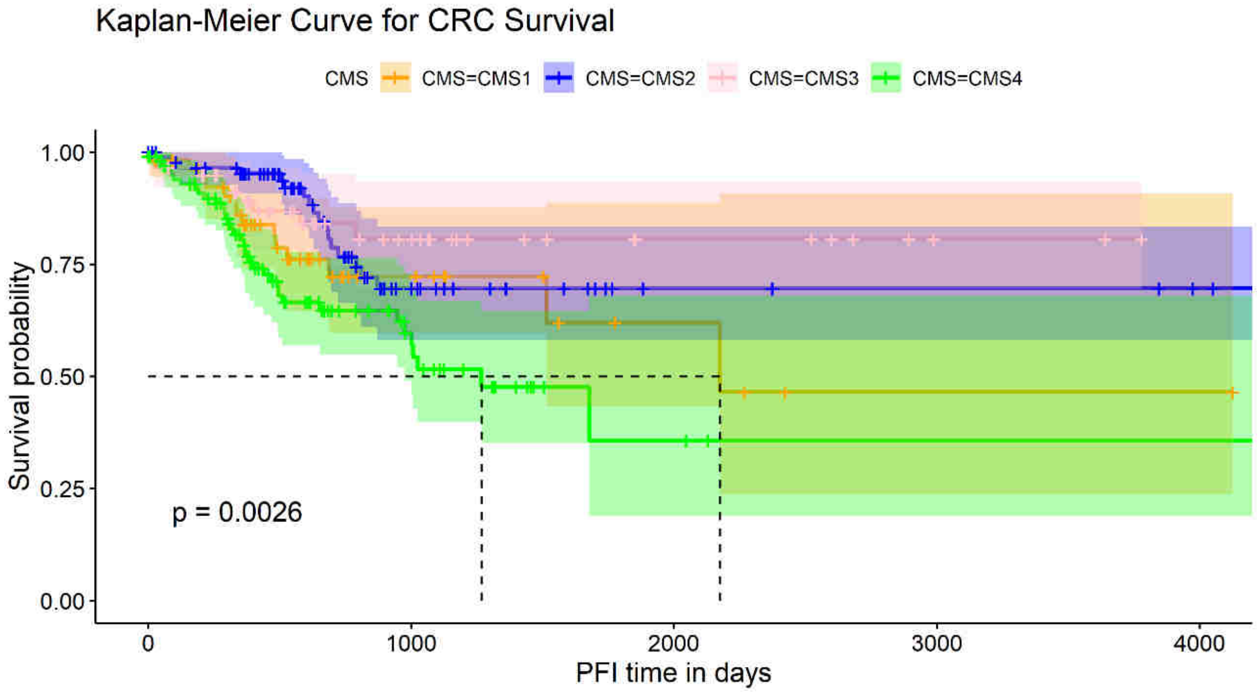

***Figure S14 – Clinical endpoint analysis of PFI in TCGA cohort**. Prognostic value of CMS1 (yellow), CMS2 (blue), CMS3 (pink), CMS4 (green) with Kaplan-Meier plot for progression free-interval (PFI). Hazard ratios and 95% confidence interval are showed.*

Section S6 – Additional information on the effect of specific parameter variation on TME

In this section, results and analysis of simulating different mutational, metabolic and stromal conditions on tumor microenvironment are provided.

S6.1 Effect of antigenicity

In order to exploit the effect of loss/acquisition of antigenicity on tumor progression, we simulated various level of tumor hypermutation by increasing *tum_newantigen_rate* parameter from the default value (i.e., 0.01) of 1-fold, 2-fold, 4-fold, 8-fold, 10-fold and 16-fold. Tumor development in space and time for each CMS are illustrated in Figures S15-22.
As the *tum_newantigen_rate* parameter increases, the number of CTLs over time increases in scenarios CMS1, CMS2 and CMS4 (Figures S15. S17 and S21), whereas for CMS3 scenario, which has a low immune response, the effect cannot be appreciated (Figure S19). Indeed, it is expected that the increasing probability of acquiring new antigens results in a more elicited antigen-specific immune response. On the other hand, the acquisition of new antigens results also in an increasing in progressive tumor outcome rate, represented by the upper subgraphs of Figures S15, S17, S19 and S21, suggesting a dual role of antigenicity; loss of antigenicity provides an immune escape mechanism by decreasing immune system activation, while at the same time make the "reduced" immune system response more effective since the mutational rate is slower.

S6.1.1 Immune molecular subtype (CMS1)

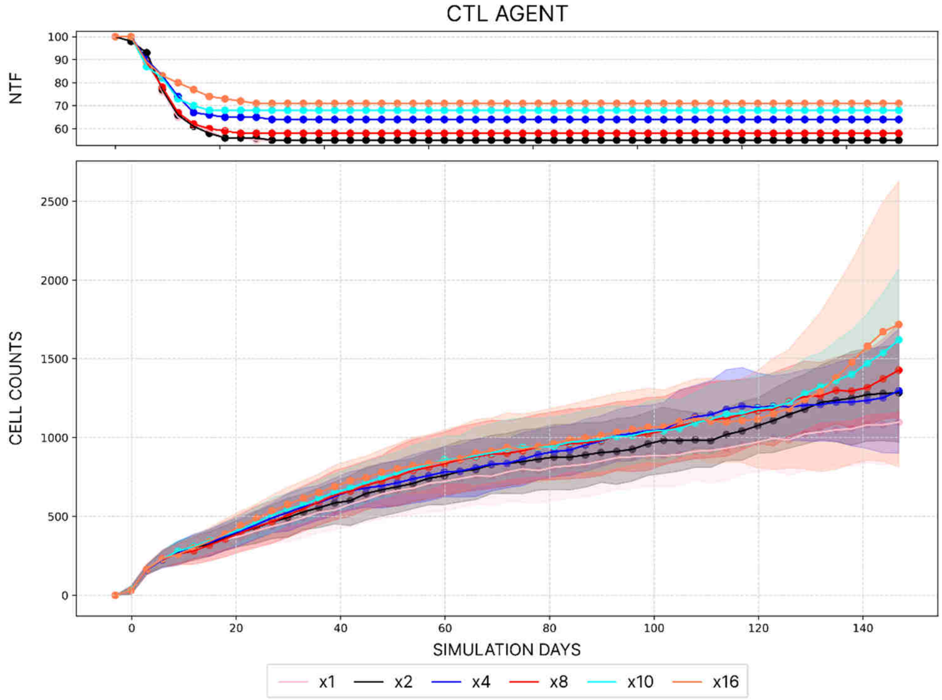

Figure S15 – Cardinality of CTL cells over time at increasing antigenicity rate in CMS1 scenario. *In the upper subgraph, the number of not completely tumor-free (NTF) simulations in a determined instant (day), i.e., simulations having at least one cancer agent in the domain, is showed for each antigenicity scenario. In the below subgraph, the continuous line represents the average number of CTL cells, and the shaded area represents its variability (± standard deviation), computed across all NTF simulations of a given antigenicity scenario. This graphical representation is generated using MAST.*

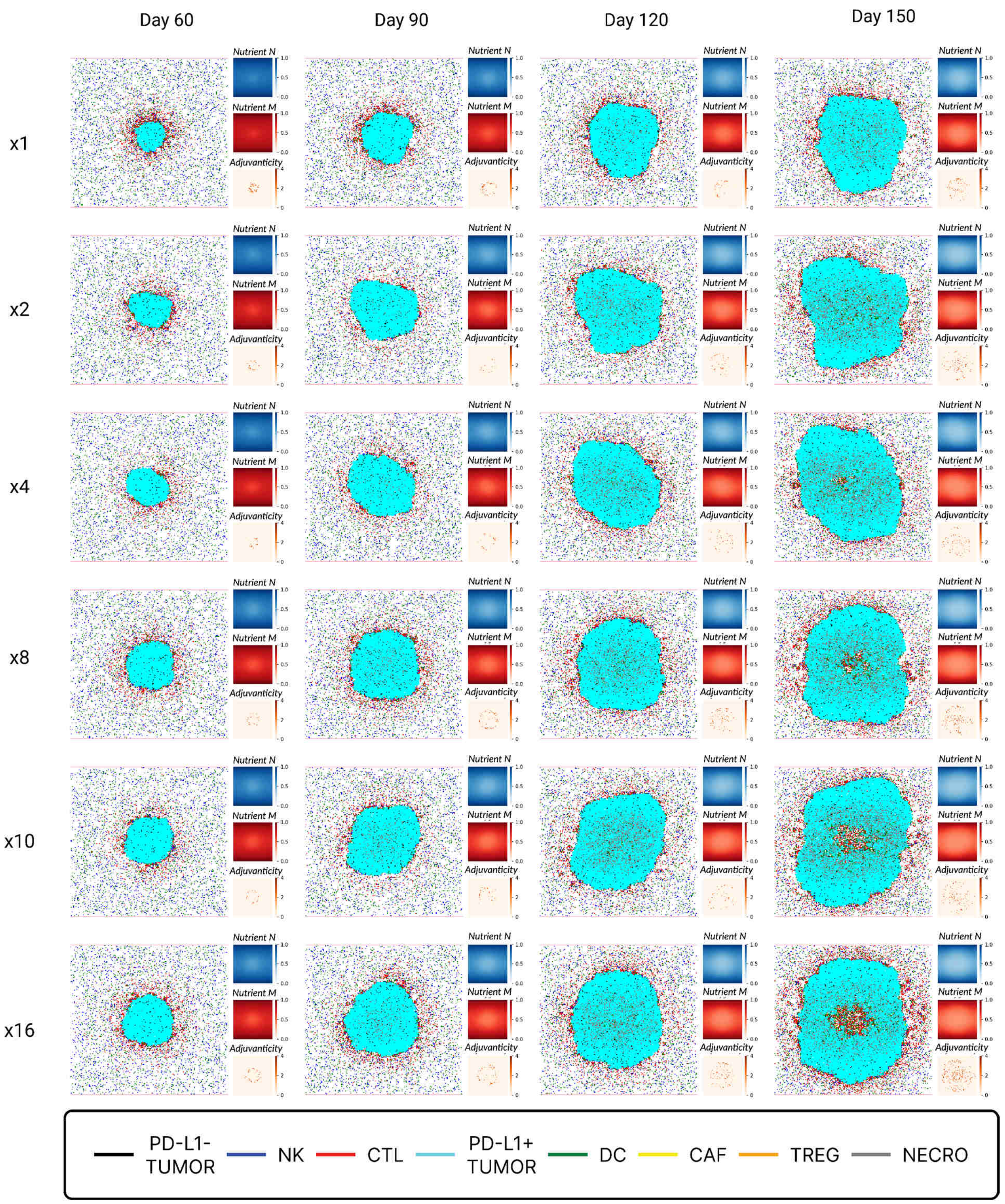

Figure S16 – Spatio-temporal evolution of tumor progression at different antigenicity rate in CMS1 scenario. *Spatio-temporal evolution of CMS1 with increased probability of tumor antigenicity. Each row of the grid represents 1 of 100 simulations performed for the specific parameter value. In addition, tumor progression on days 60, 90, 120, and 150 can be observed from left to right. Each panel represents MAST environment and displays: i) in the left, spatial distribution of different agents (legend shows agent-color association); ii) in the right, spatial distribution of nutrient N (e.g., glucose), nutrient M (e.g., oxygen), and adjuvanticity signal respectively from top to bottom subplots. All graphical representations are generated using MAST.*

S6.1.2 Canonical molecular subtype (CMS2)

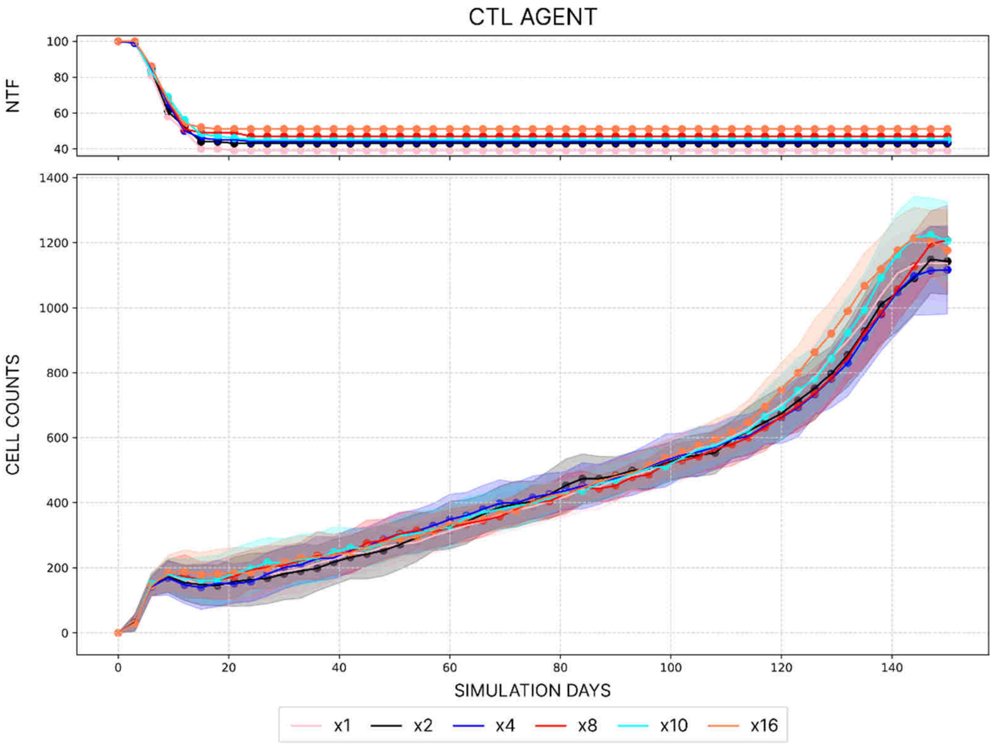

Figure S17 – Cardinality of CTL cells over time at increasing antigenicity rate in CMS2 scenario. *In the upper subgraph, the number of not completely tumor-free (NTF) simulations in a determined instant (day), i.e., simulations having at least one cancer agent in the domain, is showed for each antigenicity scenario. In the below subgraph, the continuous line represents the average number of CTL cells, and the shaded area represents its variability (± standard deviation), computed across all NTF simulations of a given antigenicity scenario. This graphical representation is generated using MAST.*

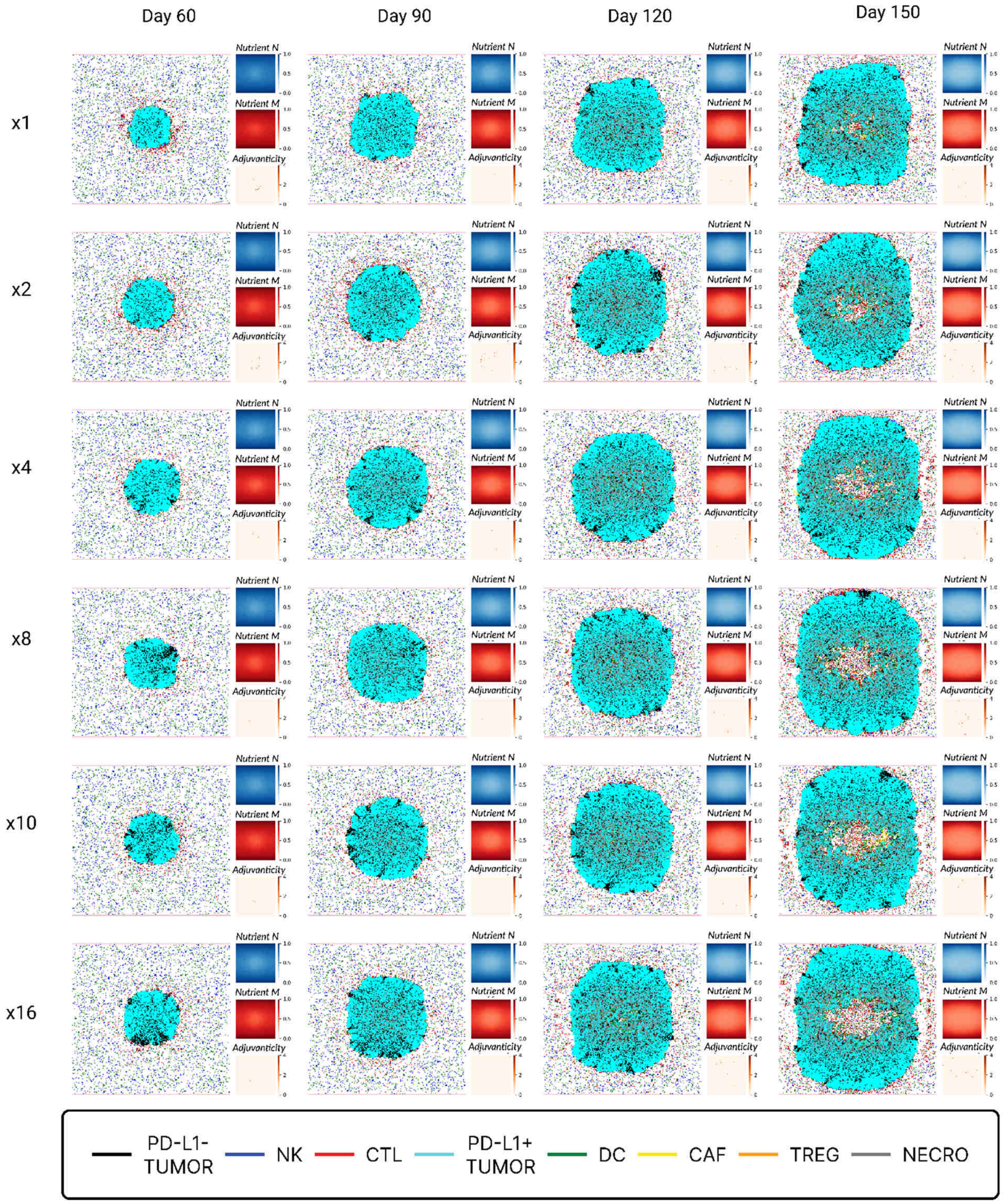

Figure S18 – Spatio-temporal evolution of tumor progression at different antigenicity rate in CMS2 scenario. *Spatio-temporal evolution of CMS1 with increased probability of tumor antigenicity. Each row of the grid represents 1 of 100 simulations performed for the specific parameter value. In addition, tumor progression on days 60, 90, 120, and 150 can be observed from left to right. Each panel represents MAST environment and displays: i) in the left, spatial distribution of different agents (legend shows agent-color association); ii) in the right, spatial distribution of nutrient N (e.g., glucose), nutrient M (e.g., oxygen), and adjuvanticity signal respectively from top to bottom subplots. All graphical representations are generated using MAST.*

S6.1.3 Metabolic molecular subtype (CMS3)

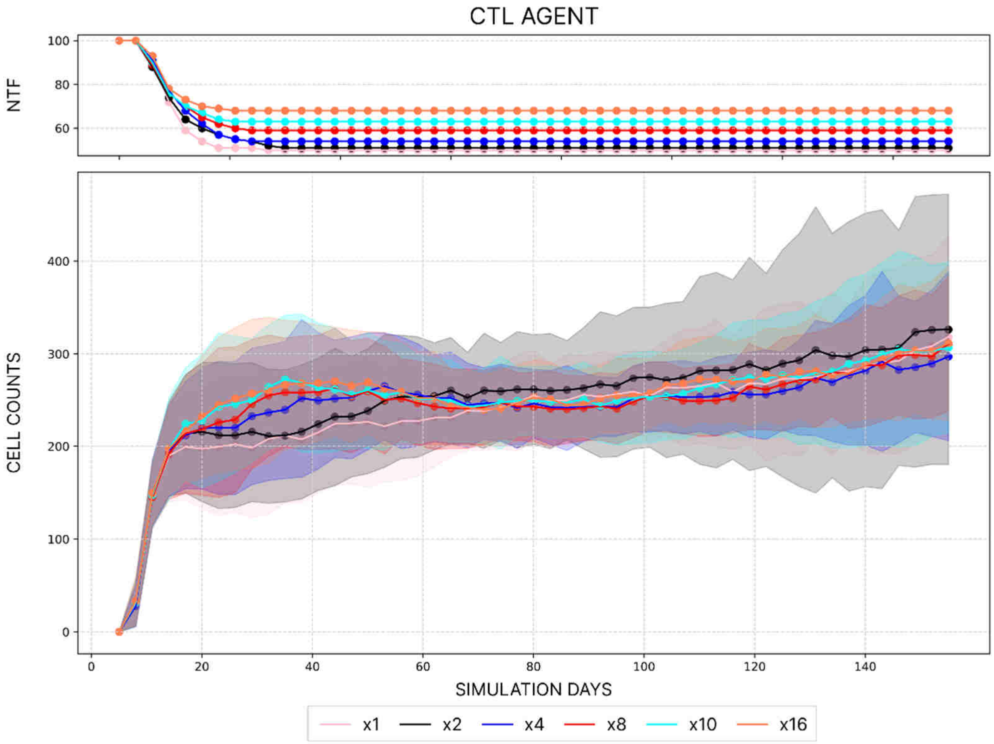

Figure S19 – Cardinality of CTL cells over time at increasing antigenicity rate in CMS3 scenario. *In the upper subgraph, the number of not completely tumor-free (NTF) simulations in a determined instant (day), i.e., simulations having at least one cancer agent in the domain, is showed for each antigenicity scenario. In the below subgraph, the continuous line represents the average number of CTL cells, and the shaded area represents its variability (± standard deviation), computed across all NTF simulations of a given antigenicity scenario. This graphical representation is generated using MAST.*

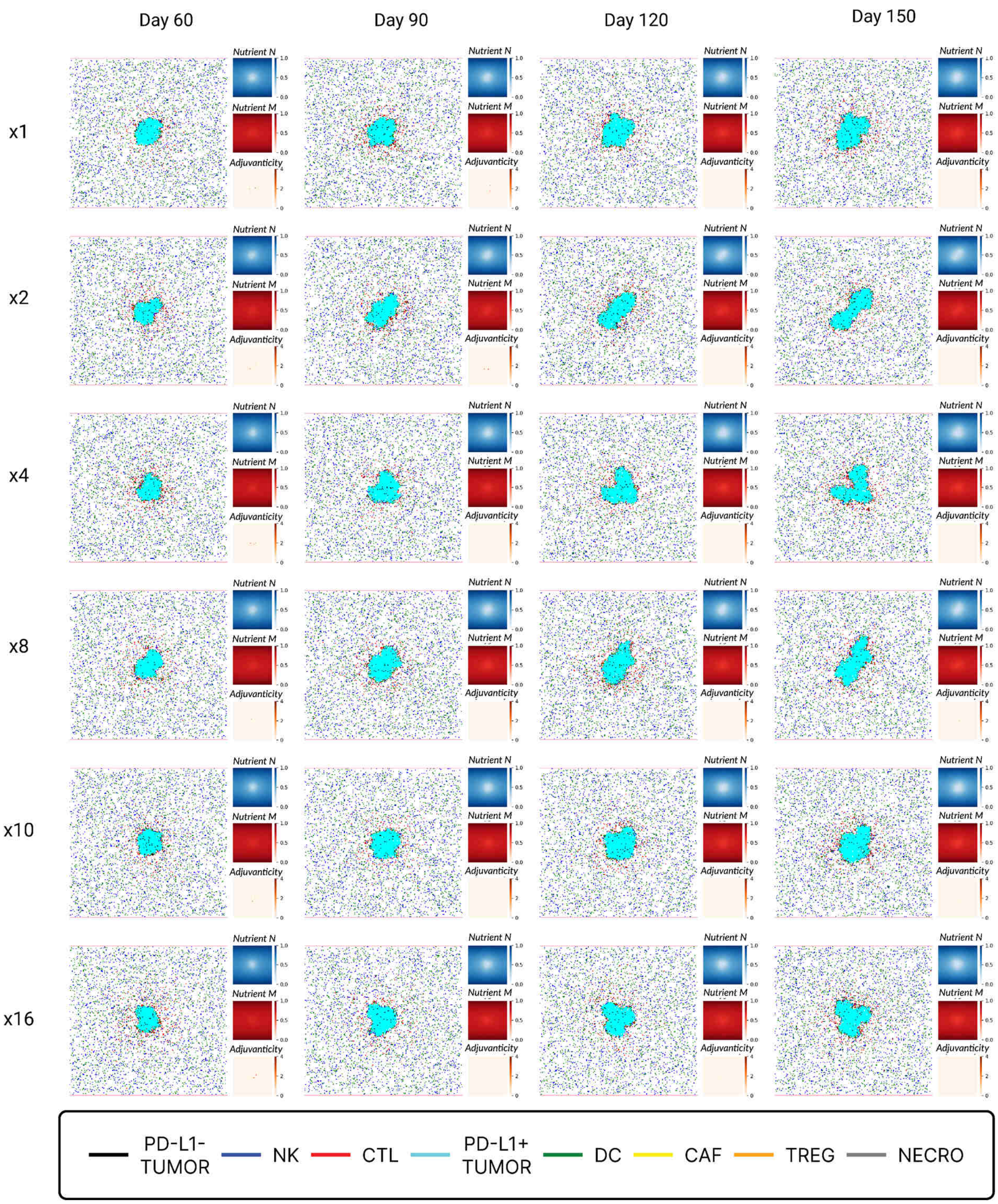

Figure S20 – Spatio-temporal evolution of tumor progression at different antigenicity rate in CMS3 scenario. *Spatio-temporal evolution of CMS1 with increased probability of tumor antigenicity. Each row of the grid represents 1 of 100 simulations performed for the specific parameter value. In addition, tumor progression on days 60, 90, 120, and 150 can be observed from left to right. Each panel represents MAST environment and displays: i) in the left, spatial distribution of different agents (legend shows agent-color association); ii) in the right, spatial distribution of nutrient N (e.g., glucose), nutrient M (e.g., oxygen), and adjuvanticity signal respectively from top to bottom subplots. All graphical representations are generated using MAST.*

S6.1.4 Mesenchymal molecular subtype (CMS4)

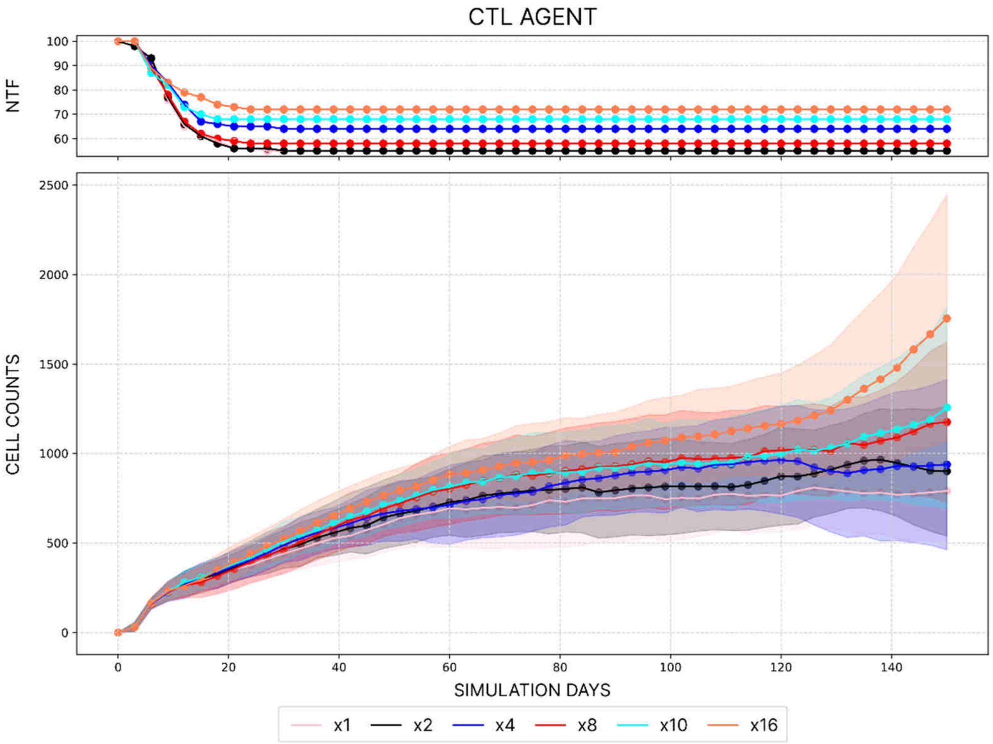

Figure S21 – Cardinality of CTL cells over time at increasing antigenicity rate in CMS4 scenario. *In the upper subgraph, the number of not completely tumor-free (NTF) simulations in a determined instant (day), i.e., simulations having at least one cancer agent in the domain, is showed for each antigenicity scenario. In the below subgraph, the continuous line represents the average number of CTL cells, and the shaded area represents its variability (± standard deviation), computed across all NTF simulations of a given antigenicity scenario. This graphical representation is generated using MAST.*

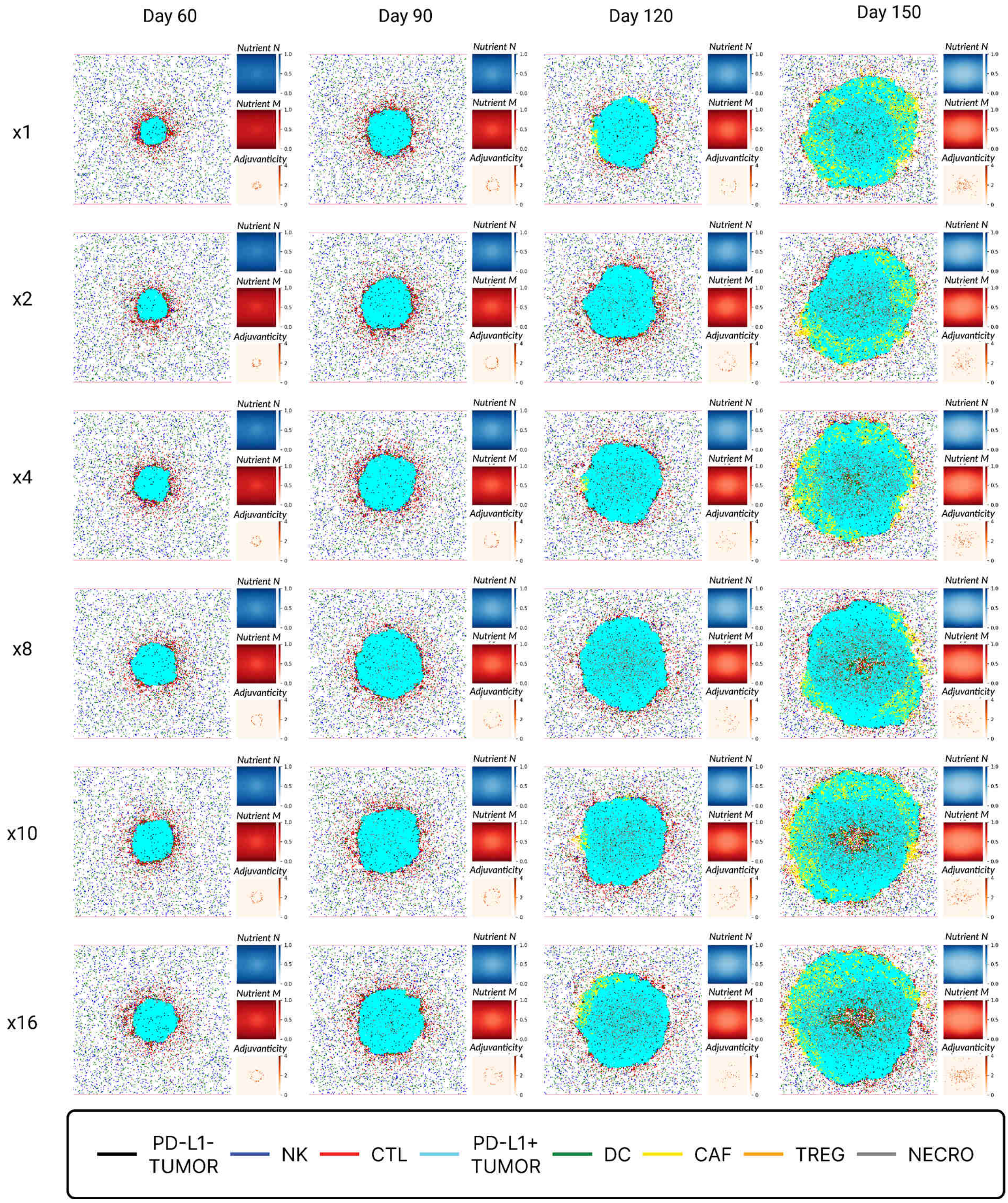

Figure S22 – Spatio-temporal evolution of tumor progression at different antigenicity rate in CMS4 scenario. *Spatio-temporal evolution of CMS1 with increased probability of tumor antigenicity. Each row of the grid represents 1 of 100 simulations performed for the specific parameter value. In addition, tumor progression on days 60, 90, 120, and 150 can be observed from left to right. Each panel represents MAST environment and displays: i) in the left, spatial distribution of different agents (legend shows agent-color association); ii) in the right, spatial distribution of nutrient N (e.g., glucose), nutrient M (e.g., oxygen), and adjuvanticity signal respectively from top to bottom subplots. All graphical representations are generated using MAST.*

S6.2 Effect of immunogenicity

In order to investigate the effect of loss of immunogenicity on tumor progression, we varied *tum_pdlp_rate* parameter from the default value (i.e.,0.02) of 1-fold, 2-fold, 4-fold, 8-fold, 10-fold and 16-fold. Tumor development in space and time for each CMS scenario are illustrated in Figures S23-30.

We can observe that as the *tum_pdlp _rate* parameter increases, it results in a worsening on progression of the tumor represented by the increasing number of not completely tumor-free simulations, highlighted in the upper subgraph of Figures S23, S25, S27 and S29. This is expected since tumor is applying an immune escape mechanism by reducing its probability to be killed by CTLs. Moreover, it is possible to notice how tumor having not acquired a PD-L1-like mutation, namely PD-L1- tumor, drastically decreases across simulations both temporally and spatially (black cells in Figures S24, S26, S28 and S30).

S6.2.1 Immune molecular subtype (CMS1)

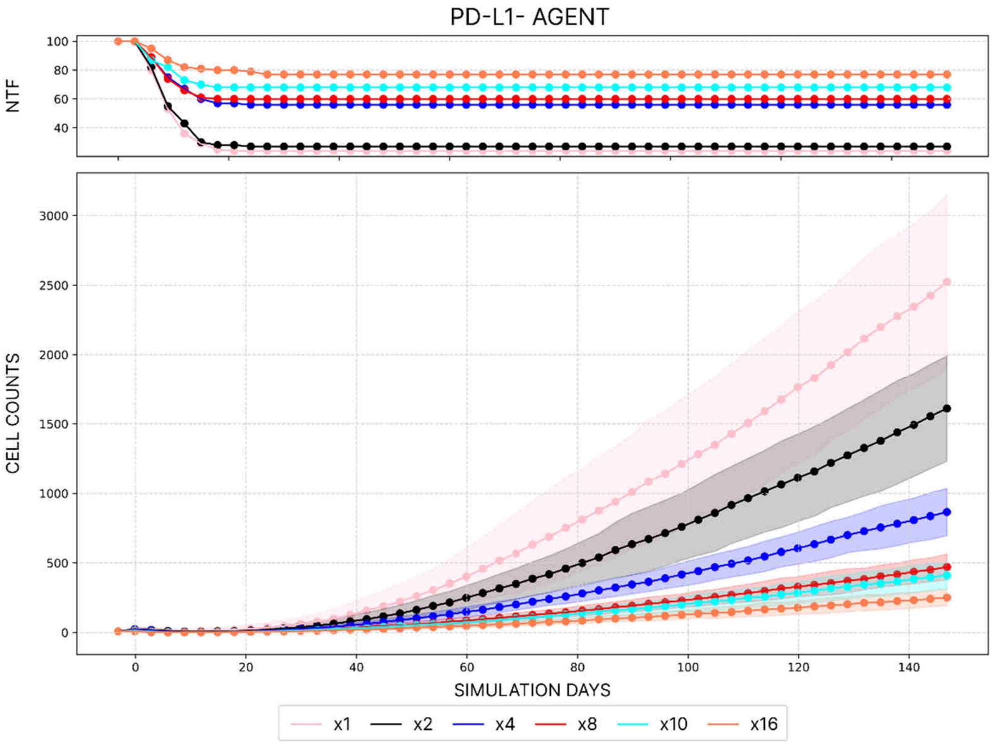

Figure S23 – Cardinality of PD-L1- tumor cells over time at increasing immunogenicity loss rate in CMS1 scenario. *In the upper subgraphs, the number of not completely tumor-free (NTF) simulations in a determined instant (day), i.e., simulations having at least one cancer agent in the domain, is showed for each immunogenicity scenario. In the below subgraph, the continuous line represents the average number of PD-L1- tumor cells and the shaded area represents its variability, computed across all NTF simulations of each immunogenicity scenario. This graphical representation is generated using MAST.*

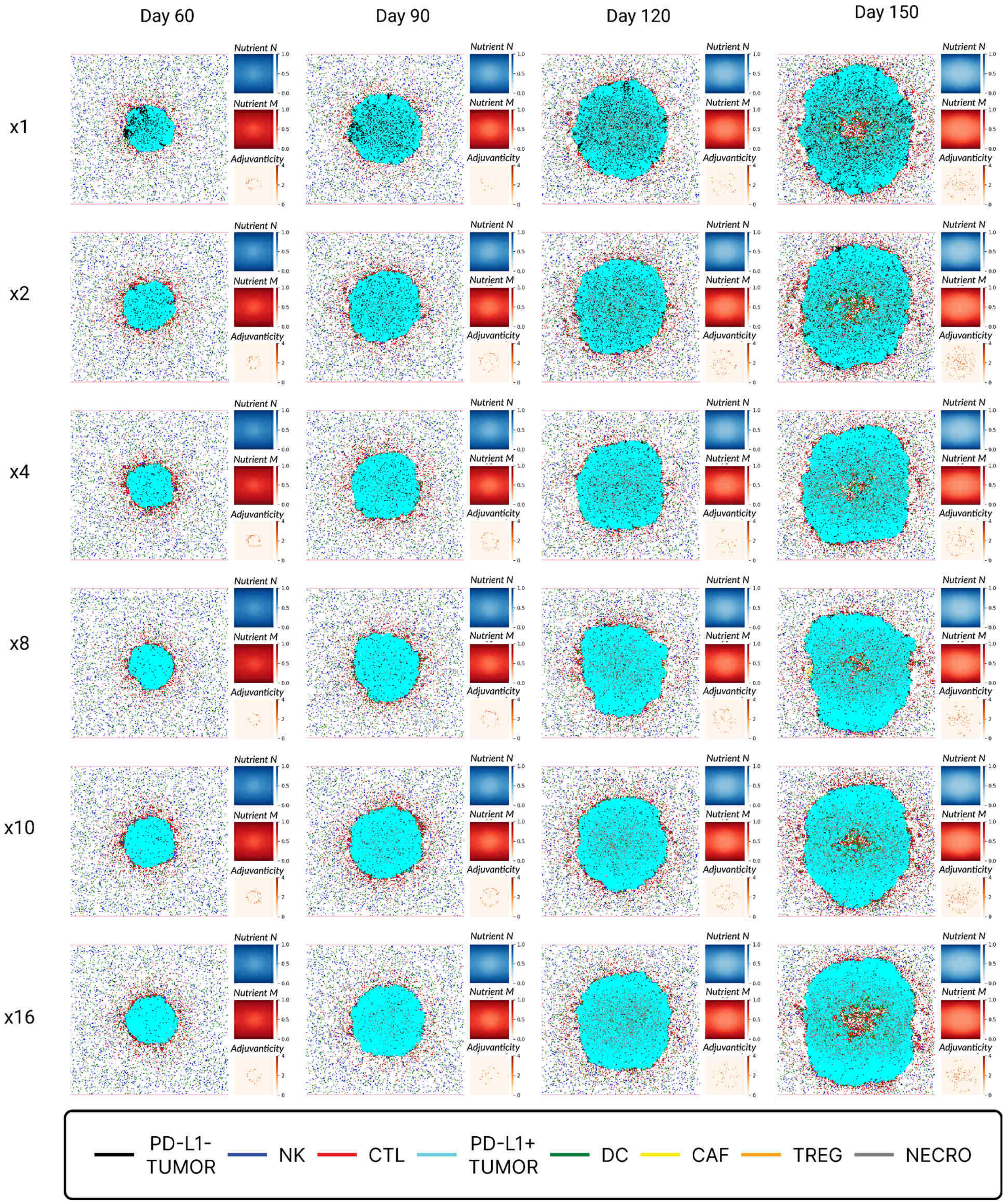

Figure S24 – Spatio-temporal evolution of tumor progression at increasing immunogenicity loss rate in CMS1 scenario. *Spatio-temporal evolution of CMS1 with increased probability of losing immunogenicity. Each row of the grid represents 1 of 100 simulations performed for the specific parameter value. In addition, tumor progression on days 60, 90, 120, and 150 can be observed from left to right. Each panel represents MAST environment and displays: i) in the left, spatial distribution of different agents (legend shows agent-color association); ii) in the right, spatial distribution of nutrient N (e.g., glucose), nutrient M (e.g., oxygen), and adjuvanticity signal respectively from top to bottom subplots. All graphical representations are generated using MAST.*

S6.2.2 Canonical molecular subtype (CMS2)

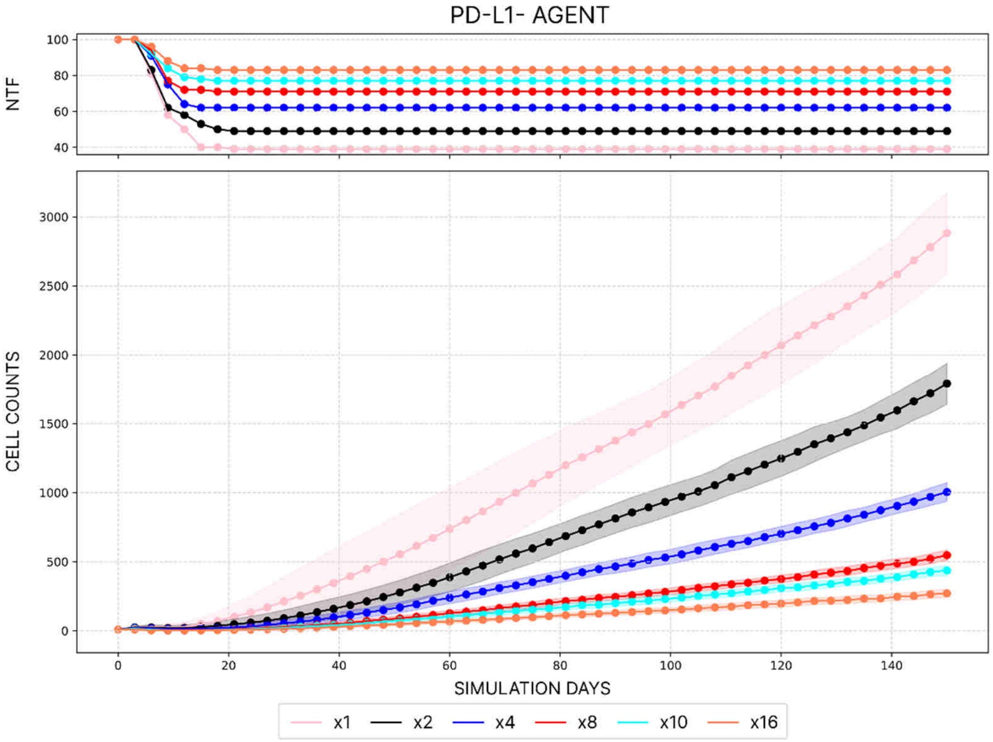

Figure S25 – Cardinality of PD-L1- tumor cells over time at increasing immunogenicity loss rate in CMS2 scenario. *In the upper subgraphs, the number of not completely tumor-free (NTF) simulations in a determined instant (day), i.e., simulations having at least one cancer agent in the domain, is showed for each immunogenicity scenario. In the below subgraph, the continuous line represents the average number of PD-L1- tumor cells and the shaded area represents its variability, computed across all NTF simulations of each immunogenicity scenario. This graphical representation is generated using MAST.*

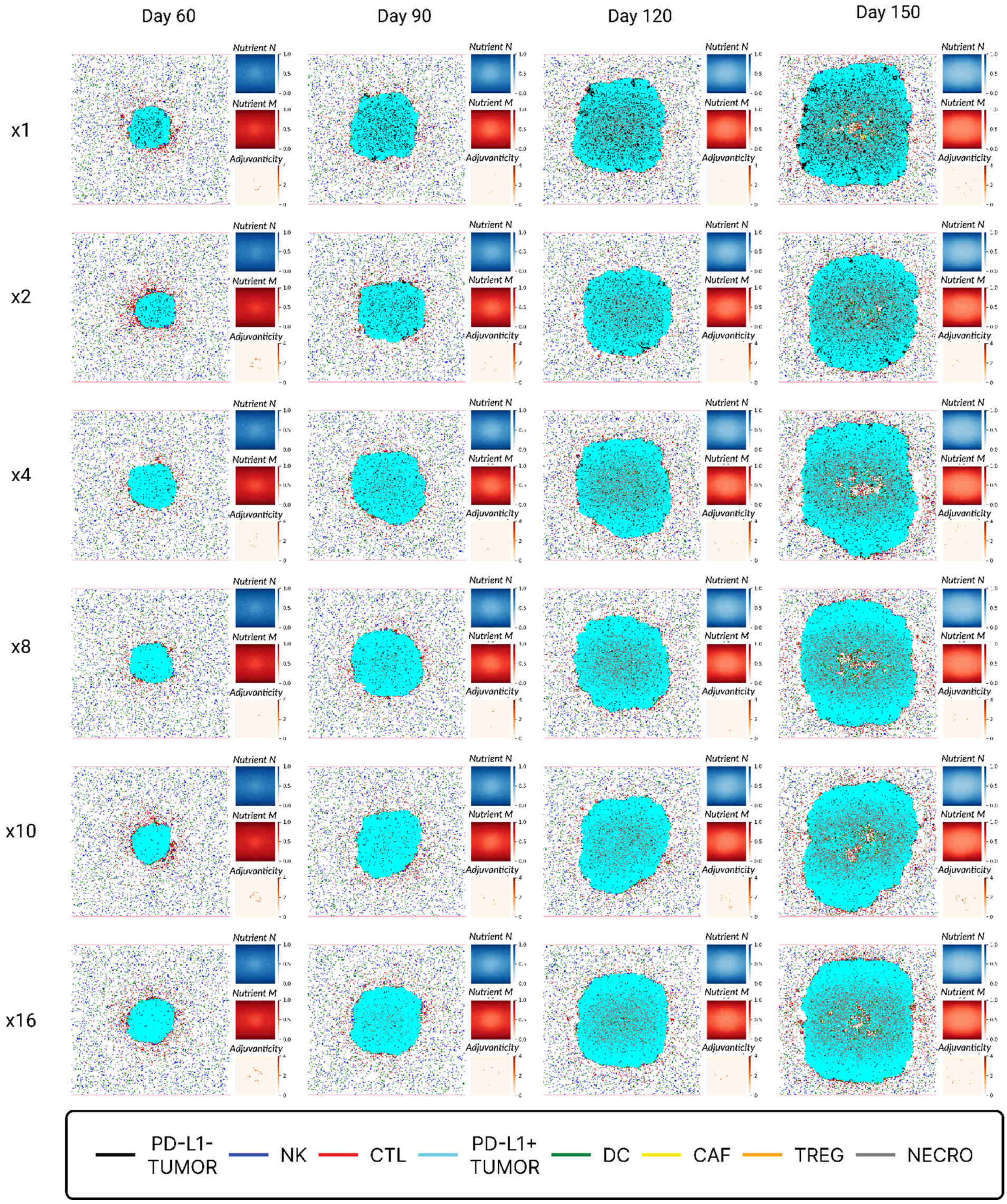

Figure S26 – Spatio-temporal evolution of tumor progression at increasing immunogenicity loss rate in CMS2 scenario. *Spatio-temporal evolution of CMS1 with increased probability of losing immunogenicity. Each row of the grid represents 1 of 100 simulations performed for the specific parameter value. In addition, tumor progression on days 60, 90, 120, and 150 can be observed from left to right. Each panel represents MAST environment and displays: i) in the left, spatial distribution of different agents (legend shows agent-color association); ii) in the right, spatial distribution of nutrient N (e.g., glucose), nutrient M (e.g., oxygen), and adjuvanticity signal respectively from top to bottom subplots. All graphical representations are generated using MAST.*

S6.2.3 Metabolic molecular subtype (CMS3)

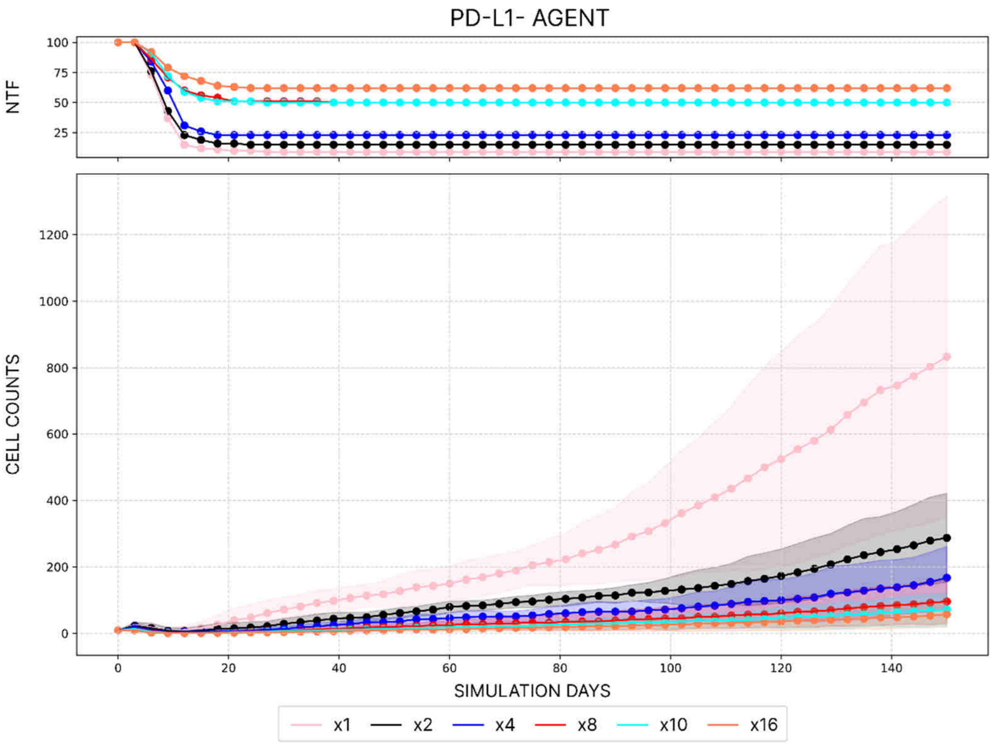

Figure S27 – Cardinality of PD-L1- tumor cells over time at increasing immunogenicity loss rate in CMS3 scenario. *In the upper subgraphs, the number of not completely tumor-free (NTF) simulations in a determined instant (day), i.e., simulations having at least one cancer agent in the domain, is showed for each immunogenicity scenario. In the below subgraph, the continuous line represents the average number of PD-L1- tumor cells and the shaded area represents its variability, computed across all NTF simulations of each immunogenicity scenario. This graphical representation is generated using MAST.*

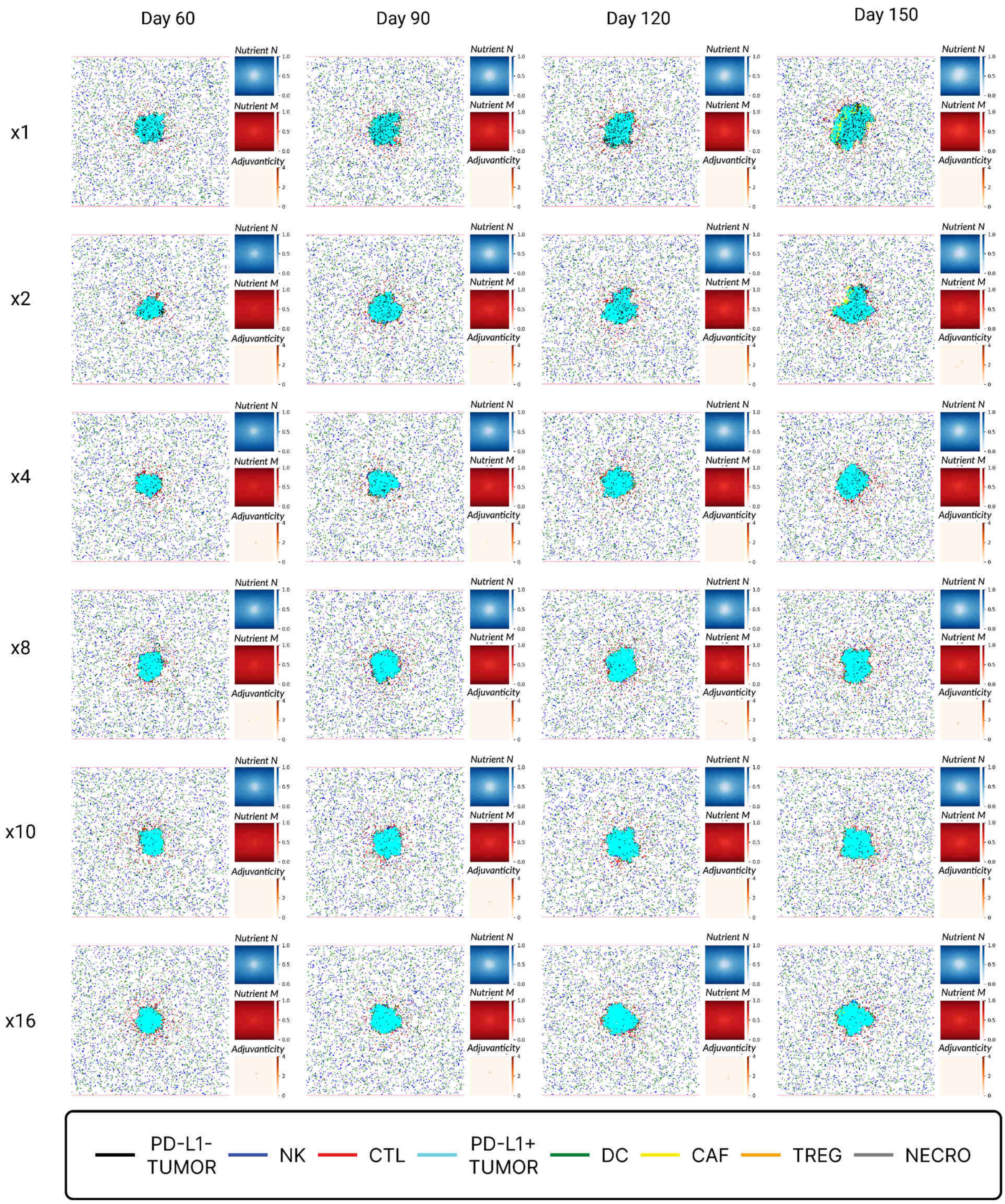

Figure S28 – Spatio-temporal evolution of tumor progression at increasing immunogenicity loss rate in CMS3 scenario. *Spatio-temporal evolution of CMS1 with increased probability of losing immunogenicity. Each row of the grid represents 1 of 100 simulations performed for the specific parameter value. In addition, tumor progression on days 60, 90, 120, and 150 can be observed from left to right. Each panel represents MAST environment and displays: i) in the left, spatial distribution of different agents (legend shows agent-color association); ii) in the right, spatial distribution of nutrient N (e.g., glucose), nutrient M (e.g., oxygen), and adjuvanticity signal respectively from top to bottom subplots. All graphical representations are generated using MAST.*

S6.2.4 Mesenchymal molecular subtype (CMS4)

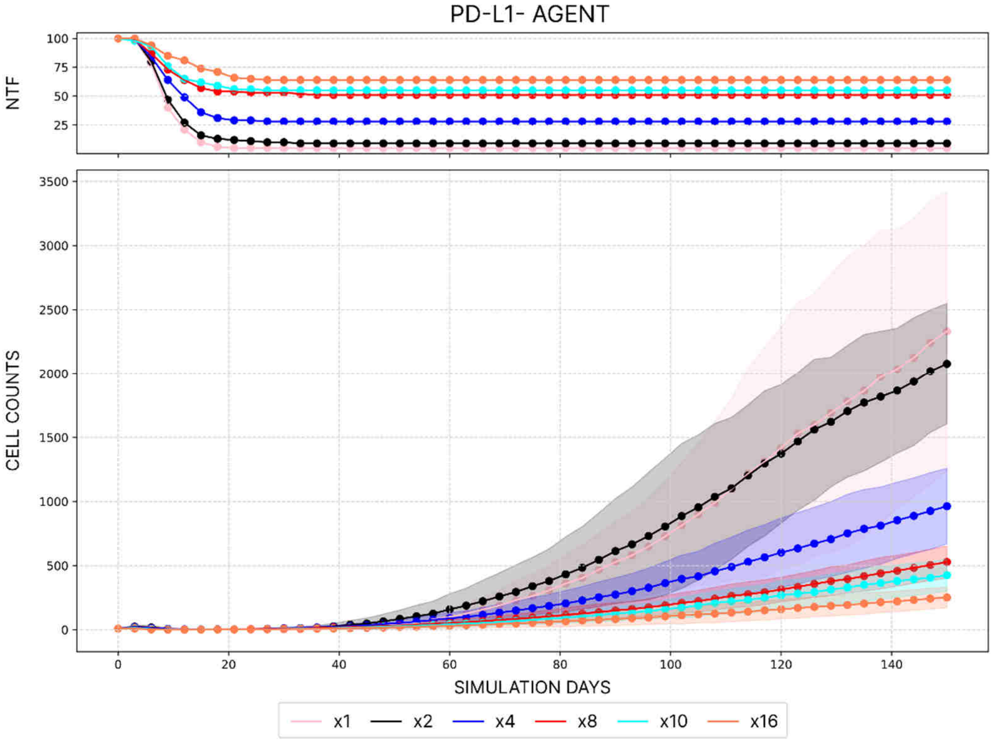

Figure S29 – Cardinality of PD-L1- tumor cells over time at increasing immunogenicity loss rate in CMS4 scenario. *In the upper subgraphs, the number of not completely tumor-free (NTF) simulations in a determined instant (day), i.e., simulations having at least one cancer agent in the domain, is showed for each immunogenicity scenario. In the below subgraph, the continuous line represents the average number of PD-L1- tumor cells and the shaded area represents its variability, computed across all NTF simulations of each immunogenicity scenario. This graphical representation is generated using MAST.*

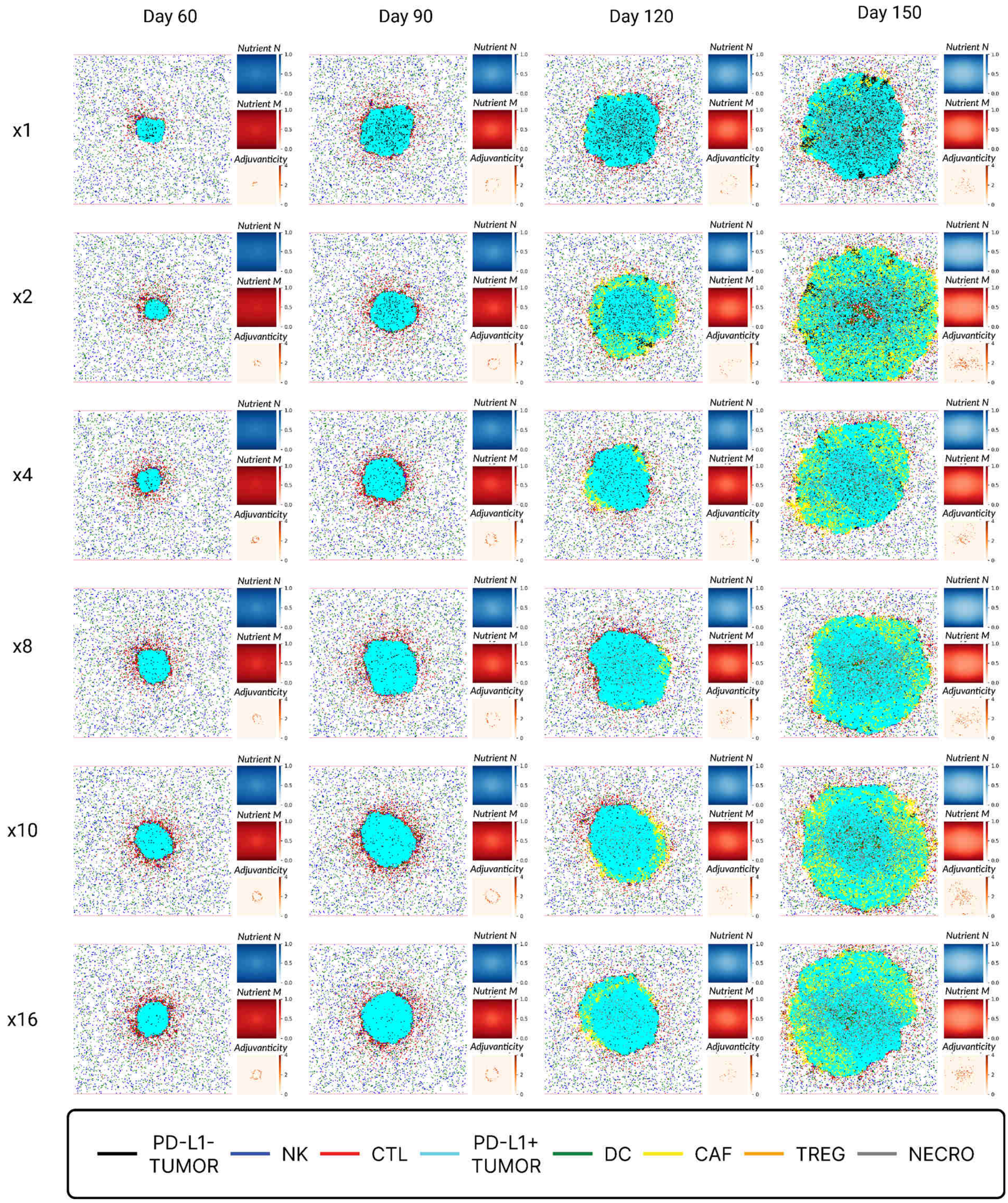

Figure S30 – Spatio-temporal evolution of tumor progression at increasing immunogenicity loss rate in CMS4 scenario. *Spatio-temporal evolution of CMS1 with increased probability of losing immunogenicity. Each row of the grid represents 1 of 100 simulations performed for the specific parameter value. In addition, tumor progression on days 60, 90, 120, and 150 can be observed from left to right. Each panel represents MAST environment and displays: i) in the left, spatial distribution of different agents (legend shows agent-color association); ii) in the right, spatial distribution of nutrient N (e.g., glucose), nutrient M (e.g., oxygen), and adjuvanticity signal respectively from top to bottom subplots. All graphical representations are generated using MAST.*

S6.3 Effect of glucose consumption

In order to investigate the effect of metabolic consumption on tumor progression, we varied *tum_ncons* parameter from the default value for non-tumor cells (i.e.,0.002) of 1-fold, 25-fold, 50-fold, 75-fold, 100-fold and 150-fold. Tumor development in space and time for each CMS scenario are illustrated in Figures S31-38.

We can observe that as the *tum_ncons* parameter increases, it results in a less proliferative microenvironment as PD-L1+ and PD-L1- tumor cell count decrease (Figures S31, S33, S35 and S37). This is expected since tumor requires an increasing amount of nutrient in order to perform duplication, while nutrient availability stay constant. Consequently, the limited nutrient provision leads also to an increasing tumor eradication rate in the in-silico simulations, as shown in the upper subgraphs of Figures S31, S33, S35 and S37.

S6.3.1 Immune molecular subtype (CMS1)

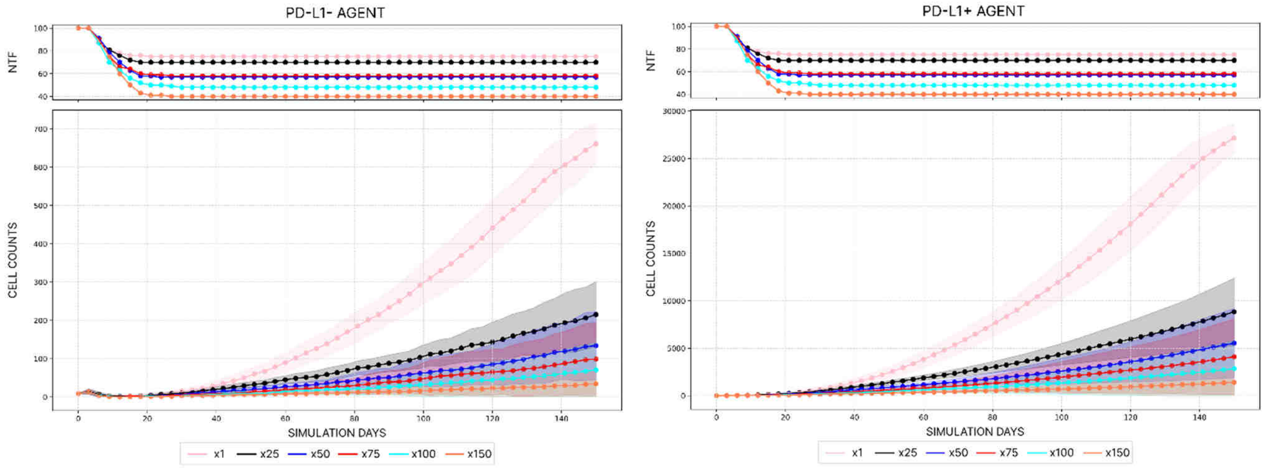

Figure S31 – Cardinality of PD-L1- (left) and PD-L1+ (right) tumor cells over time at increasing metabolic consumption rate in CMS1 scenario. *In the upper subgraphs, the number of not completely tumor-free (NTF) simulations in a determined instant (day), i.e., simulations having at least one cancer agent in the domain, is showed for each metabolic consumption scenario. In the below subgraph, the continuous line represents the average number of PD-L1- (left) and PD-L1+ (right) cells and the shaded area represents its variability, computed across all NTF simulations for each metabolic consumption scenario. This graphical representation is generated using MAST.*

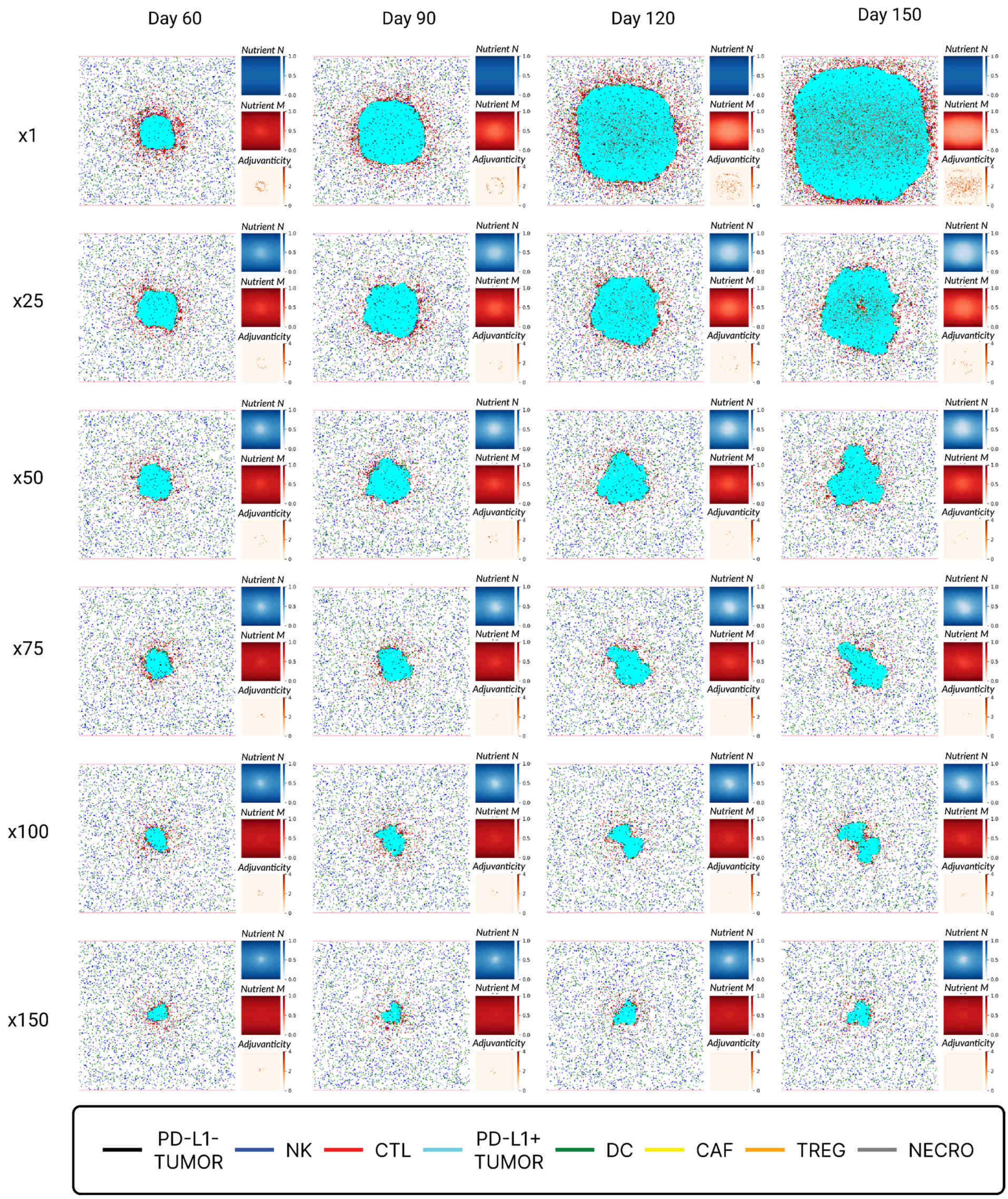

Figure S32 – Spatio-temporal evolution of tumor progression at increasing metabolic consumption rate in CMS1 scenario. *Spatio-temporal evolution of CMS1 with increased nutrient demand. Each row of the grid represents 1 of 100 simulations performed for the specific parameter value. In addition, tumor progression on days 60, 90, 120, and 150 can be observed from left to right. Each panel represents MAST environment and displays: i) in the left, spatial distribution of different agents (legend shows agent-color association); ii) in the right, spatial distribution of nutrient N (e.g., glucose), nutrient M (e.g., oxygen), and adjuvanticity signal respectively from top to bottom subplots. All graphical representations are generated using MAST.*

S6.3.2 Canonical molecular subtype (CMS2)

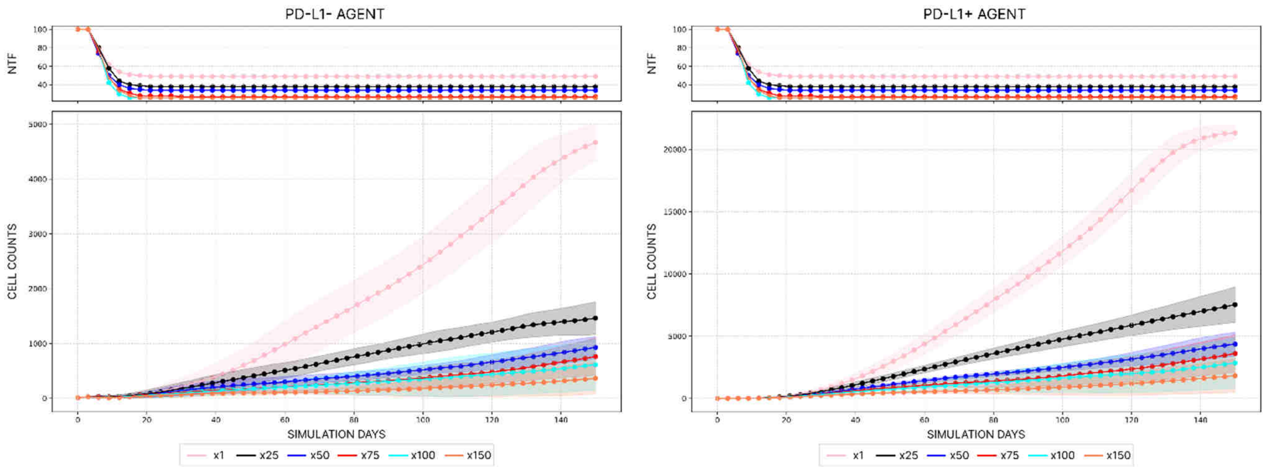

Figure S33 – Cardinality of PD-L1- (left) and PD-L1+ (right) tumor cells over time at increasing metabolic consumption rate in CMS2 scenario. *In the upper subgraphs, the number of not completely tumor-free (NTF) simulations in a determined instant (day), i.e., simulations having at least one cancer agent in the domain, is showed for each metabolic consumption scenario. In the below subgraph, the continuous line represents the average number of PD-L1- (left) and PD-L1+ (right) cells and the shaded area represents its variability, computed across all NTF simulations for each metabolic consumption scenario. This graphical representation is generated using MAST.*

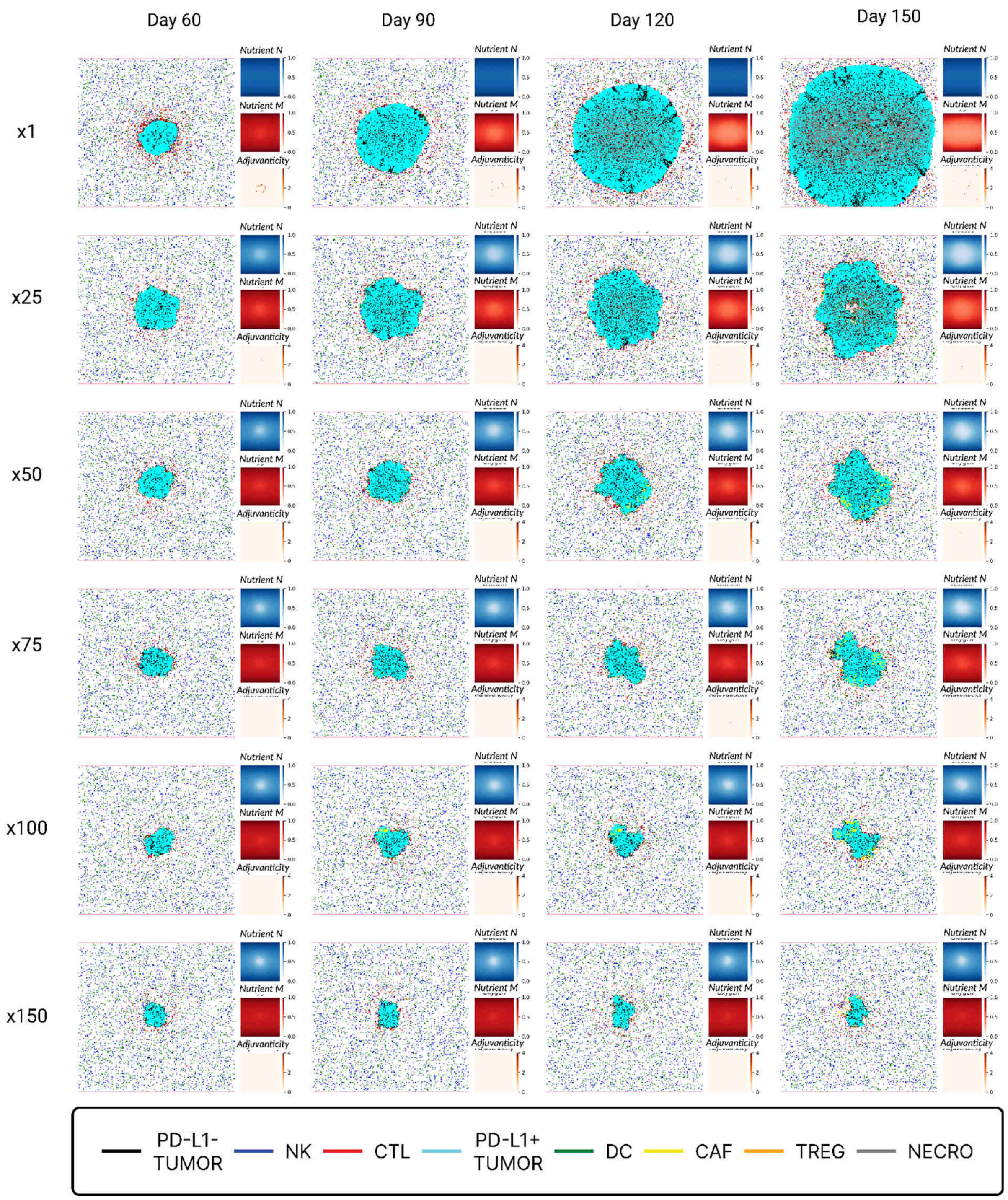

Figure S34 – Spatio-temporal evolution of tumor progression at increasing metabolic consumption rate in CMS2 scenario. *Spatio-temporal evolution of CMS1 with increased nutrient demand. Each row of the grid represents 1 of 100 simulations performed for the specific parameter value. In addition, tumor progression on days 60, 90, 120, and 150 can be observed from left to right. Each panel represents MAST environment and displays: i) in the left, spatial distribution of different agents (legend shows agent-color association); ii) in the right, spatial distribution of nutrient N (e.g., glucose), nutrient M (e.g., oxygen), and adjuvanticity signal respectively from top to bottom subplots. All graphical representations are generated using MAST.*

S6.3.3 Metabolic molecular subtype (CMS3)

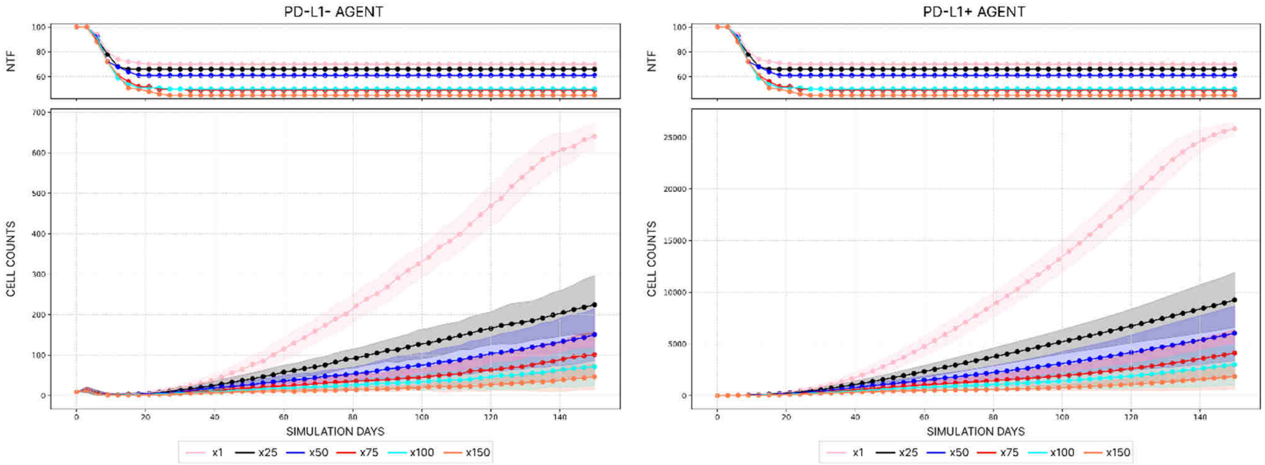

Figure S35 – Cardinality of PD-L1- (left) and PD-L1+ (right) tumor cells over time at increasing metabolic consumption rate in CMS3 scenario. *In the upper subgraphs, the number of not completely tumor-free (NTF) simulations in a determined instant (day), i.e., simulations having at least one cancer agent in the domain, is showed for each metabolic consumption scenario. In the below subgraph, the continuous line represents the average number of PD-L1- (left) and PD-L1+ (right) cells and the shaded area represents its variability, computed across all NTF simulations for each metabolic consumption scenario. This graphical representation is generated using MAST.*

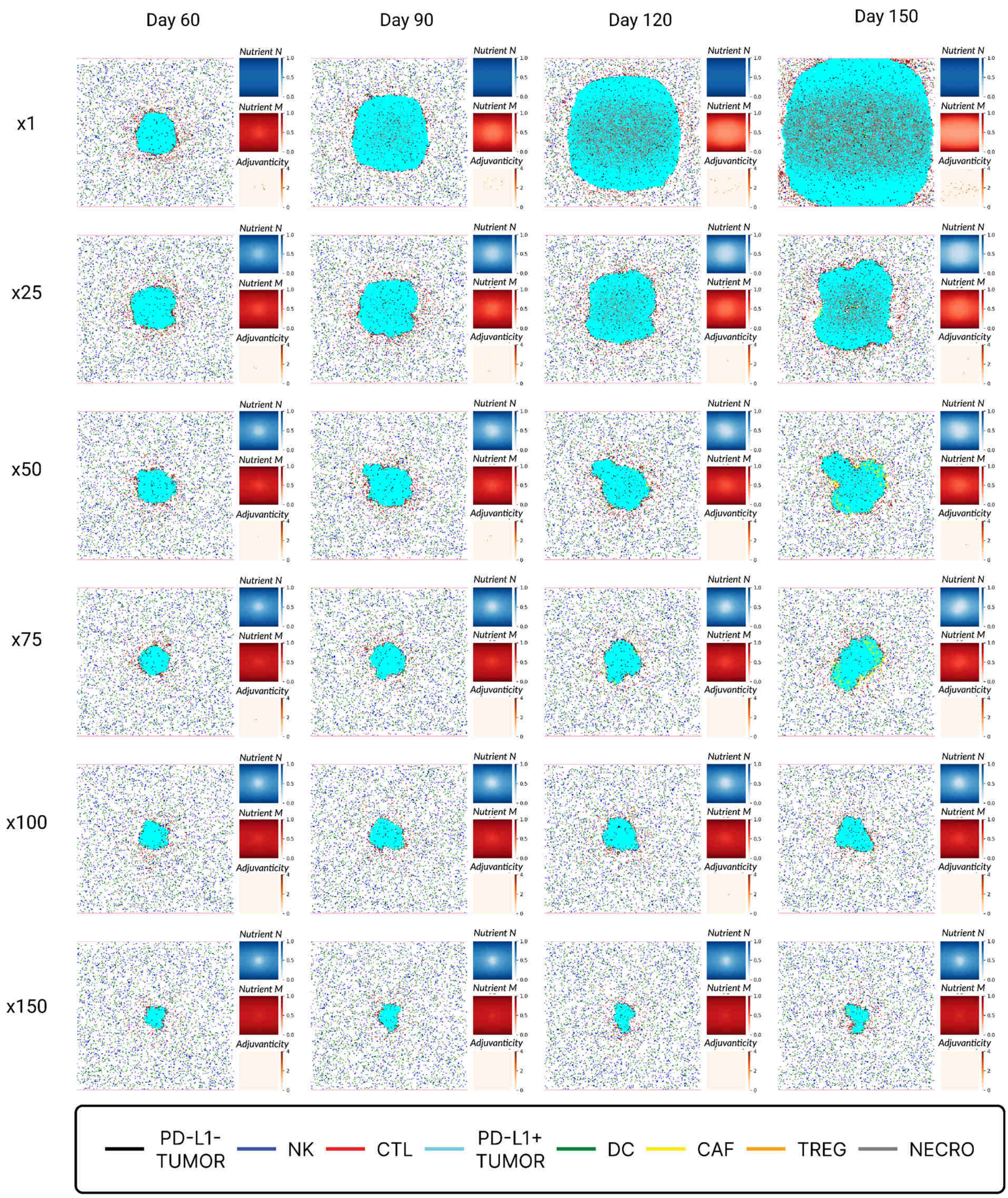

Figure S36– Spatio-temporal evolution of tumor progression at increasing metabolic consumption rate in CMS3 scenario. *Spatio-temporal evolution of CMS1 with increased nutrient demand. Each row of the grid represents 1 of 100 simulations performed for the specific parameter value. In addition, tumor progression on days 60, 90, 120, and 150 can be observed from left to right. Each panel represents MAST environment and displays: i) in the left, spatial distribution of different agents (legend shows agent-color association); ii) in the right, spatial distribution of nutrient N (e.g., glucose), nutrient M (e.g., oxygen), and adjuvanticity signal respectively from top to bottom subplots. All graphical representations are generated using MAST.*

S6.3.4 Mesenchymal molecular subtype (CMS4)

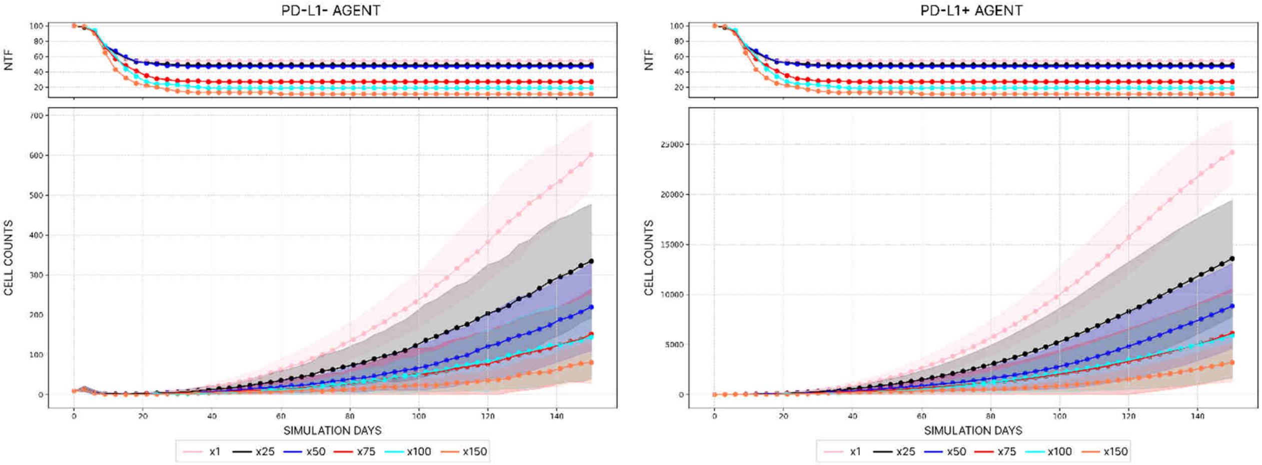

Figure S37 – Cardinality of PD-L1- (left) and PD-L1+ (right) tumor cells over time at increasing metabolic consumption rate in CMS4 scenario. *In the upper subgraphs, the number of not completely tumor-free (NTF) in a determined instant (day), i.e., simulations having at least one cancer agent in the domain, is showed for each metabolic consumption scenario. In the below subgraph, the continuous line represents the average number of PD-L1- (left) and PD-L1+ (right) cells and the shaded area represents its variability, computed across all NTF simulations for each metabolic consumption scenario. This graphical representation is generated using MAST.*

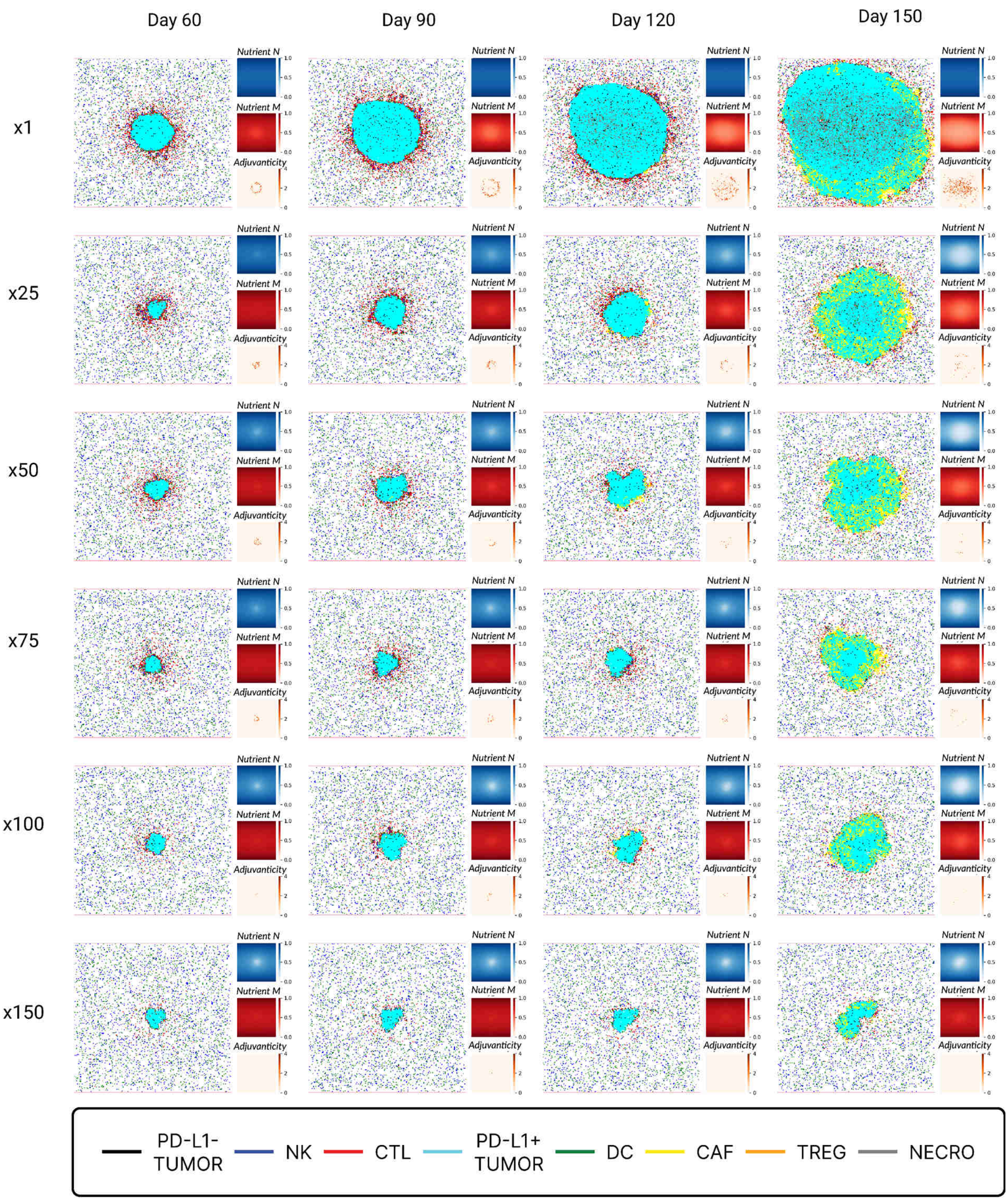

Figure S38 – Spatio-temporal evolution of tumor progression at increasing metabolic consumption rate in CMS4 scenario. *Spatio-temporal evolution of CMS1 with increased nutrient demand. Each row of the grid represents 1 of 100 simulations performed for the specific parameter value. In addition, tumor progression on days 60, 90, 120, and 150 can be observed from left to right. Each panel represents MAST environment and displays: i) in the left, spatial distribution of different agents (legend shows agent-color association); ii) in the right, spatial distribution of nutrient N (e.g., glucose), nutrient M (e.g., oxygen), and adjuvanticity signal respectively from top to bottom subplots. All graphical representations are generated using MAST.*

S6.4 Effect of stromal recruitment

In order to observe the effect of stroma recruitment on tumor progression, we varied *inhib_CAF_recruit_par* parameter from the default value (i.e., 0.1) of 1-fold, 5-fold, 10-fold, 15-fold, 20-fold, and 30-fold. The increasing of this parameter results in a decreasing of CAF recruitment rate in the environment. Tumor development in space and time for each CMS scenario are illustrated in Figures S39-46. We can observe that as the *inhib_CAF_recruit_parameter* parameter increases, it results in a lower recruitment of CAF agents, as expected, and thus in a less proliferative tumor as highlighted by the spatial representation of tumor mass.

S6.4.1 Immune molecular subtype (CMS1)

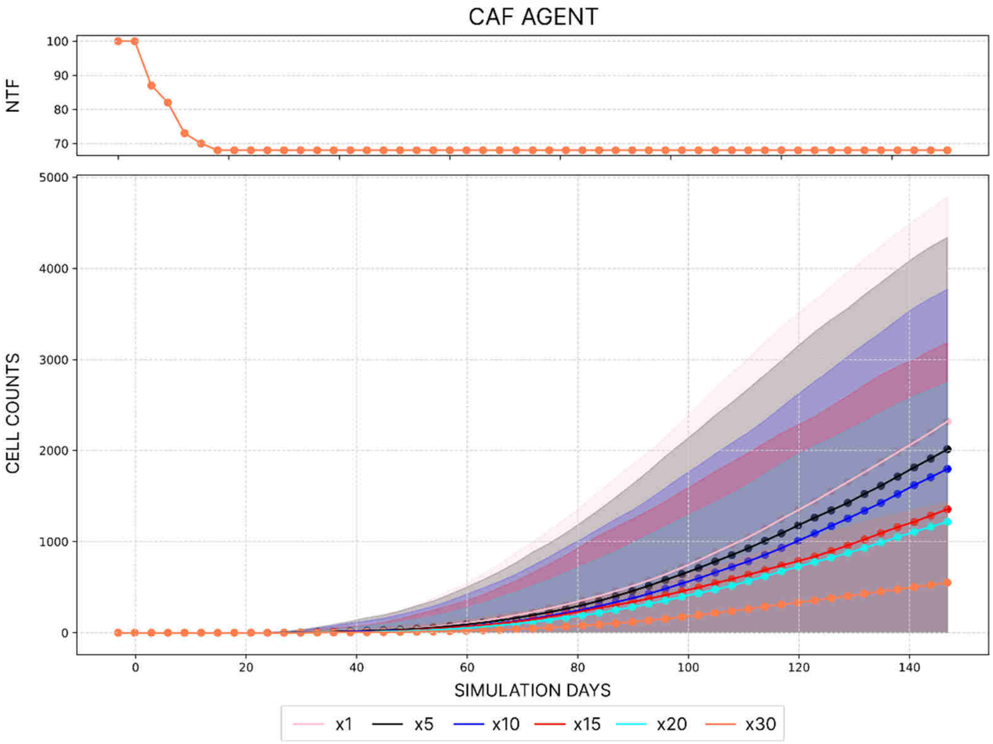

Figure S39 – Cardinality of CAF cells over time at decreasing CAF recruitment rate in CMS1 scenario. *In the upper subgraphs, the number of not completely tumor-free (NTF) simulations in a determined instant (day), i.e., simulations having at least one cancer agent in the domain, is showed for each CAF recruitment parameter setting. In the below subgraph, the continuous line represents the average number of CAF cells, and the shaded area represents its variability, computed across all NTF simulations for each CAF recruitment parameter setting. This graphical representation is generated using MAST.*

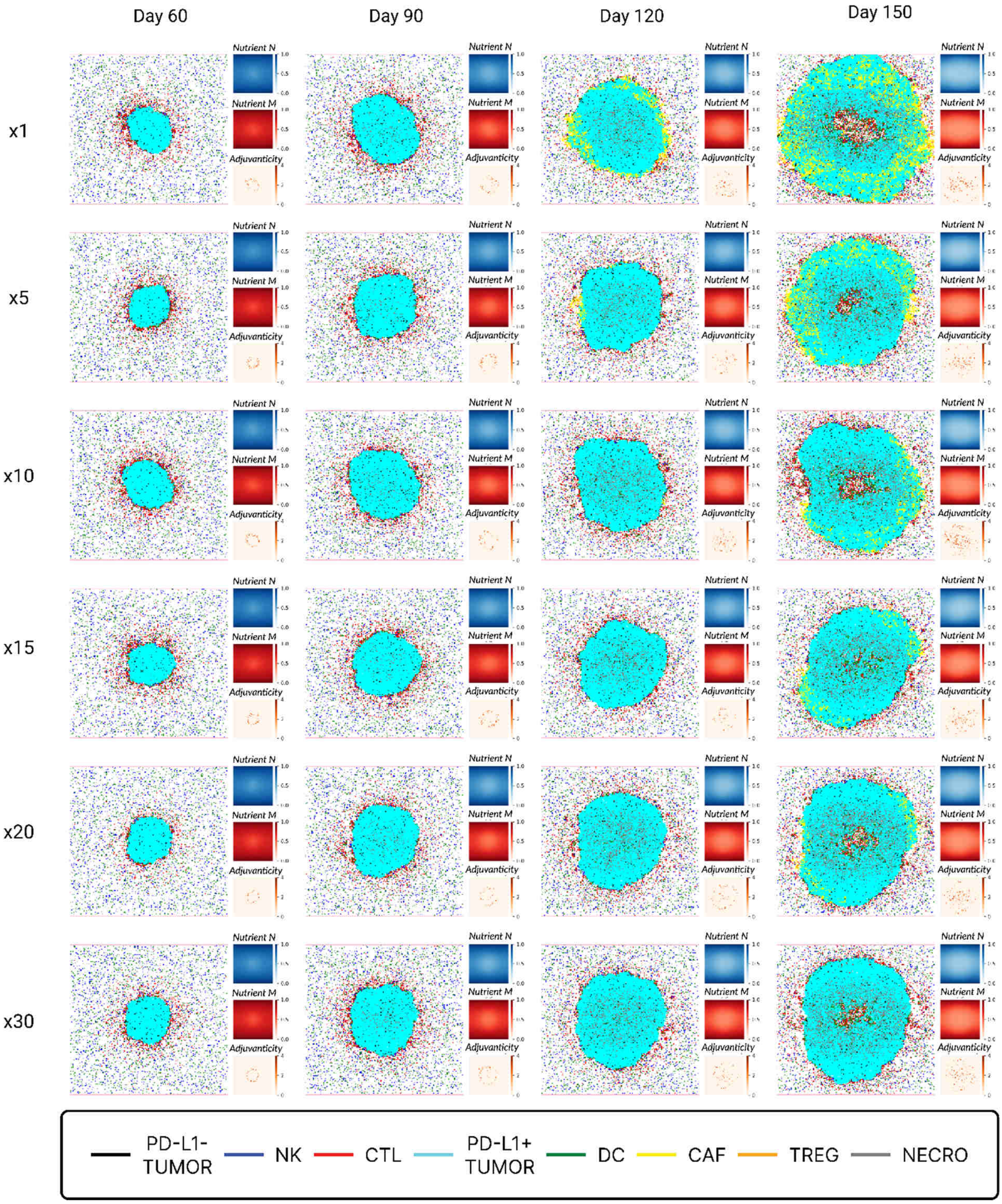

Figure S40 – Spatio-temporal evolution of tumor progression at decreasing CAF recruitment rate in CMS1 scenario. *Spatio-temporal evolution of CMS1 with increased values of inhib_CAF_recruit_par parameter. Each row of the grid represents 1 of 100 simulations performed for the specific parameter value. In addition, tumor progression on days 60, 90, 120, and 150 can be observed from left to right. Each panel represents MAST environment and displays: i) in the left, spatial distribution of different agents (legend shows agent-color association); ii) in the right, spatial distribution of nutrient N (e.g., glucose), nutrient M (e.g., oxygen), and adjuvanticity signal respectively from top to bottom subplots. All graphical representations are generated using MAST.*

S6.4.2 Canonical molecular subtype (CMS2)

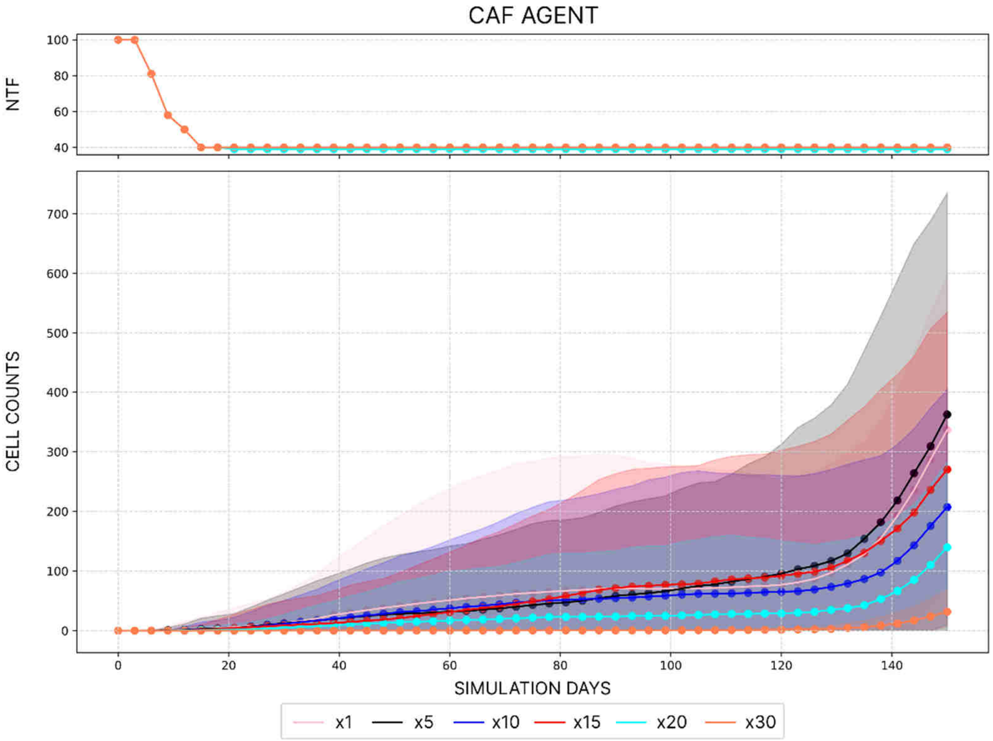

Figure S41 – Cardinality of CAF cells over time at decreasing CAF recruitment rate in CMS2 scenario. *In the upper subgraphs, the number of not completely tumor-free (NTF) simulations in a determined instant (day), i.e., simulations having at least one cancer agent in the domain, is showed for each CAF recruitment parameter setting. In the below subgraph, the continuous line represents the average number of CAF cells, and the shaded area represents its variability, computed across all NTF simulations for each CAF recruitment parameter setting. This graphical representation is generated using MAST.*

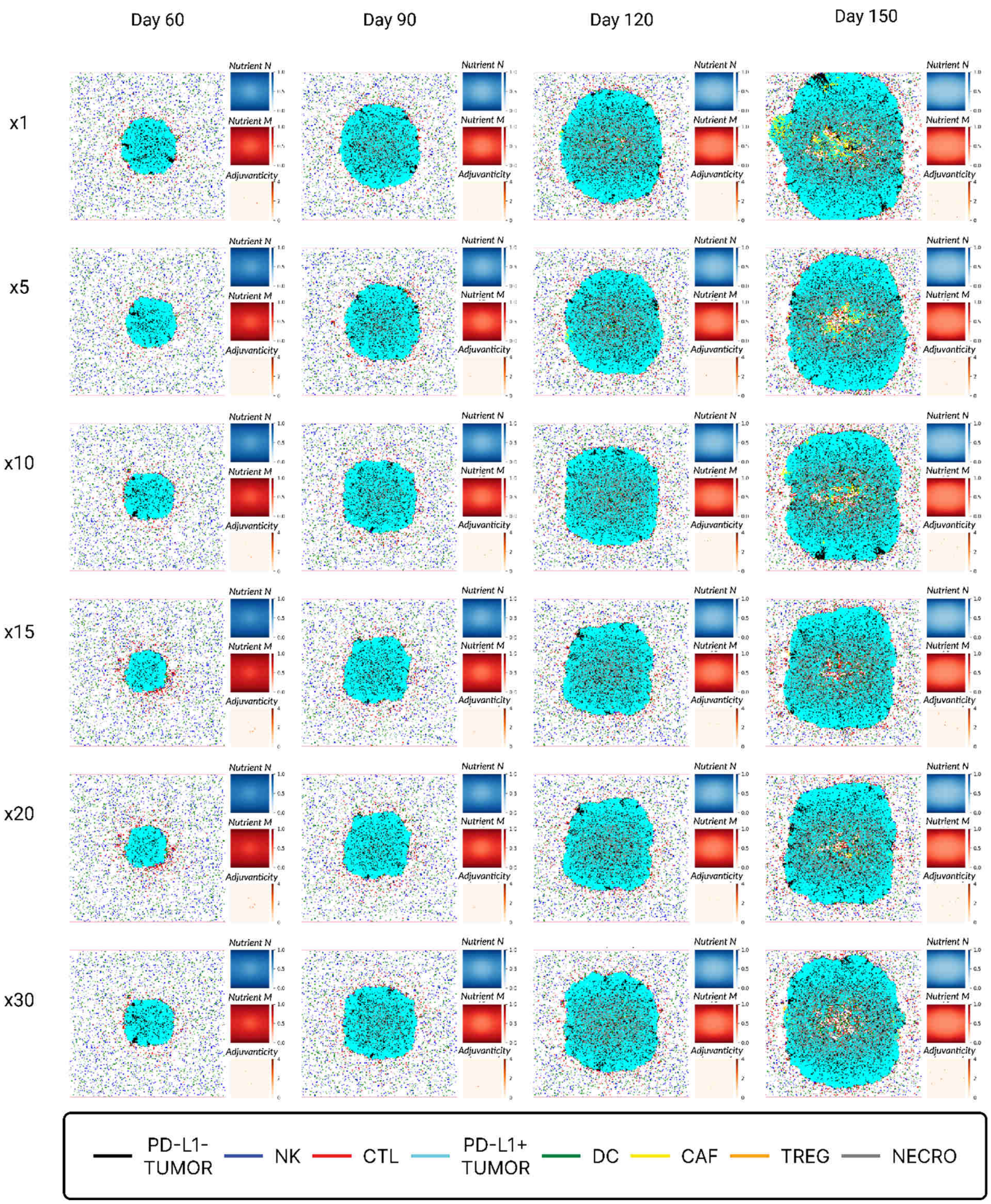

Figure S42 – Spatio-temporal evolution of tumor progression at decreasing CAF recruitment rate in CMS2 scenario. *Spatio-temporal evolution of CMS1 with increased values of inhib_CAF_recruit_par parameter. Each row of the grid represents 1 of 100 simulations performed for the specific parameter value. In addition, tumor progression on days 60, 90, 120, and 150 can be observed from left to right. Each panel represents MAST environment and displays: i) in the left, spatial distribution of different agents (legend shows agent-color association); ii) in the right, spatial distribution of nutrient N (e.g., glucose), nutrient M (e.g., oxygen), and adjuvanticity signal respectively from top to bottom subplots. All graphical representations are generated using MAST.*

S6.4.3 Metabolic molecular subtype (CMS3)

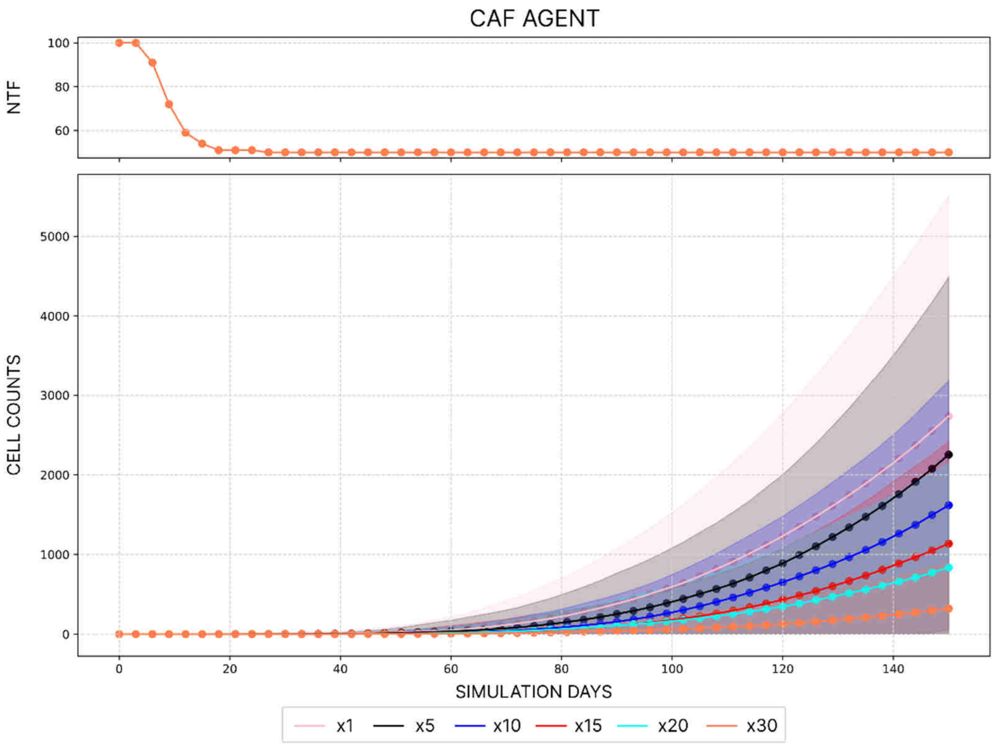

Figure S43 – Cardinality of CAF cells over time at decreasing CAF recruitment rate in CMS3 scenario. *In the upper subgraphs, the number of not completely tumor-free (NTF) simulations in a determined instant (day), i.e., simulations having at least one cancer agent in the domain, is showed for each CAF recruitment parameter setting. In the below subgraph, the continuous line represents the average number of CAF cells, and the shaded area represents its variability, computed across all NTF simulations for each CAF recruitment parameter setting. This graphical representation is generated using MAST.*

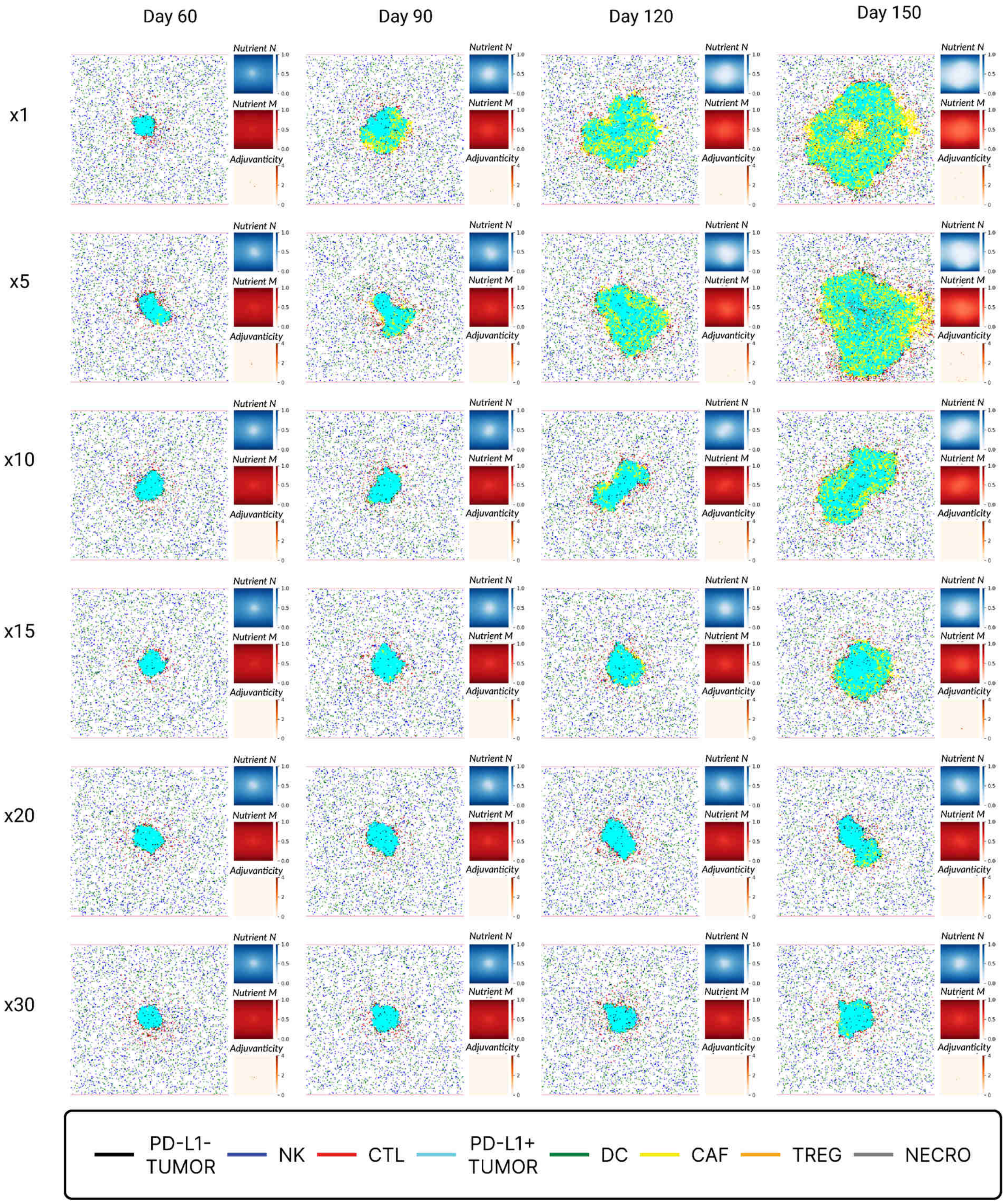

Figure S44 – Spatio-temporal evolution of tumor progression at decreasing CAF recruitment rate in CMS3 scenario. *Spatio-temporal evolution of CMS1 with increased values of inhib_CAF_recruit_par parameter. Each row of the grid represents 1 of 100 simulations performed for the specific parameter value. In addition, tumor progression on days 60, 90, 120, and 150 can be observed from left to right. Each panel represents MAST environment and displays: i) in the left, spatial distribution of different agents (legend shows agent-color association); ii) in the right, spatial distribution of nutrient N (e.g., glucose), nutrient M (e.g., oxygen), and adjuvanticity signal respectively from top to bottom subplots. All graphical representations are generated using MAST.*

S6.4.4 Mesenchymal molecular subtype (CMS4)

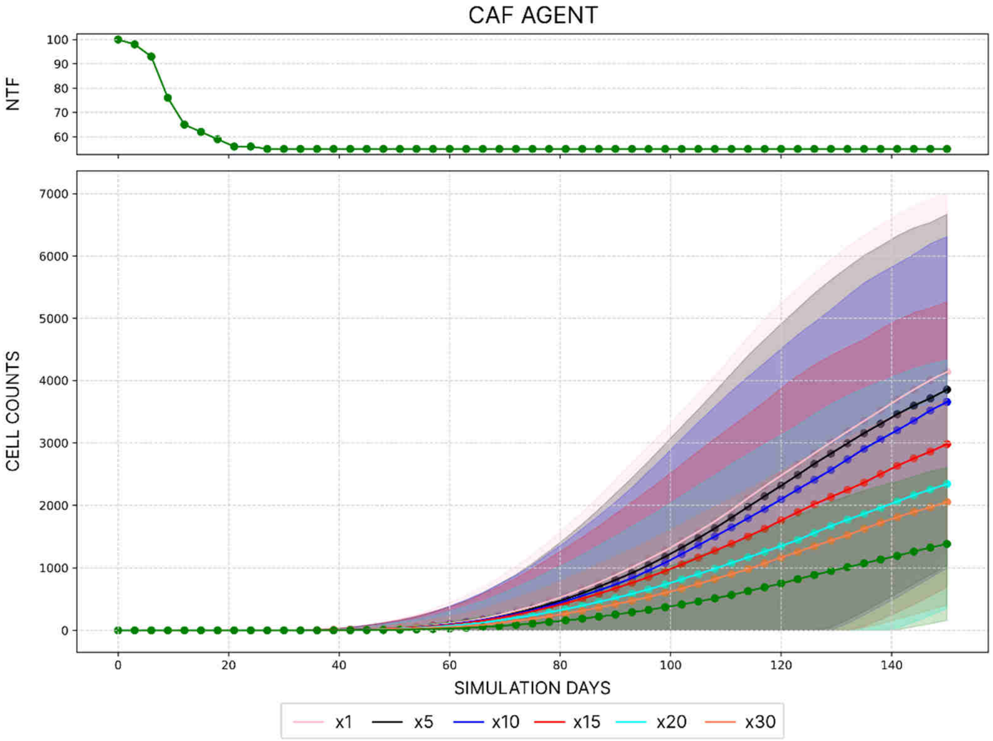

Figure S45 – Cardinality CAF cells over time at decreasing CAF recruitment rate in CMS4 scenario. *In the upper subgraphs, the number of not completely tumor-free (NTF) simulations in a determined instant (day), i.e., simulations having at least one cancer agent in the domain, is showed for each CAF recruitment parameter setting. In the below subgraph, the continuous line represents the average number CAF cells, and the shaded area represents its variability, computed across all NTF simulations for each CAF recruitment parameter setting. This graphical representation is generated using MAST.*

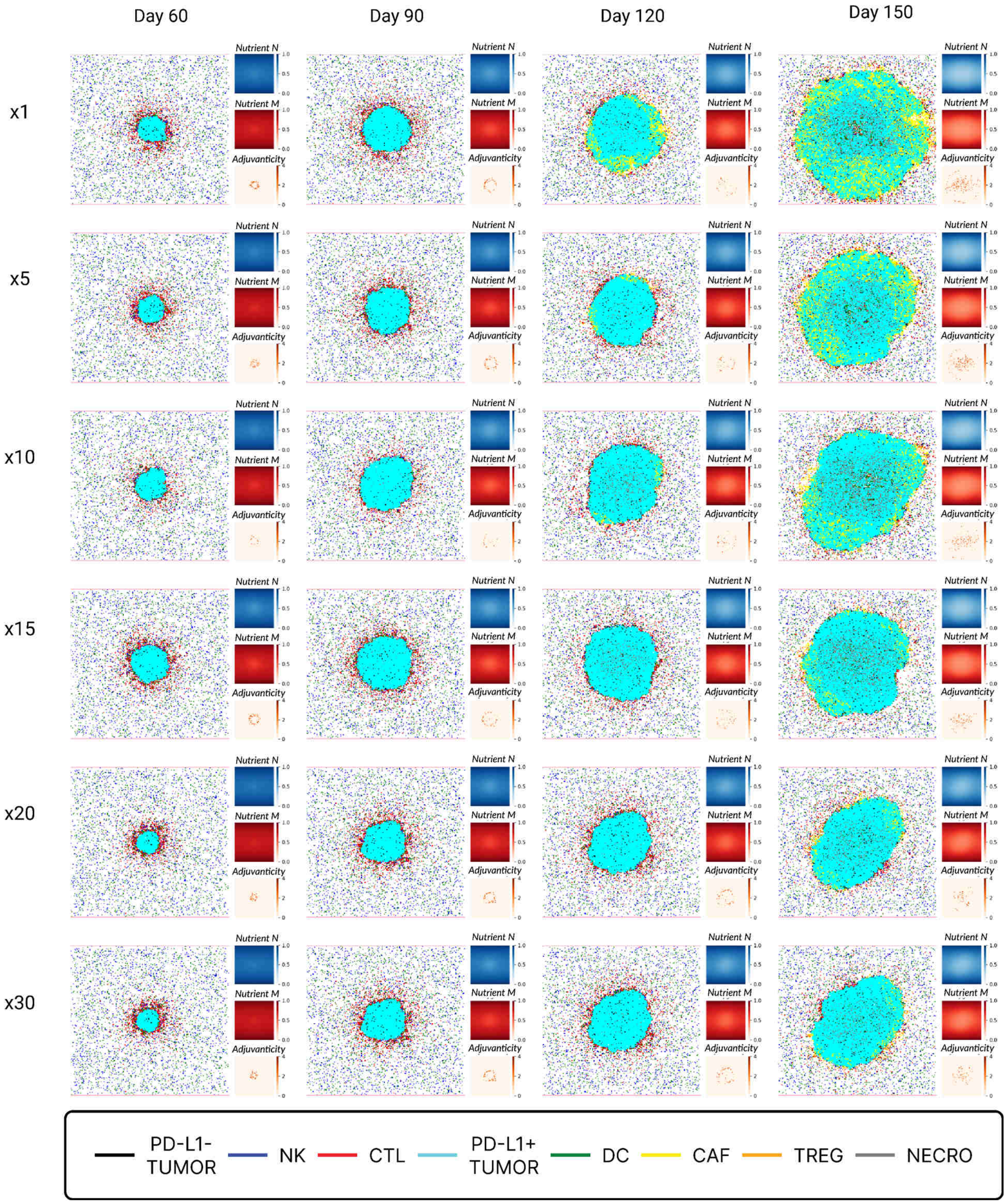

Figure S46 – Spatio-temporal evolution of tumor progression at decreasing CAF recruitment rate in CMS4 scenario. *Spatio-temporal evolution of CMS1 with increased values of inhib_CAF_recruit_par parameter. Each row of the grid represents 1 of 100 simulations performed for the specific parameter value. In addition, tumor progression on days 60, 90, 120, and 150 can be observed from left to right. Each panel represents MAST environment and displays: i) in the left, spatial distribution of different agents (legend shows agent-color association); ii) in the right, spatial distribution of nutrient N (e.g., glucose), nutrient M (e.g., oxygen), and adjuvanticity signal respectively from top to bottom subplots. All graphical representations are generated using MAST.*

Section S7 – Additional information on the effect of data sources on CMS simulation

To exploit the data-driven approach that characterize MAST, we tested the effect of informing parameters in simulating real case scenarios using a different source of information, based on data availability. In particular, data available from TCGA database, i.e.,bulk experiments of genomics and transcriptomics, or only data from SMC dataset of Lee Ho et al., i.e.,bulk genome and single-cell transcriptome experiments are used to inform MAST.
In this section, analysis and results on the effect of considering different sources of information, i.e.,TCGA database or SMC dataset solely, in CMS simulation are provided. In both cases, the emergent properties of CMS still arise from our model, suggesting that different source of information provides specific features of TMEs under investigation, while the relevant and distinct biological characterization of CMS is confirmed.

S7.1 - CMS parameter setting using only TCGA dataset

MAST can be informed using solely data available from TCGA cohort, i.e.,bulk experiments of genomics and transcriptomics. The distribution of TMB and cell fraction values across CMS in TCGA collective has been previously showed in Figures S7, S8 and S9.
Differently from tumor cell–level estimation (Figure S10), average expression level of inhibitory immune checkpoint genes can be computed at tissue-level from bulk RNA-seq of TCGA individuals. The comparison of values between CMS is showed in Figure S47.

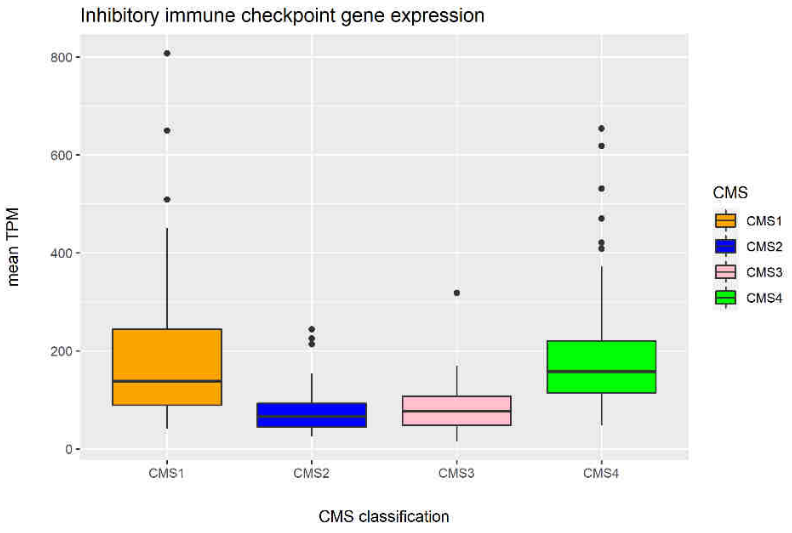

Figure S47 – Distribution of mean average expression level of immune checkpoint genes across CMS computed from bulk RNA-seq in TCGA cohort. *The boxplot describes the median and IQR. Colors identify the different groups: yellow for CMS1, blue for CMS2, pink for CMS3, and green for CMS4.*

By following the trend of metrices distribution across CMS (Figures S7, S8, S9 and S47), the corresponding parameters in MAST are specified for CMS simulating, as showed in Table S4.

**Parameter**	**CMS1**	**CMS2**	**CMS3**	**CMS4**	**Source**	**Dataset**	**Index**
tum_newantigen_rate	High (0.1)	Low (0.01)	Low (0.01)	Low (0.01)	DNA seq	TGCA	TMB
tum_pdlp_rate	High (0.2)	Low (0.02)	Low (0.02)	High (0.2)	RNA-seq	TGCA	Gene expression
tum_adjchange_rate	Low (0.005)	High (0.05)	Mid (0.025)	Low (0.005)	RNA-seq	TGCA	Cell fraction
inhib_TREG_recruit_par	High (1)	High (1)	High (1)	Low (0.1)	RNA-seq	TGCA	Cell fraction
inhib_CAF_recruit_par	High (2)	High (2)	High (2)	Low (0.2)	RNA-seq	TGCA	Cell fraction
tum_ncons	Low (0.02)	Low (0.02)	High (0.2)	Low (0.02)	literature	/	/

Table S4 – Parameter setting of some MAST parameters across CMS using only TCGA dataset as source of information. *The values that differ from the tuning through both TGCA and SMC datasets (Table S3) are highlighted in red.*

With respect to previously setting (Table S3), only parameter related to loss of immunogenicity in CMS3 is changed (highlighted in red in Table S4). Figure S48 shows model outputs of all CMS simulation using this parameter setting. The variation of the parameter affects only model outcomes in CMS3, which results in an increased tumor eradication (i.e., lower height of histograms), while the collective behavior of all tumor groups is still arise by MAST.

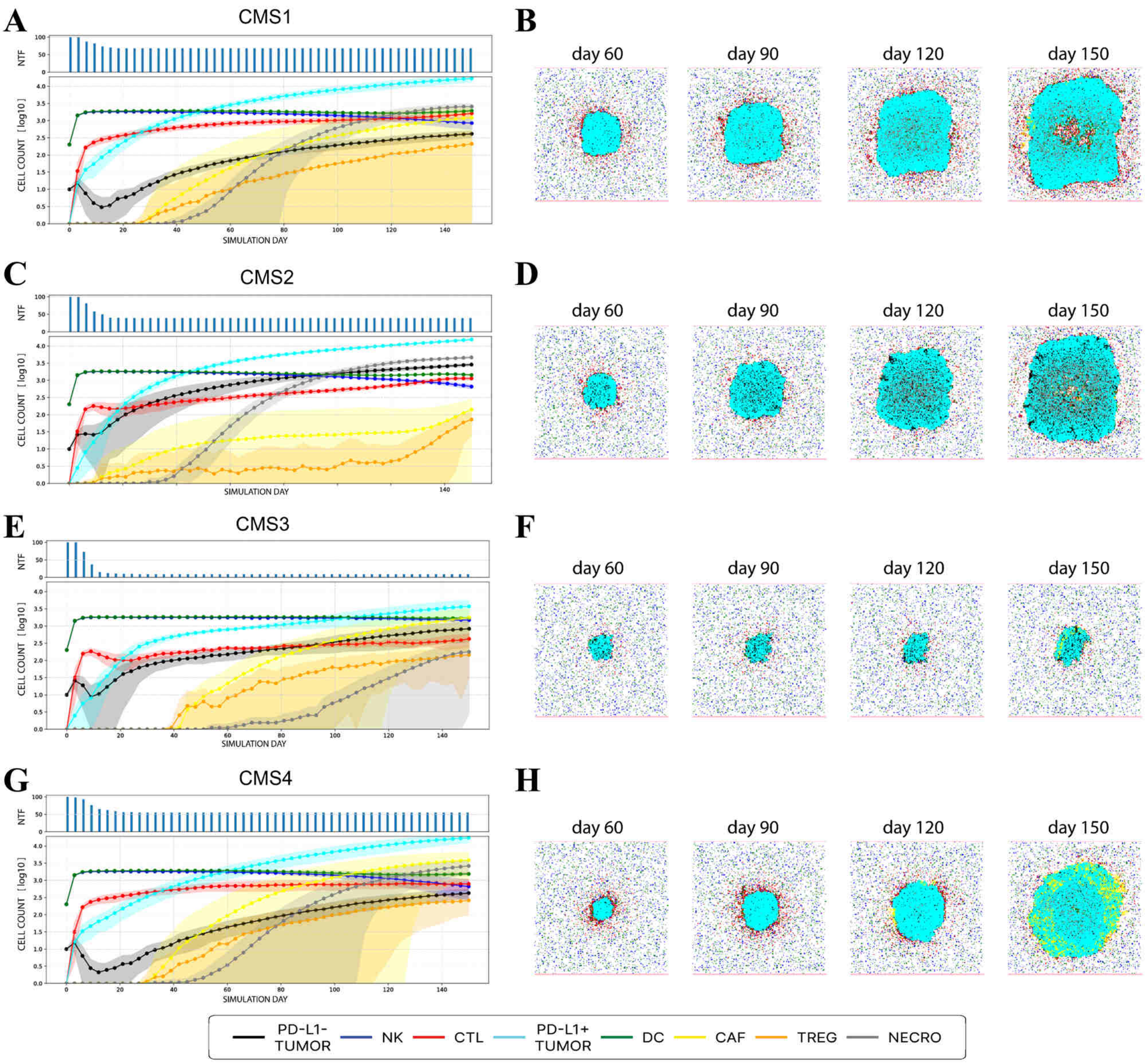

Figure S48 - In silico simulation of the four CMS using only TCGA data. *Left panels (A, C, E, and G) provide information on the temporal evolution of 100 simulations. In the upper subgraph, the number of not completely tumor-free (NTF) simulations in a determined instant (day), i.e., simulations having at least one cancer agent in the domain, is showed for each CMS. In the below subgraph, the time course of agent counts across not completely tumor-free simulations in log10-scale is showed: continuous line represents the average count, and the shaded area represents its variability (± standard deviation). Right panels (B, D, F, and H) display the spatio-temporal evolution of one simulation for each CMS. From left to right, tumor progression on days 60, 90, 120 and 150 are represented. Legend represents color-agent association related to above representations. All graphical representations are generated using MAST.*

S7.2 - CMS parameter setting using only SMC dataset

MAST can be informed using solely data available from SMC dataset, i.e.,bulk experiments of genomics and single-cell transcriptomic experiments.
The distribution of average expression level of PD-L1-like genes at tumor-cell level in SMC dataset was previously showed in Figure S10.
Cell fraction estimates can be computed from single-cell RNA sequencing data using clustering and cell type annotation tools. CAF and Treg cell proportions are used to set parameters related to their recruitment in MAST, while immune cell fraction (including T cells, B cells and myeloid cells) is used to specific mutational probability of creating an immunosuppressive environment and thus locally repelling immune system cells, i.e., *tum_adjchange_rate*. Figures S49 and S50 show their distribution across CMS.

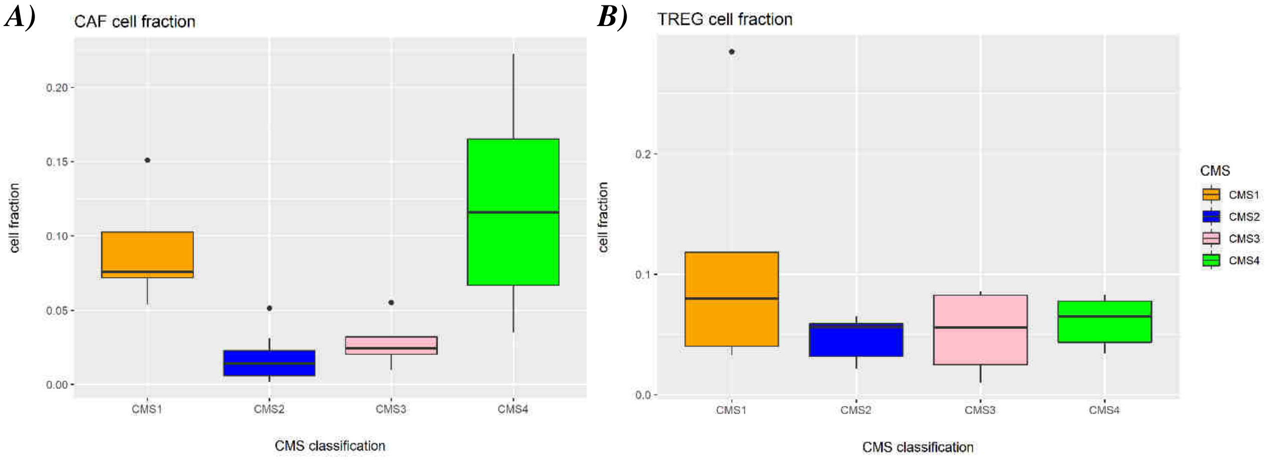

Figure S49 –Distribution of CAF and Treg cell fractions across CMS using single-cell RNA-seq data in SMC dataset. *A) Comparison of CAFs proportions between CMS. B) Comparison of Tregs proportions between CMS. The boxplot describes the median and IQR. Colors identify the different groups: yellow for CMS1, blue for CMS2, pink for CMS3, and green for CMS4.*

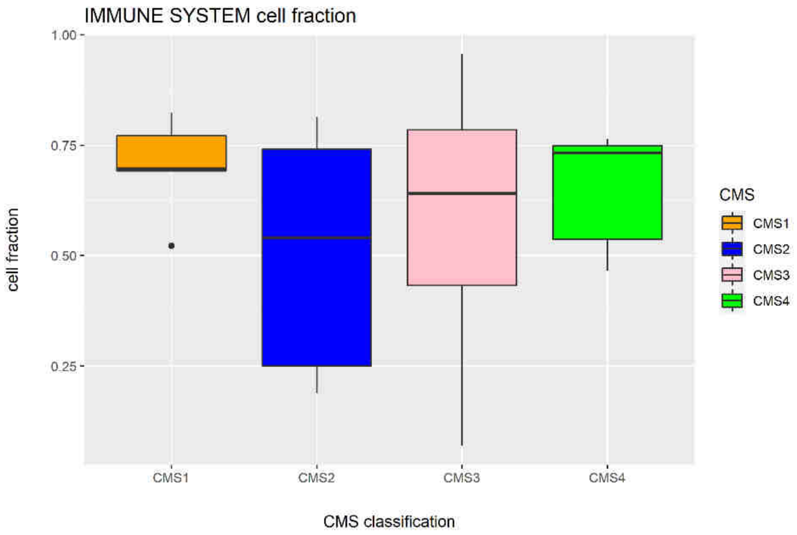

Figure S50 – Distribution of immune system cell fractions across CMS using single-cell RNA-seq data in SMC dataset. *The boxplot describes the median and IQR. Colors identify the different groups: yellow for CMS1, blue for CMS2, pink for CMS3, and green for CMS4.*

The number of mutations for each SMC individual is estimated using bulk whole-genome sequencing, and the relative distribution across CMS is showed Figure S51.

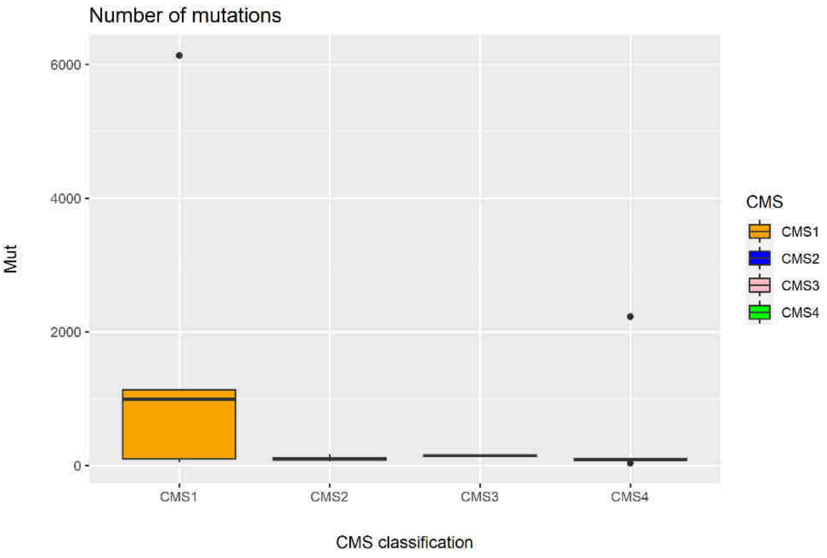

Figure S51 – Distribution of mutational events across CMS from bulk whole-genome sequencing in SMC dataset. *The boxplot describes the median and IQR. Colors identify the different groups: yellow for CMS1, blue for CMS2, pink for CMS3, and green for CMS4.*

By following the trend of metric distributions across CMS derived by SMC dataset (Figures S10, S49, S50 and S51), the corresponding parameters in MAST are specified for CMS simulating, as showed in Table S5.

Parameter	CMS1	CMS2	CMS3	CMS4	Source	Dataset	Index
tum_newantigen_rate	High (0.1)	Low (0.01)	Low (0.01)	Low (0.01)	DNA seq	SMC	TMB
tum_pdlp_rate	High (0.2)	Low (0.02)	High (0.2)	High (0.2)	scRNA-seq	SMC	Gene expression
tum_adjchange_rate	Low (0.005)	High (0.05)	Mid (0.025)	Low (0.005)	scRNA-seq	SMC	Cell fraction
inhib_TREG_recruit_par	Low (0.1)	High (1)	High (1)	High (1)	scRNA-seq	SMC	Cell fraction
inhib_CAF_recruit_par	Low (0.2)	High (2)	High (2)	Low (0.2)	scRNA-seq	SMC	Cell fraction
tum_ncons	Low (0.02)	Low (0.02)	High (0.2)	Low (0.02)	literature	/	/

Table S5 – Parameter setting of some MAST parameters across CMS using only SMC data as source of information. *The values that differ from the tuning through both TGCA and SMC datasets (Table S3) are highlighted in red.*

While *tum_adjchange_rate* and *tum_newantigen_rate* parameter setting is unchanged, parameters related to Treg and CAF recruitment are differently set with respect to the ones of TCGA cohort (highlighted in red in Table S5). In particular, CMS1 and CMS4 settings have slightly changed. Simulation outputs of all CMS are illustrated in Figure S52.

Although an increased spatial recruitment of stromal and inflamed cells which slightly promoted tumor proliferation is observed, the emergent properties of these tumor subtypes are still reproduced by our model.

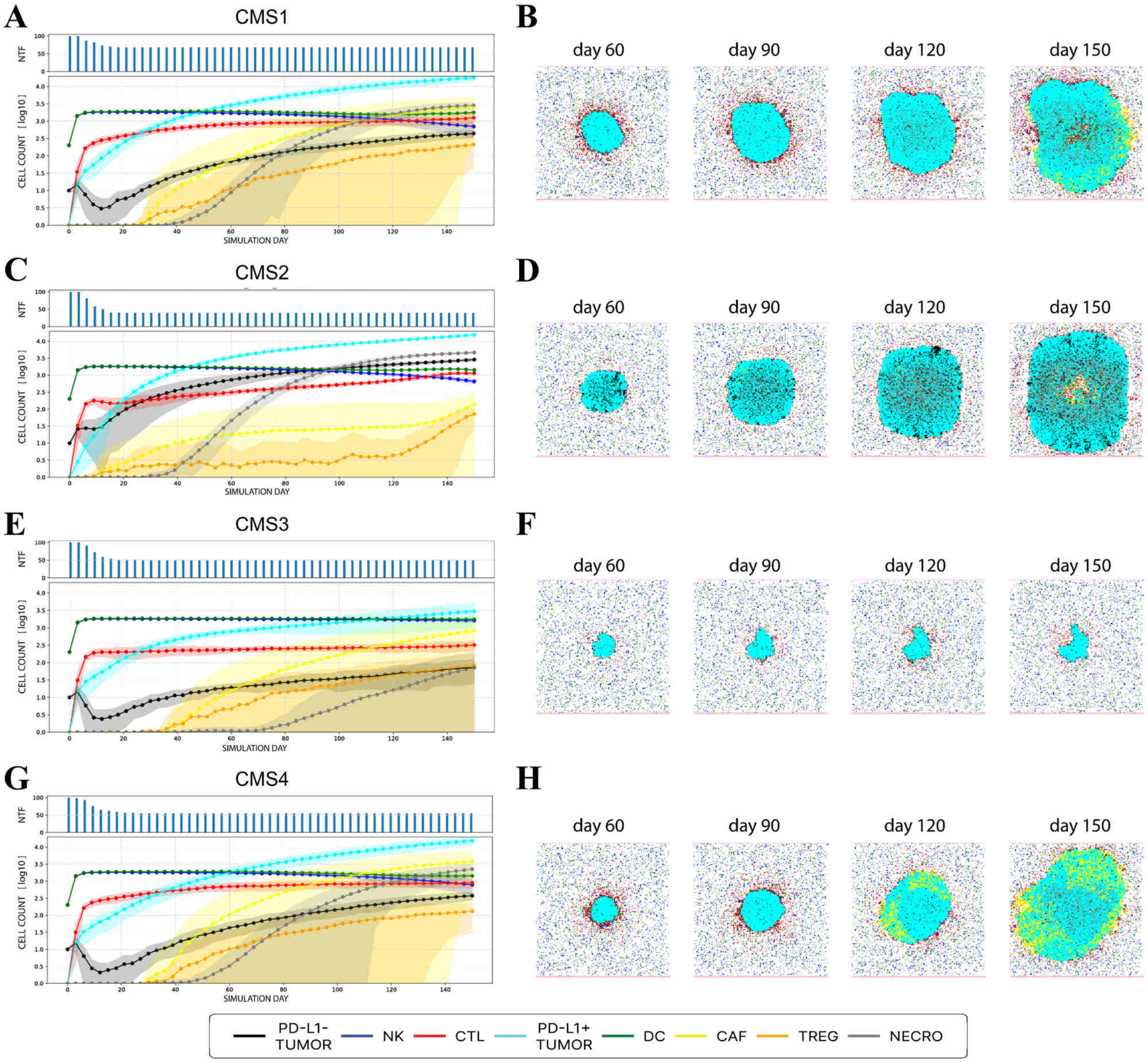

Figure S52 - In silico simulation of the four CMS using only TCGA data. *Left panels (A, C, E, and G) provide information on the temporal evolution of 100 simulations. In the upper subgraph, the number of not completely tumor-free (NTF) simulations in a determined instant (day), i.e., simulations having at least one cancer agent in the domain, is showed for each CMS. In the below subgraph, the time course of agent counts across not completely tumor-free simulations in log10-scale is showed: continuous line represents the average count, and the shaded area represents its variability (± standard deviation). Right panels (B, D, F, and H) display the spatio-temporal evolution of one simulation for each CMS. From left to right, tumor progression on days 60, 90, 120 and 150 are represented. Legend represents color-agent association related to above representations. All graphical representations are generated using MAST.*

Section S8 – Treatment modelling

Among the available cancer treatments, immunotherapy, also known as biological therapy, relies on the knowledge of the tumor microenvironment (Labani-Motlagh *et al.*, 2020). In particular, immune checkpoint inhibitors (ICIs) are used to perform treatment that prevents the immune escape of cancer cells from the IS, targeting immune checkpoint molecules, like PD-1, PD-L1 or Cytotoxic T-Lymphocyte Antigen 4 (CTLA-4). In the literature, some quantitative models attempted to simulate treatments based on immune checkpoint inhibitors to study their effect in the immune system. For example, Gong et al. (Gong *et al.*, 2017) modeled an anti-PD-L1 treatment by reducing the probability of cytotoxic T cells (CD8+) being suppressed by cancer cells bearing a PD-L1+ by a certain factor.
Similarly, MAST allows simulating ICI treatment by increasing the probability of CD8+ cells to kill tumor cells bearing a PD-L1-like mutation, thus reproducing the effect of blocking PD-L1-like molecules. To mimic the periodic intravenous administration of ICI-based drugs for a specific amount of time (e.g., until disease progression or patient death) (Makaremi *et al.*, 2021). MAST implements a basic infrastructure to define treatments time schedules. Specifically, tumor development begins without any treatment and the immunotherapy can be optionally applied starting at a certain time point and specifying the frequency of the treatment and its duration.
For each delivery day (inject_cure), we simulate the ICI treatment for the number of days specified as duration (drug_duration). In this way, different drug schedule can be simulated in the in-silico domain. In this preliminary implementation, the dosage of the administered drug remains constant throughout the window; this feature will be made adjustable in the future to emulate possible different dosages over time.
Our simulations (injections at days 15/45/105 for 9 days duration and probability of CD8+ cells to kill tumor cells bearing a PD-L1-like mutation increased at 45%) showed that early application of treatment provides overall better outcomes on CMS1 and CMS4 increasing the percentage of simulations with complete remission of 3 folds and 2 folds respectively with respect to CMS2 and CMS3 where it increases the percentage of complete remissions of 1.1 and 1.5 folds, respectively. These results suggest that ICI treatment is effective in MSI immune scenario. This is confirmed in the literature, where CMS1 is known to be the main target of ICI treatment, whereas CMS2 and CMS3 do not benefit from this kind of treatment (Hoorn *et al.*, 2022; Hu *et al.*, 2021; Le *et al.*, 2017). CMS4 scenario is moderately responsive to in silico simulated treatment accordingly to what has been suggested in literature, since CMS4 tumors might benefit more from combinations of different targeted therapies compared to ICI treatment alone (Dienstmann *et al.*, 2018; Huyghe *et al.*, 2020).

Supplementary References

Aran,D. *et al.* (2017) xCell: Digitally portraying the tissue cellular heterogeneity landscape. *Genome Biol*, **18**, 1–14.

Becht,E. *et al.* (2016) Estimating the population abundance of tissue-infiltrating immune and stromal cell populations using gene expression. *Genome Biol*, **17**, 1–20.

Breart,B. *et al.* (2008) Two-photon imaging of intratumoral CD8+ T cell cytotoxic activity during adoptive T cell therapy in mice. *J Clin Invest*, **118**, 1390–1397.

Carvalho,S. *et al.* (2017) Glucose diffusion in colorectal mucosa-a comparative study between normal and cancer tissues. *J Biomed Opt*, **22**, 091506.

Cerignoli,F. *et al.* (2018) In vitro immunotherapy potency assays using real-time cell analysis. *PLoS One*, **13**, e0193498.

Christophe,C. *et al.* (2015) A Biased Competition Theory of Cytotoxic T Lymphocyte Interaction with Tumor Nodules. *PLoS One*, **10**, e0120053.

Dienstmann,R. *et al.* (2018) Molecular Subtypes and the Evolution of Treatment Decisions in Metastatic Colorectal Cancer. *American Society of Clinical Oncology Educational Book*, 231–238.

Donini,C. *et al.* (2018) Next generation immune-checkpoints for cancer therapy. *J Thorac Dis*, **10**, S1581–S1601.

Eide,P.W. *et al.* (2017) CMScaller: an R package for consensus molecular subtyping of colorectal cancer pre-clinical models. *Scientific Reports 2017 7:1*, **7**, 1–8.

Fessler,E. and Medema,J.P. (2016) Colorectal Cancer Subtypes: Developmental Origin and Microenvironmental Regulation. *Trends Cancer*, **2**, 505–518.

Finotello,F. *et al.* (2019) Molecular and pharmacological modulators of the tumor immune contexture revealed by deconvolution of RNA-seq data. *Genome Med*, **11**, 1–20.

Finotello,F. and Trajanoski,Z. (2018) Quantifying tumor-infiltrating immune cells from transcriptomics data. *Cancer Immunology, Immunotherapy*, **67**, 1031–1040.

Gong,C. *et al.* (2017) A computational multiscale agent-based model for simulating spatio-temporal tumour immune response to PD1 and PDL1 inhibition. *J R Soc Interface*, **14**.

Grossman,R.L. *et al.* (2016) Toward a Shared Vision for Cancer Genomic Data. *New England Journal of Medicine*, **375**, 1109–1112.

Guinney,J. *et al.* (2015) The consensus molecular subtypes of colorectal cancer. *Nature Medicine 2015 21:11*, **21**, 1350–1356.

Hoorn,S. ten *et al.* (2022) Clinical Value of Consensus Molecular Subtypes in Colorectal Cancer: A Systematic Review and Meta-Analysis. *JNCI: Journal of the National Cancer Institute*, **114**, 503–516.

Huang,C. *et al.* (2019) Immune checkpoint molecules. Possible future therapeutic implications in autoimmune diseases. *J Autoimmun*, **104**, 102333.

Hu,F. *et al.* (2021) Comprehensive Analysis of Subtype-Specific Molecular Characteristics of Colon Cancer: Specific Genes, Driver Genes, Signaling Pathways, and Immunotherapy Responses. *Front Cell Dev Biol*, **9**, 3089.

Huyghe,N. *et al.* (2020) Immunotherapy with immune checkpoint inhibitors in colorectal cancer: what is the future beyond deficient mismatch-repair tumours? *Gastroenterol Rep (Oxf)*, **8**, 11–24.

Jimenez-Sanchez,A. *et al.* (2019) Comprehensive benchmarking and integration of tumor microenvironment cell estimation methods. *Cancer Res*, **79**, 6238–6246.

Kather,J.N. *et al.* (2018) Genomics and emerging biomarkers for immunotherapy of colorectal cancer. *Semin Cancer Biol*, **52**, 189–197.

Kather,J.N. *et al.* (2017) In silico modeling of immunotherapy and stroma-targeting therapies in human colorectal cancer. *Cancer Res*, **77**, 6442–6452.

Labani-Motlagh,A. *et al.* (2020) The Tumor Microenvironment: A Milieu Hindering and Obstructing Antitumor Immune Responses. *Front Immunol*, **11**, 940.

Lapuente-Santana,Ó. *et al.* (2021) Interpretable systems biomarkers predict response to immune-checkpoint inhibitors. *Patterns*, **2**, 100293.

Le,D.T. *et al.* (2017) Mismatch repair deficiency predicts response of solid tumors to PD-1 blockade. *Science (1979)*, **357**, 409–413.

Lee,H.O. *et al.* (2020) Lineage-dependent gene expression programs influence the immune landscape of colorectal cancer. *Nat Genet*, **52**, 594–603.

Li,B. *et al.* (2016) Comprehensive analyses of tumor immunity: Implications for cancer immunotherapy. *Genome Biol*, **17**, 1–16.

Liu,J. *et al.* (2018) An Integrated TCGA Pan-Cancer Clinical Data Resource to Drive High-Quality Survival Outcome Analytics. *Cell*, **173**, 400-416.e11.

Makaremi,S. *et al.* (2021) Immune Checkpoint Inhibitors in Colorectal Cancer: Challenges and Future Prospects. *Biomedicines*, **9**.

Peng,Q. *et al.* (2020) PD-L1 on dendritic cells attenuates T cell activation and regulates response to immune checkpoint blockade. *Nature Communications 2020 11:1*, **11**, 1–8.

Picard,E. *et al.* (2020) Relationships Between Immune Landscapes, Genetic Subtypes and Responses to Immunotherapy in Colorectal Cancer. *Front Immunol*, **11**, 369.

Qin,S. *et al.* (2019) Novel immune checkpoint targets: moving beyond PD-1 and CTLA-4. *Molecular Cancer 2019 18:1*, **18**, 1–14.

Racle,J. *et al.* (2017) Simultaneous enumeration of cancer and immune cell types from bulk tumor gene expression data. *Elife*, **6**.

Robert,C. (2020) A decade of immune-checkpoint inhibitors in cancer therapy. *Nature Communications 2020 11:1*, **11**, 1–3.

Sturm,G. *et al.* (2019) Comprehensive evaluation of transcriptome-based cell-type quantification methods for immuno-oncology. *Bioinformatics*, **35**, i436–i445.

Thorsson,V. *et al.* (2018) The Immune Landscape of Cancer. *Immunity*, **48**, 812-830.e14.

Weinstein,J.N. *et al.* (2013) The Cancer Genome Atlas Pan-Cancer analysis project. *Nature Genetics 2013 45:10*, **45**, 1113–1120.

Wiedemann,A. *et al.* (2006) Cytotoxic T lymphocytes kill multiple targets simultaneously via spatiotemporal uncoupling of lytic and stimulatory synapses. *Proc Natl Acad Sci U S A*, **103**, 10985–10990.

Yalcin,A. *et al.* (2009) Regulation of glucose metabolism by 6-phosphofructo-2-kinase/fructose-2,6-bisphosphatases in cancer. *Exp Mol Pathol*, **86**, 174–179.
